# Supplementary material for: K27-linked ubiquitination of BRAF by ITCH engages cytokine response to maintain MEK-ERK signaling
Source: Nat Commun. 2019 Apr 23;10:1870. doi: 10.1038/s41467-019-09844-0 (PMC6478693; doi:10.1038/s41467-019-09844-0)
Supplement: Supplementary file 1 — Supplementary Infomation [file 41467_2019_9844_MOESM1_ESM.pdf]

Supplementary Information

K27-linked Ubiquitination of BRAF by ITCH Engages Cytokine Response to Maintain MEK-ERK Signaling

Yin et al.

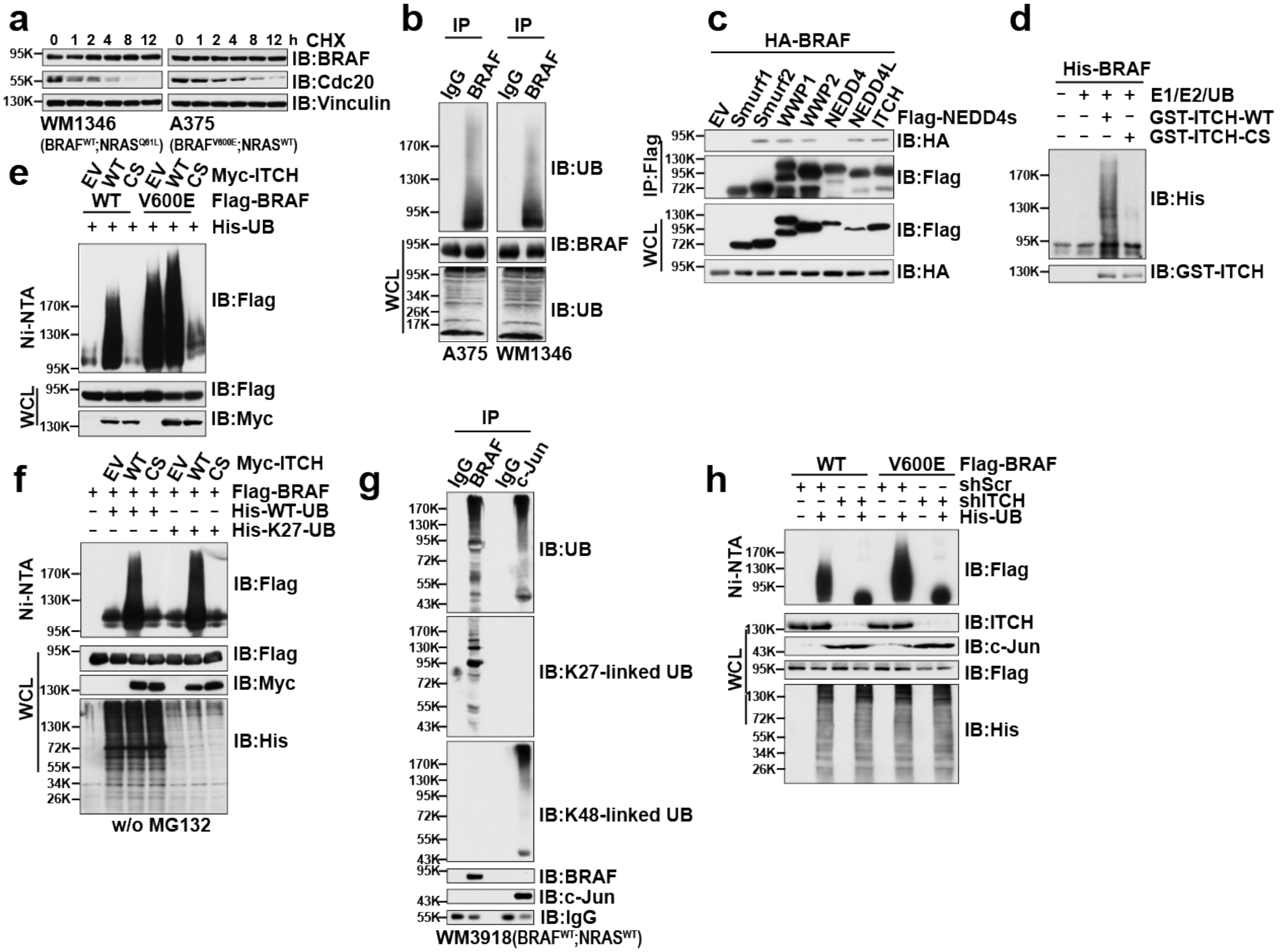

Supplementary Figure 1. BRAF is Poly-ubiquitinated by ITCH via the K27-linkage

(a) The half-life of BRAF was determined in WM1346 and A375 cells. 20 μg·ml^{-1} cycloheximide (CHX) was added to cells for the indicated time periods before harvest for immunoblot (IB) analysis.

(b) Endogenous BRAF is ubiquitinated in melanoma cells. IB analysis of whole cell lysates (WCL) and anti-BRAF immunoprecipitates (IP) derived from A375 and WM1346 cells (IgG was used as a negative control).

(c) BRAF binds to a subset of NEDD4 family E3 ligases. IB analysis of WCL and anti-Flag IP derived from 293T cells transfected with HA-BRAF and the indicated Flag-NEDD4 constructs.

(d) ITCH promoted BRAF ubiquitination *in vitro*. Bacterial purified His-BRAF protein was incubated with the indicated GST-tagged WT- or C832S(CS)-ITCH proteins as well as E1, E2 and ubiquitin proteins at 30[illegible] C for 60 minutes before being resolved by SDS-PAGE and probed with the anti-His antibody.

(e) ITCH promoted ubiquitination of both WT- and V600E-BRAF. IB analysis of WCL and Ni-NTA affinity precipitates derived from 293T cells transfected with His-UB and the indicated Flag-BRAF and Myc-ITCH constructs. Cells were pretreated with 10 μM MG132 for 12 hours before harvest.

(f) BRAF was poly-ubiquitinated via the K27-linkage. IB analysis of WCL and Ni-NTA affinity precipitates derived from 293T cells transfected with Flag-BRAF and the indicated Myc-ITCH, His-WT-UB or His-K27-UB constructs.

(g) Endogenous BRAF and c-Jun ubiquitination was determined using anti-ubiquitin, anti-K27-linkage and anti-K48-linkage antibodies. IB analysis of WCL and anti-BRAF or anti-c-Jun IP derived from WM3918 cells treated with 10 μM MG132 for 12 hours and 50 ng/·ml^{-1} TNFα for 20 min.

(h) Depletion of ITCH abolished poly-ubiquitination of both BRAF^{WT} and BRAF^{V600E}. IB analysis of WCL and Ni-NTA affinity precipitates derived from shScr-293T or sh*ITCH*-293T cells transfected with His-UB and the indicated Flag-BRAF constructs. Cells were pretreated with 10 μM MG132 for 12 hours before harvest.

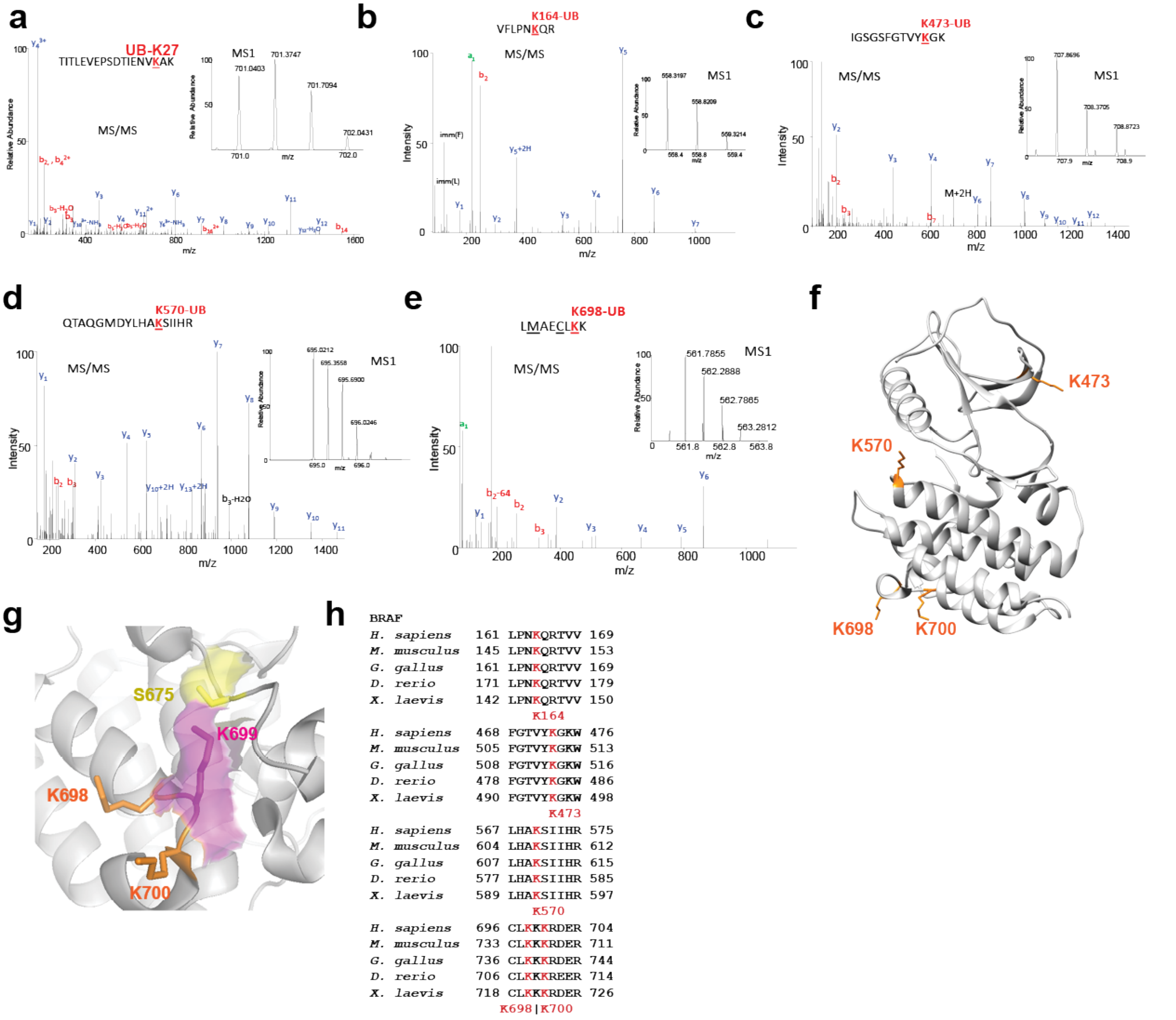

Supplementary Figure 2. Identification of BRAF Ubiquitination Sites by Mass Spectrometry

(a) Tandem mass spectrum identifying the K27-linkage on the purified ubiquitinated-BRAF protein samples. 293T cells were transfected with Flag-BRAF and HA-UB constructs. 48 hours post-transfection, cells were lysed for anti-Flag IP. The immuno-purified Flag-BRAF was eluted using Flag peptide (Sigma-Aldrich) and subjected to anti-HA IP to enrich ubiquitinated BRAF proteins, which were then subjected to mass spectrometry analyses of ubiquitin linkage type.

(b-e) Tandem mass spectrum identifying K164 (**b**), K473 (**c**), K570 (**d**) and K698 (**e**) as major BRAF ubiquitination residues in cells. 293T cells were transfected with Flag-BRAF and HA-UB constructs. 48 hours post-transfection, cells were lysed for anti-Flag IP. The immuno-purified Flag-BRAF was eluted using Flag peptide (Sigma-Aldrich) and subjected to anti-HA IP to enrich ubiquitinated BRAF proteins, which were then subjected to mass spectrometry analyses of ubiquitination sites.

(f-g) Structural illustrations of the identified lysine residues in a reported BRAF kinase domain crystal structure [1] (PDB ID: 1UWH). The structures were generates using UCSF Chimera.

(h) Sequence alignments of the ubiquitinated lysine-containing regions of BRAF proteins from various species.

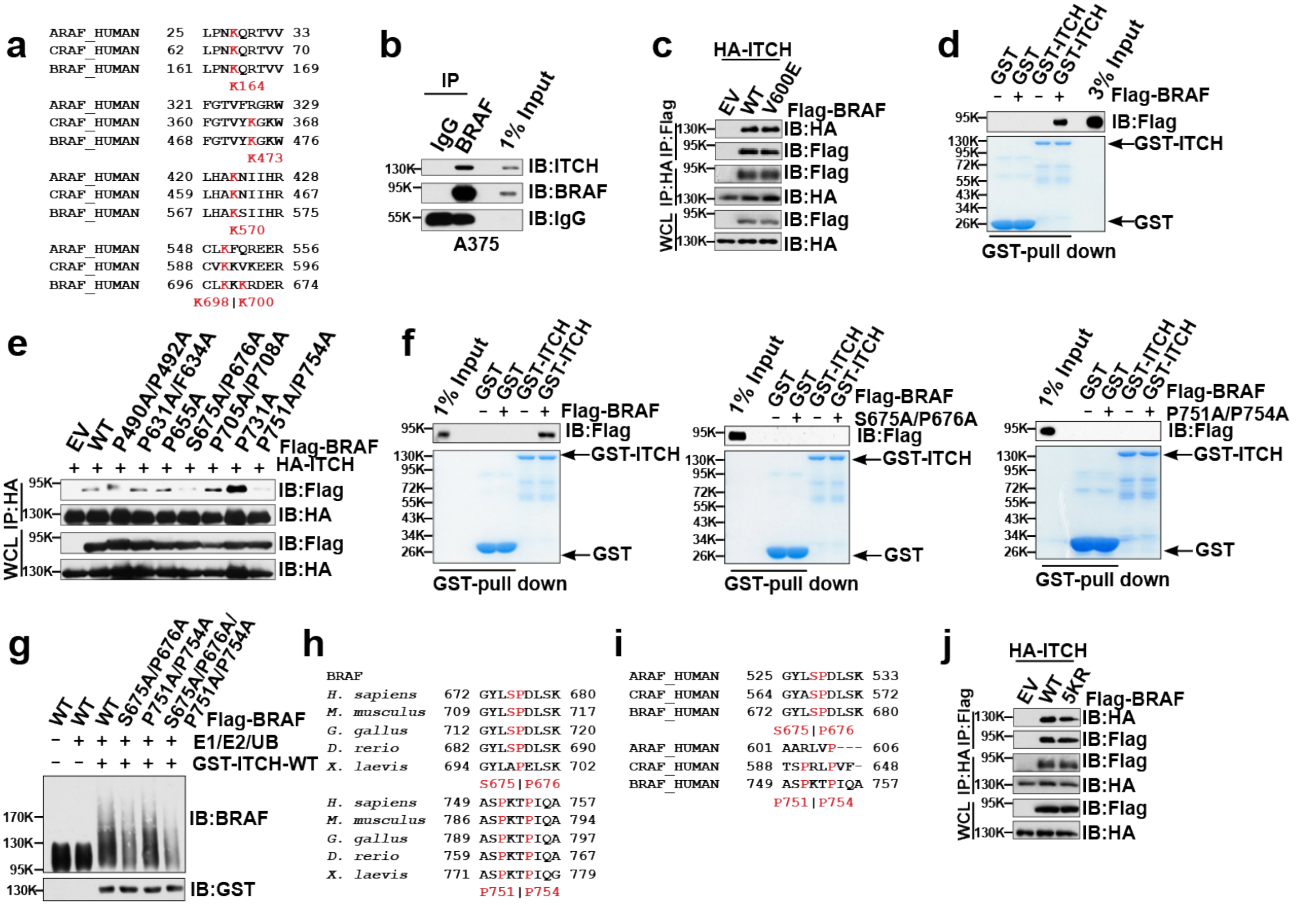

Supplementary Figure 3. ITCH Binds to BRAF via Two Proline-rich Motifs

(a) Sequence alignments of the protein sequences from three RAF isoforms flanking the five lysine residues found in BRAF that are ubiquitinated.

(b) Endogenous BRAF bound to ITCH. IB analysis of WCL and anti-BRAF IP derived from A375 cells.

(c) Both WT-BRAF and V600E-BRAF bound to ITCH. Immunoblot (IB) analysis of whole cell lysates (WCL) and immunoprecipitates (IP) derived from 293T cells transfected with HA-ITCH and the indicated Flag-BRAF constructs.

(d) BRAF bound to purified recombinant GST-ITCH, but not the GST.

(e) Mutation of two proline-rich motifs in BRAF attenuated its binding to ITCH in cells. IB analysis of WCL and anti-HA IP derived from 293T cells transfected with HA-ITCH and the indicated Flag-BRAF constructs.

(f) Mutation of two proline-rich motifs in BRAF attenuated its binding to ITCH *in vitro*. GST-pull down experiments using purified recombinant GST-ITCH proteins showed specific interaction with WT-BRAF but not S675A/P676A-BRAF or P751A/P754A-BRAF.

(g) BRAF S675A/P676A and P751A/P754A mutants displayed compromised ubiquitination *in vitro*. Various Flag-BRAF WT and KR mutants immuno-purified from 293T cells were incubated with bacterially purified GST-ITCH and the E1, E2 and ubiquitin proteins as indicated. The reaction was performed at 30°C for 60 minutes and followed by SDS-PAGE and IB analyses.

(h) Sequence alignments of the S675/P676- and the P751/P754-containing regions of BRAF proteins from various species.

(i) Sequence alignments of the protein sequences from three RAF isoforms flanking the two identified ITCH interacting motifs found in BRAF.

(j) Ubiquitination-deficient 5KR-BRAF bound to ITCH. IB analysis of WCL and IP derived from 293T cells transfected with HA-ITCH and the indicated Flag-BRAF constructs.

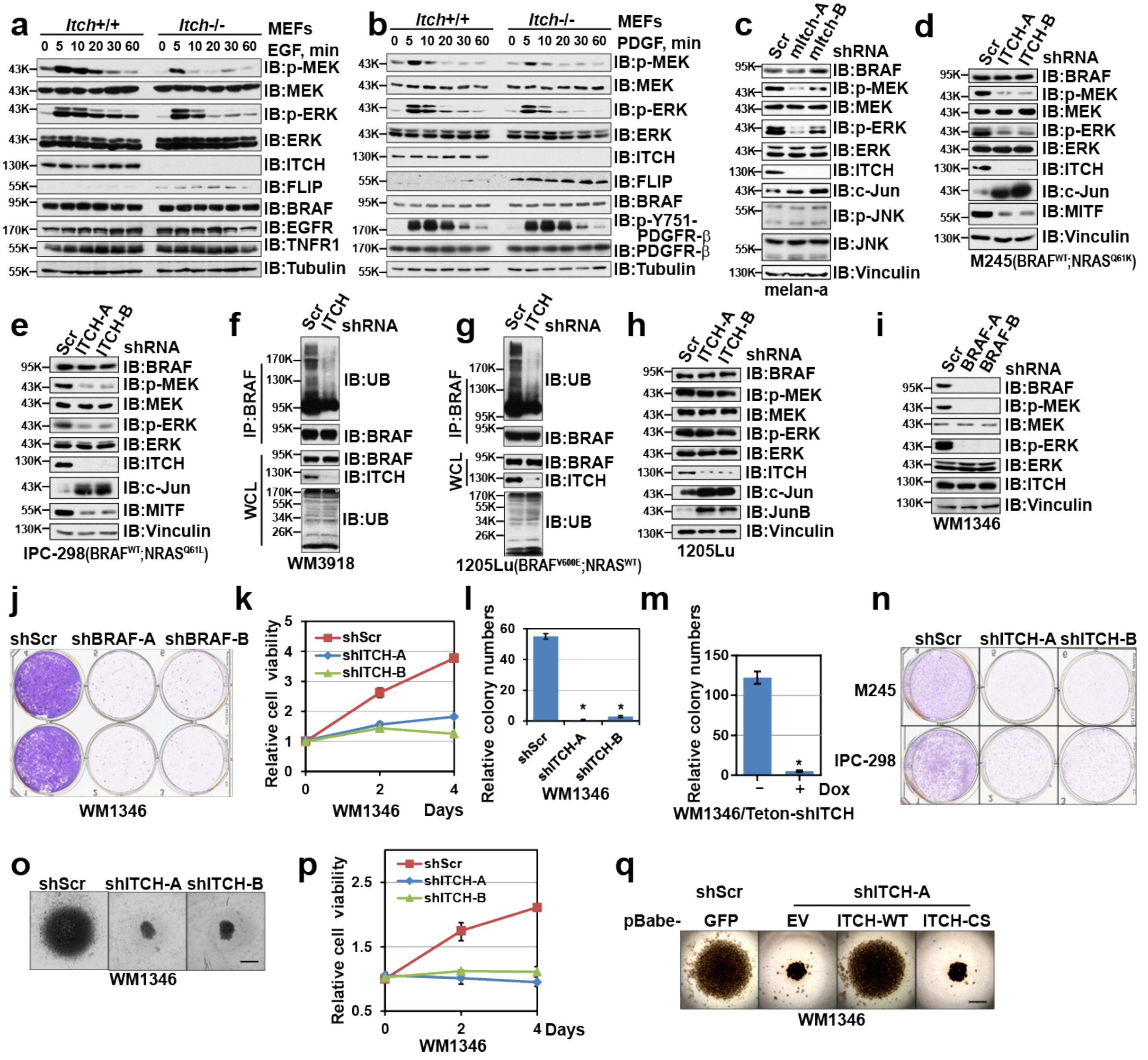

Supplementary Figure 4. Depletion of ITCH Attenuates BRAF Activity and Suppresses Melanoma Cell Growth

(a-b) Immunoblot (IB) analysis of whole cell lysates (WCL) derived from WT and *Itch*$^{-/-}$ MEFs treated with 10 ng·ml^{-1} EGF (**a**) or 4 ng·ml^{-1} PDGF (**b**) after overnight serum starvation for the indicated time period.

(c) IB analysis of WCL and derived from melan-a cells infected with the indicated sh*Itch* lentiviral constructs, a scrambled shRNA construct (shScr) was used as the negative control.

(d-e) IB analysis of WCL derived from M245 (**d**) or IPC-298 (**e**) cells infected with the indicated sh*ITCH* lentiviral constructs, shScr was used as the negative control.

(f-g) Depletion of ITCH abolished endogenous BRAF ubiquitination. IB analysis of WCL and anti-BRAF immunoprecipitates (IP) derived from WM3918 (**f**) and 1205Lu (**g**) cells infected with shScr or sh*ITCH* lentiviral construct.

(h) IB analysis of WCL derived from 1205Lu cells infected with the indicated sh*ITCH* lentiviral constructs, shScr was used as the negative control.

(i) IB analysis of WCL derived from WM1346 cells infected with the indicated sh*BRAF* lentiviral constructs, shScr was used as the negative control.

(j) WM1346 cells generated in (**i**) were subjected to clonogenic survival assays in RPMI-1640 media supplemented with 10% FBS for 14 days. Crystal violet was used to stain the formed colonies and representative pictures were shown from three independent experiments.

(k) WM1346 cells generated in **Fig. 3d** were subjected to cell proliferation assays in RPMI-1640 media supplemented with 10% FBS for 4 days. Cell viability was determined at the indicated time points. The viability was calculated as mean ± SD (n=3) from three independent experiments.

(l-m) The colony numbers were counted from **Fig. 3i** (**l**) and **Fig. 3j** (**m**) were calculated as mean ± SD (n=3), * $P < 0.05$; Student's *t* test.

(n) M245 and IPC-298 cells generated in (**d**,**e**) were subjected to clonogenic survival assays in RPMI-1640 media supplemented with 10% FBS for 14 days. Crystal violet was used to stain the formed colonies and representative pictures were shown from three independent experiments.

(o-p) WM1346 cells generated in **Fig. 3d** were subjected to 3D spheroid formation experiment in RPMI-1640 media supplemented with 10% FBS for 14 days (**o**). The viability of 3D spheroids formed was calculated as mean ± SD (n=3) from three independent experiments (**p**). Scale bar, 500 µm.

(q) WM1346 cells generated in **Fig. 3k** were subjected to 3D spheroid formation experiment in RPMI-1640 media supplemented with 10% FBS for 14 days. Scale bar, 500 µm.

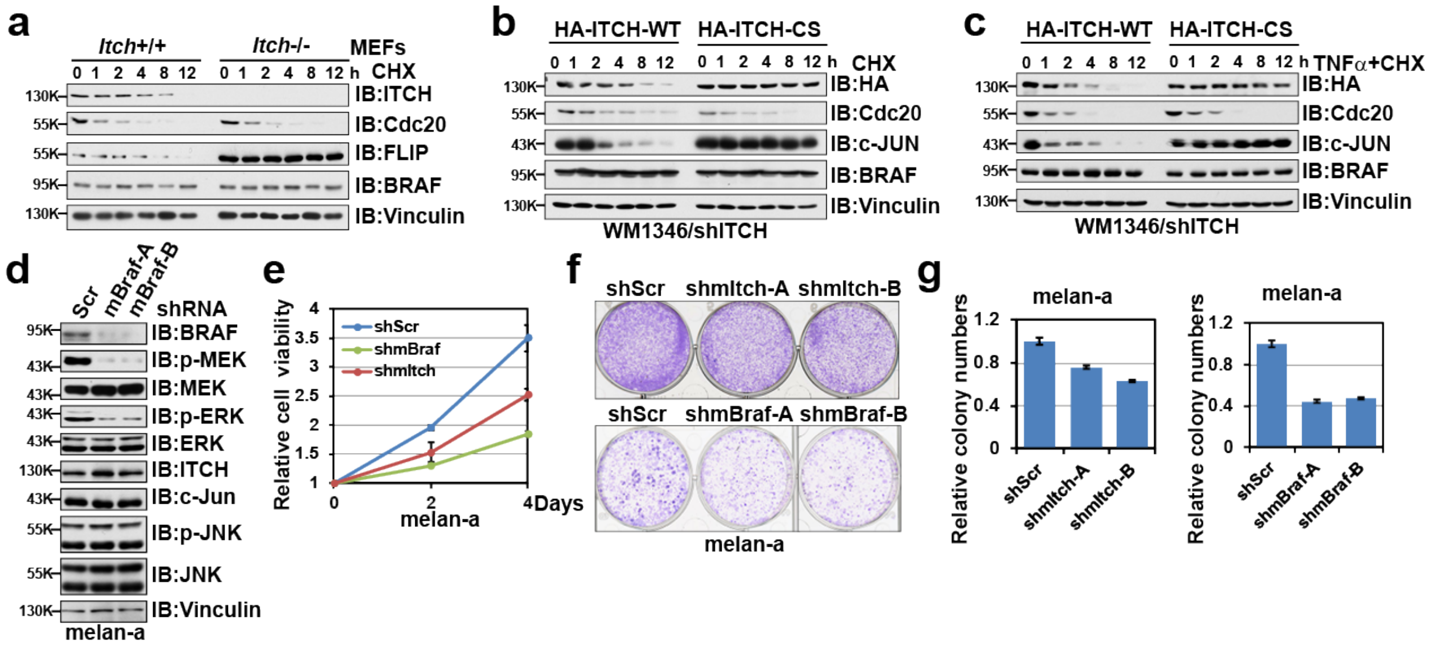

Supplementary Figure 5. Melanocytes are more Tolerate to ITCH or BRAF Deficiency

(a) Half-lives of BRAF and ITCH were determined in *Itch*$^{+/+}$ and *Itch*$^{-/-}$ MEFs. 20 μg·ml^{-1} cycloheximide (CHX) was added to cells for the indicated time periods before harvest for immunoblot (IB) analysis.

(b) Half-lives of BRAF, c-Jun and HA-ITCH were determined in WT-ITCH and C832S(CS)-ITCH-expressing shITCH-WM1346 cells. 20 μg·ml^{-1} cycloheximide (CHX) was added to cells for the indicated time periods before harvest for IB analysis.

(c) Half-lives of BRAF, c-Jun and HA-ITCH were determined in the cells described in (**b**) without or with TNF[illegible] treatment. 20 μg·ml^{-1} cycloheximide (CHX) was added to cells for the indicated time periods before harvest for IB analysis.

(d) IB analysis of WCL derived from melan-a cells infected with the indicated sh*Braf* lentiviral constructs, shScr was used as the negative control.

(e) melan-a cells generated in (**d**) and **Supplementary Fig. 4c** were subjected to cell proliferation assays in RPMI-1640 media supplemented with 10% FBS for 4 days. Cell viability was determined at the indicated time points. The viability was calculated as mean ± SD (n=3) from three independent experiments.

(f-g) melan-a cells generated in (**d**) and **Supplementary Fig. 4c** were subjected to clonogenic survival assays. Crystal violet was used to stain the formed colonies (**f**) and the colony numbers were counted from three independent experiments. The colony numbers were calculated as mean ± SD (n=3) (**g**).

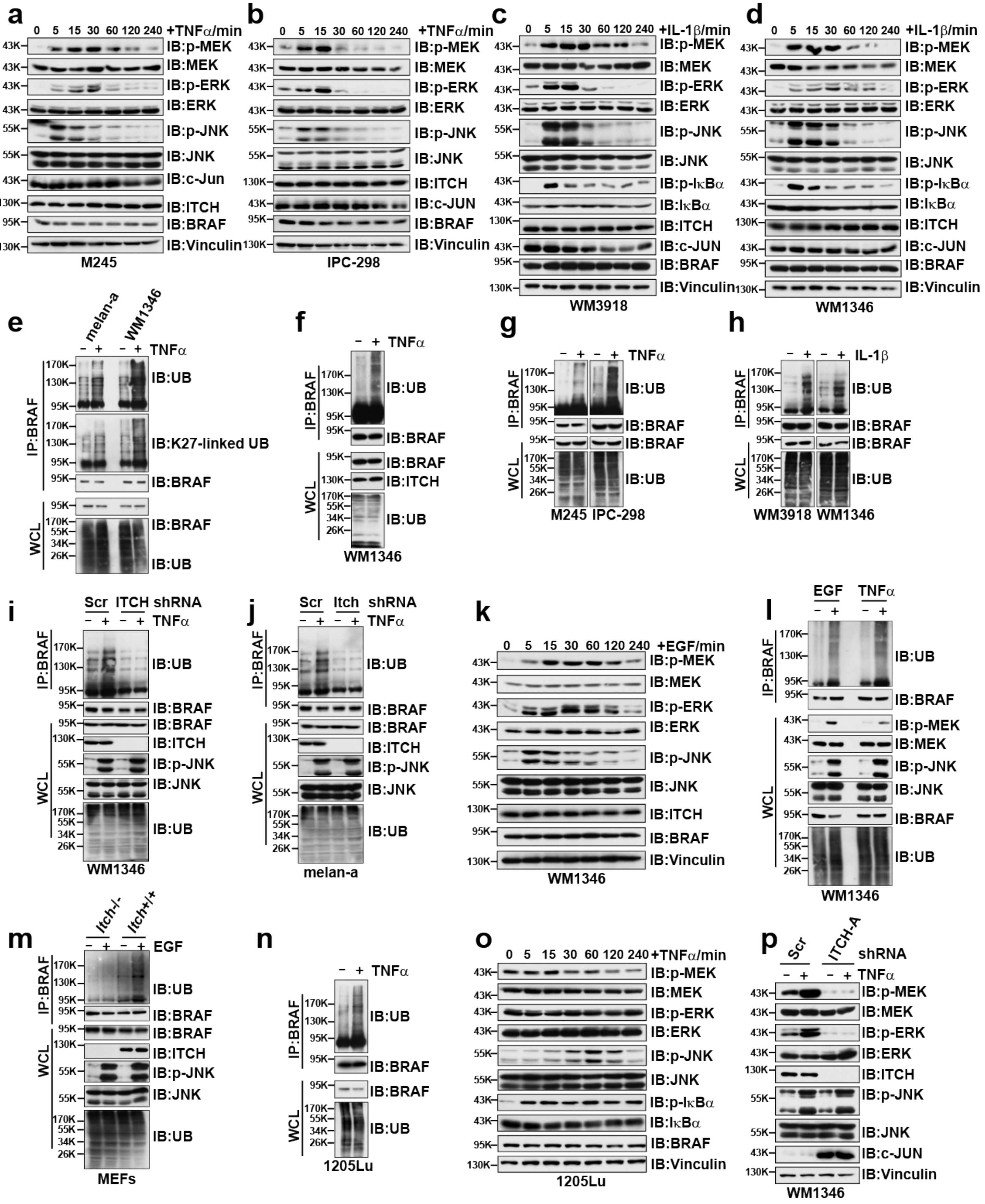
a
0 5 15 30 60 120 240 +TNFα/min
IB:p-MEK
IB:MEK
IB:p-ERK
IB:ERK
IB:p-JNK
IB:JNK
IB:c-Jun
IB:ITCH
IB:BRAF
IB:Vinculin
M245
b
0 5 15 30 60 120 240 +TNFα/min
IB:p-MEK
IB:MEK
IB:p-ERK
IB:ERK
IB:p-JNK
IB:JNK
IB:ITCH
IB:c-JUN
IB:BRAF
IB:Vinculin
IPC-298
c
0 5 15 30 60 120 240 +IL-1β/min
IB:p-MEK
IB:MEK
IB:p-ERK
IB:ERK
IB:p-JNK
IB:JNK
IB:p-IκBα
IB:IκBα
IB:ITCH
IB:c-JUN
IB:BRAF
IB:Vinculin
WM3918
d
0 5 15 30 60 120 240 +IL-1β/min
IB:p-MEK
IB:MEK
IB:p-ERK
IB:ERK
IB:p-JNK
IB:JNK
IB:p-IκBα
IB:IκBα
IB:ITCH
IB:c-JUN
IB:BRAF
IB:Vinculin
WM1346
e
melan-a WM1346
− + − + TNFα
IP:BRAF
IB:UB
IB:K27-linked UB
IB:BRAF
WCL
IB:BRAF
IB:UB
f
− + TNFα
IP:BRAF
IB:UB
IB:BRAF
WCL
IB:BRAF
IB:ITCH
IB:UB
WM1346
g
− + − + TNFα
IP:BRAF
IB:UB
IB:BRAF
WCL
IB:BRAF
IB:UB
M245 IPC-298
h
− + − + IL-1β
IP:BRAF
IB:UB
IB:BRAF
WCL
IB:BRAF
IB:UB
WM3918 WM1346
i
Scr ITCH shRNA
− + − + TNFα
IP:BRAF
IB:UB
IB:BRAF
WCL
IB:BRAF
IB:ITCH
IB:p-JNK
IB:JNK
IB:UB
WM1346
j
Scr Itch shRNA
− + − + TNFα
IP:BRAF
IB:UB
IB:BRAF
WCL
IB:BRAF
IB:ITCH
IB:p-JNK
IB:JNK
IB:UB
melan-a
k
0 5 15 30 60 120 240 +EGF/min
IB:p-MEK
IB:MEK
IB:p-ERK
IB:ERK
IB:p-JNK
IB:JNK
IB:ITCH
IB:BRAF
IB:Vinculin
WM1346
l
EGF TNFα
− + − +
IP:BRAF
IB:UB
IB:BRAF
WCL
IB:p-MEK
IB:MEK
IB:p-JNK
IB:JNK
IB:BRAF
IB:UB
WM1346
m
Itch-/- Itch+/+
− + − + EGF
IP:BRAF
IB:UB
IB:BRAF
WCL
IB:BRAF
IB:ITCH
IB:p-JNK
IB:JNK
IB:UB
MEFs
n
− + TNFα
IP:BRAF
IB:UB
IB:BRAF
WCL
IB:BRAF
IB:UB
1205Lu
o
0 5 15 30 60 120 240 +TNFα/min
IB:p-MEK
IB:MEK
IB:p-ERK
IB:ERK
IB:p-JNK
IB:JNK
IB:p-IκBα
IB:IκBα
IB:BRAF
IB:Vinculin
1205Lu
p
Scr ITCH-A shRNA
− + − + TNFα
IB:p-MEK
IB:MEK
IB:p-ERK
IB:ERK
IB:ITCH
IB:p-JNK
IB:JNK
IB:c-JUN
IB:Vinculin
WM1346

Supplementary Figure 6. Proinflammatory Cytokines Stimulates BRAF Ubiquitination and Subsequent Activation

(a-b) Immunoblot (IB) analysis of whole cell lysates (WCL) derived from M245 (**a**) and IPC-298 (**b**) cells treated with 50 $ng{\cdot}ml^{-1}$ TNFα for the indicated time period.

(c-d) IB analysis of WCL derived from WM3918 (**c**) and WM1346 (**d**) cells treated with 20 $ng{\cdot}ml^{-1}$ IL-1β for the indicated time period.

(e-g) Endogenous BRAF ubiquitination was increased upon TNFα treatment. IB analysis of WCL and anti-BRAF immunoprecipitates (IP) derived from melan-a (**e**), WM1346 (**e-f**), M245 and IPC-298 (**g**) cells without treatment or treated with 50 $ng{\cdot}ml^{-1}$ TNFα for 20 min.

(h) Endogenous BRAF ubiquitination was increased upon IL-1β treatment. IB analysis of WCL and anti-BRAF immunoprecipitates (IP) derived from WM1346 and WM3918 cells without treatment or treated with 20 $ng{\cdot}ml^{-1}$ IL-1β for 20 min.

(i-j) TNFα-induced endogenous BRAF ubiquitination was abrogated upon ITCH knockdown. IB analysis of WCL and anti-BRAF IP derived from WM1346 (**i**) and melan-a (**j**) transiently expressing shScr or the sh*ITCH* lentiviral construct. The cells were treated with 50 $ng{\cdot}ml^{-1}$ TNFα as indicated for 20 min before harvest.

(k) IB analysis of WCL derived from WM1346 cells treated with 10 $ng{\cdot}ml^{-1}$ EGF for the indicated time period.

(l) Endogenous BRAF ubiquitination was increased upon EGF or TNFα treatment. IB analysis of WCL and anti-BRAF IP derived from WM1346 cells without treatment or treated with 10 $ng{\cdot}ml^{-1}$ EGF or 50 $ng{\cdot}ml^{-1}$ TNFα for 20 min.

(m) Endogenous BRAF ubiquitination was increased upon EGF in WT but not $Itch^{-/-}$ MEFs. IB analysis of WCL and anti-BRAF IP derived from WT and $Itch^{-/-}$ MEFs cells without treatment or treated with 10 $ng{\cdot}ml^{-1}$ EGF for 20 min.

(n) Endogenous BRAF ubiquitination was increased upon TNFα treatment. IB analysis of WCL and anti-BRAF IP derived from 1205Lu cells without treatment or treated with 50 $ng{\cdot}ml^{-1}$ TNFα for 20 min.

(o) IB analysis of WCL derived from 1205Lu cells treated with 50 $ng{\cdot}ml^{-1}$ TNFα for the indicated time period.

(p) IB analysis of WCL derived from shScr- and sh*ITCH*-WM1346 cells without or with 50 $ng{\cdot}ml^{-1}$ TNFα treatment for 20 min.

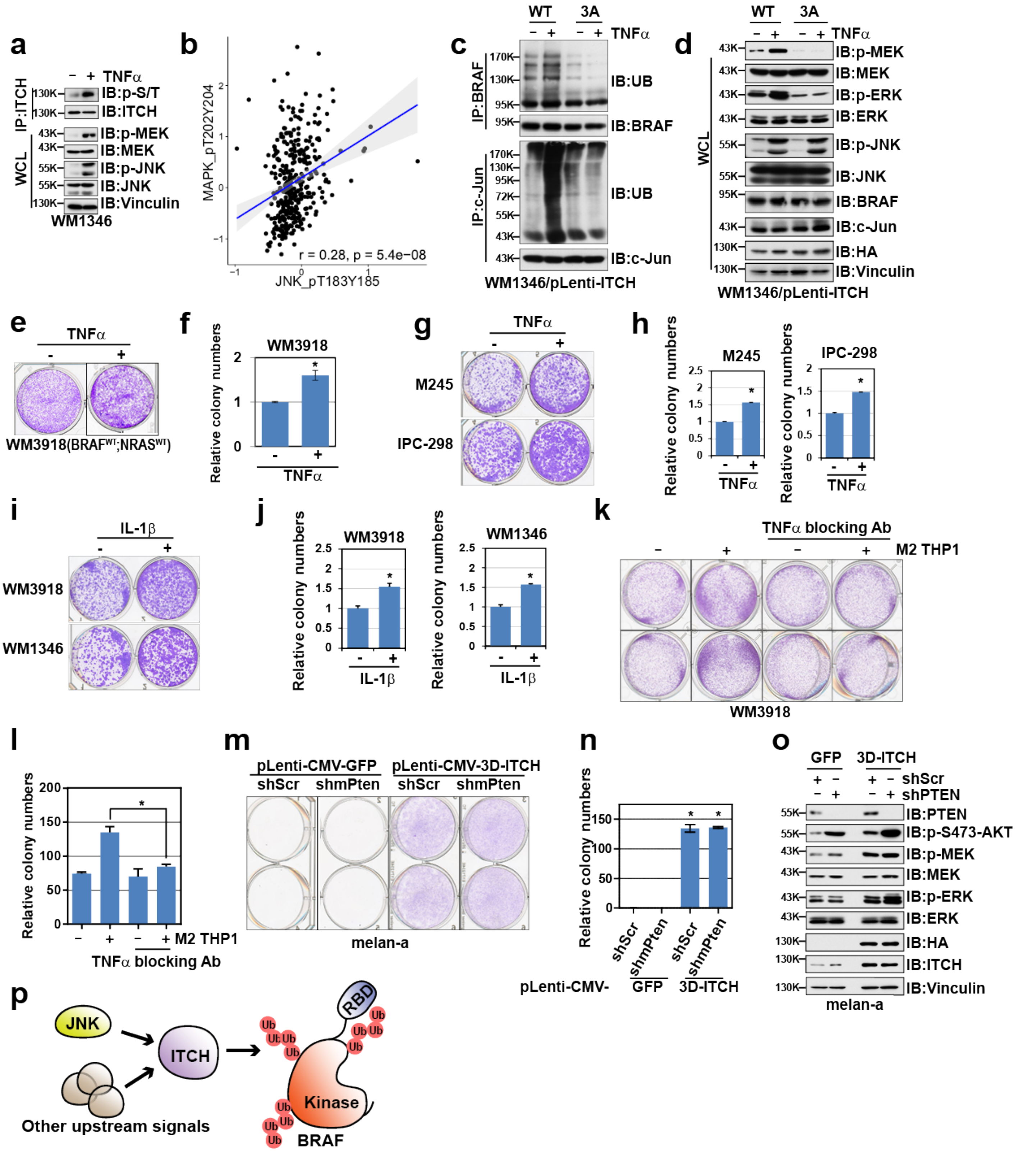

a
b
c
d
e
f
g
h
i
j
k
l
m
n
o
p
IP:ITCH
WCL
TNFα
IB:p-S/T
IB:ITCH
IB:p-MEK
IB:MEK
IB:p-JNK
IB:JNK
IB:Vinculin
WM1346
MAPK_pT202Y204
JNK_pT183Y185
r = 0.28, p = 5.4e−08
WT
3A
IP:BRAF
IP:c-Jun
IB:UB
IB:BRAF
IB:c-Jun
WM1346/pLenti-ITCH
IB:p-ERK
IB:ERK
IB:HA
WM3918(BRAFWT;NRASWT)
Relative colony numbers
WM3918
M245
IPC-298
IL-1β
TNFα blocking Ab
M2 THP1
pLenti-CMV-GFP
pLenti-CMV-3D-ITCH
shScr
shmPten
melan-a
pLenti-CMV-
GFP
3D-ITCH
shPTEN
IB:PTEN
IB:p-S473-AKT
JNK
ITCH
Other upstream signals
RBD
Kinase
BRAF
Ub

Supplementary Figure 7. Proinflammatory Cytokines Stimulates Proliferation of Melanoma Cells

(a) Immunoblot (IB) analysis of whole cell lysates (WCL) and anti-ITCH immunoprecipitates (IP) derived derived from WM1346 cells treated with 50 ng·ml^{-1} TNFα for 20 min.

(b) Scatter plot showing the correlation between pT183/pY185-JNK and pT202/pY204-ERK from the TCGA cutaneous melanoma (TCGA-SKCM) RPPA (reverse phase protein array) dataset containing 354 tumors. The Pearson correlation coefficient *r* and the *p* value were calculated using the ggscatter function from the ggpubr package.

(c-d) Endogenous BRAF ubiquitination was increased upon TNFα treatment in WT- but not in activated 3A-ITCH-expressing WM1346 cells. IB analysis of anti-BRAF and anti-c-Jun IP (**c**) as well as WCL (**d**) and derived from WT- and 3A-ITCH-WM1346 cells without treatment or treated with 50 ng·ml^{-1} TNFα for 20 min.

(e-f) WM3918 cells without or with 50 ng·ml^{-1} TNFα treatment were subjected to clonogenic survival assays. Crystal violet was used to stain the formed colonies (**e**) and the colony numbers were counted from three independent experiments. The colony numbers were calculated as mean ± SD (n=3), * $P < 0.05$; Student's *t* test (**f**).

(g-h) M245 and IPC-298 cells without or with 50 ng·ml^{-1} TNFα treatment were subjected to clonogenic survival assays. Crystal violet was used to stain the formed colonies (**g**) and the colony numbers were counted from three independent experiments. The colony numbers were calculated as mean ± SD (n=3), * $P < 0.05$; Student's *t* test (**h**).

(i-j) WM3918 and WM1346 cells without or with 20 ng·ml^{-1} IL-1β treatment were subjected to clonogenic survival assays. Crystal violet was used to stain the formed colonies (**i**) and the colony numbers were counted from three independent experiments. The colony numbers were calculated as mean ± SD (n=3), * $P < 0.05$; Student's *t* test (**j**).

(k-l) WM3918 cells were subjected to clonogenic survival assays without or with M2 differentiated THP1, TNFα blocking antibody (5ng·ml^{-1}) was added as indicated. Crystal violet was used to stain the formed colonies (**k**) and the colony numbers were counted from three independent experiments. The colony numbers were calculated as mean ± SD (n=3), * $P < 0.05$; Student's *t* test (**l**).

(m-n) melan-a cells generated in **Fig. 4m** were further transduced with shScr or sh*Pten* lentiviral constructs followed by clonogenic survival assays without TPA for 16 days. Crystal violet was used to stain the formed colonies (**m**) and the colony numbers were counted from three independent experiments. The colony numbers were calculated as mean ± SD (n=3), * $P < 0.05$; Student's *t* test (**n**).

(o) melan-a cells generated in **Fig. 4m** were further transduced with shScr or sh*Pten* lentiviral constructs before harvest for IB analysis.

(p) A schematic illustration of the model that upon cytokine stimulation, activated JNK restores ITCH E3 ligase activity to promote BRAF ubiquitination and subsequent elevation of MEK/ERK signals.

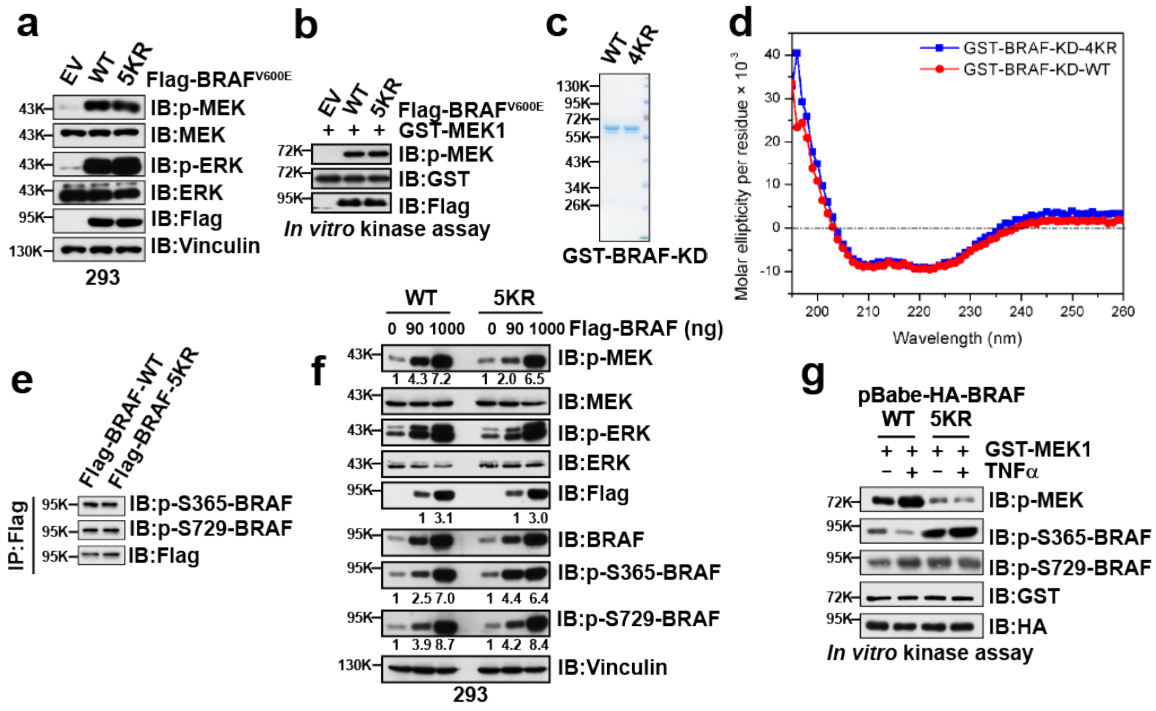

Supplementary Figure 8. Ubiquitination-deficient BRAF Mutant Displays Elevated S365 Phosphorylation in Cells

(a) Immunoblot (IB) analysis of whole cell lysates (WCL) derived from HEK293 cells transfected with the indicated Flag-tagged $BRAF^{V600E}$ or $BRAF^{V600E+5KR}$ mutants.

(b) *In vitro* kinase assays showing that immuno-purified Flag- $BRAF^{V600E}$ and $BRAF^{V600E+5KR}$ proteins displayed similar activity to promote the phosphorylation of GST-MEK1.

(c) Coomassie blue staining of purified WT and 4KR-BRAF kinase domain (KD) that were used for circular dichroism spectroscopy experiments shown in (**d**).

(d) Circular dichroism spectroscopy experiments showing that WT and 4KR-BRAF kinase domain (KD) exhibited similar secondary structure.

(e) IB analysis of immuno-purified Flag-BRAF proteins used in *in vitro* kinase assays from **Fig. 5b**.

(f) IB analysis of WCL derived from HEK293 cells transfected with the indicated amount of Flag-WT-BRAF or Flag-5KR-BRAF constructs. The densitometry was determined using ImageJ.

(g) *In vitro* kinase assays using immuno-purified HA-WT-BRAF and HA-5KR-BRAF from stable WM1346 cell lines as described in **Fig. 7a.**

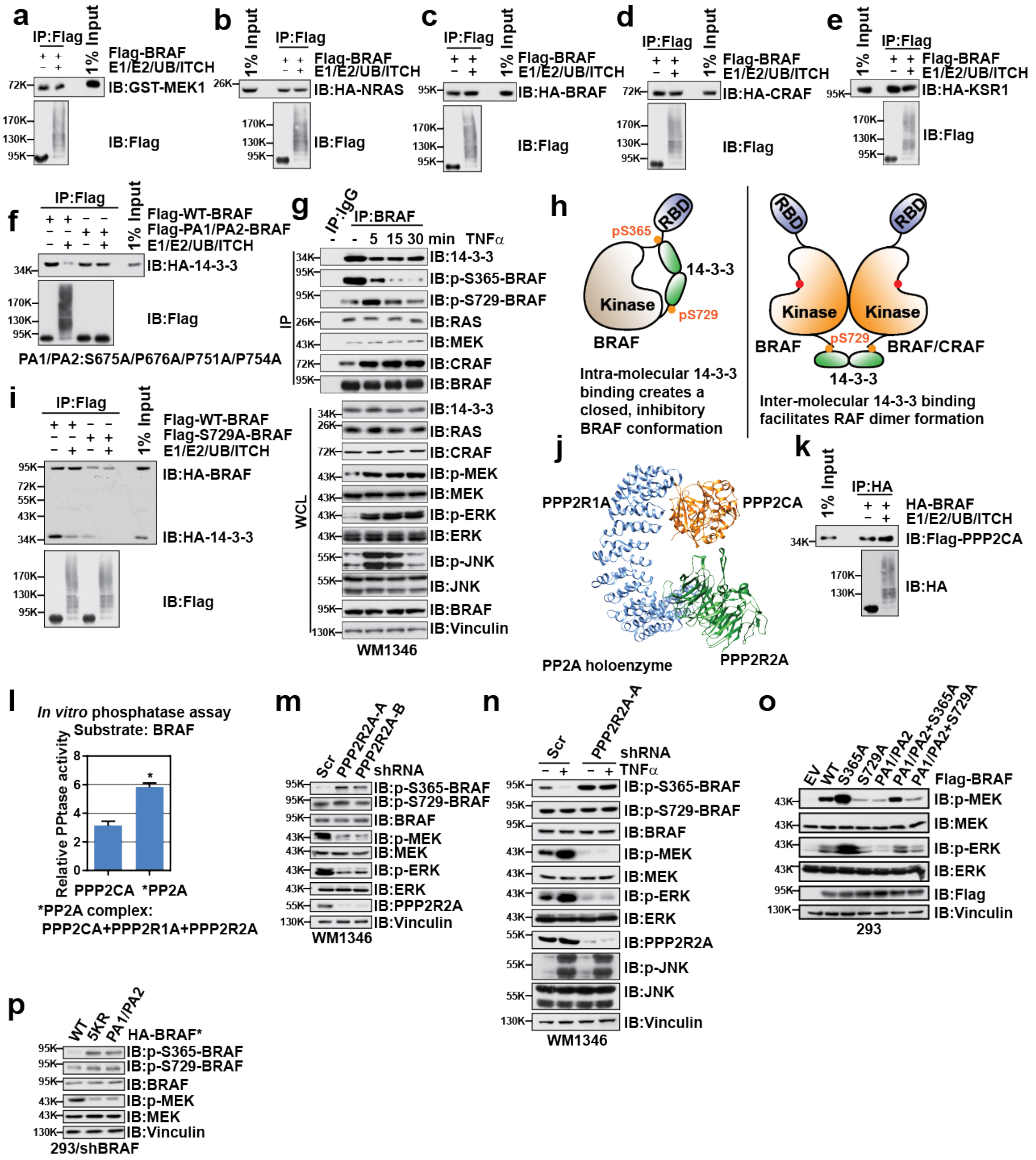
a
IP:Flag
1% Input
Flag-BRAF
E1/E2/UB/ITCH
IB:GST-MEK1
IB:Flag
b
IB:HA-NRAS
c
IB:HA-BRAF
d
IB:HA-CRAF
e
IB:HA-KSR1
f
Flag-WT-BRAF
Flag-PA1/PA2-BRAF
IB:HA-14-3-3
PA1/PA2:S675A/P676A/P751A/P754A
g
IP:IgG
IP:BRAF
5 15 30 min TNFα
IB:14-3-3
IB:p-S365-BRAF
IB:p-S729-BRAF
IB:RAS
IB:MEK
IB:CRAF
IB:BRAF
IP
WCL
IB:p-MEK
IB:p-ERK
IB:ERK
IB:p-JNK
IB:JNK
IB:Vinculin
WM1346
h
pS365
RBD
14-3-3
Kinase
pS729
BRAF
Intra-molecular 14-3-3 binding creates a closed, inhibitory BRAF conformation
BRAF/CRAF
Inter-molecular 14-3-3 binding facilitates RAF dimer formation
i
Flag-S729A-BRAF
j
PPP2R1A
PPP2CA
PPP2R2A
PP2A holoenzyme
k
IP:HA
HA-BRAF
IB:Flag-PPP2CA
IB:HA
l
In vitro phosphatase assay
Substrate: BRAF
Relative PPtase activity
PPP2CA
*PP2A
*PP2A complex: PPP2CA+PPP2R1A+PPP2R2A
m
Scr
PPP2R2A-A
PPP2R2A-B
shRNA
IB:PPP2R2A
n
o
EV
WT
S365A
S729A
PA1/PA2
PA1/PA2+S365A
PA1/PA2+S729A
Flag-BRAF
IB:Flag
293
p
5KR
HA-BRAF*
293/shBRAF

Supplementary Figure 9. BRAF Ubiquitination Attenuates the Interaction with 14-3-3

(a-e) Ubiquitination of BRAF did not affect its binding with various interacting proteins *in vitro*. *In vitro* ubiquitination assays using immuno-purified Flag-BRAF proteins were performed followed by incubation of Flag-BRAF with various proteins including MEK1 (**a**), NRAS (**b**), BRAF (**c**), CRAF(**d**), and KSR1 (**e**). The anti-Flag immunoprecipitates (IP) were subjected to SDS-PAGE and immunoblot (IB) analysis.

(f) ITCH-binding deficient PA1/PA2-BRAF displayed stronger binding to 14-3-3 compared to WT-BRAF. *In vitro* ubiquitination assays using the indicated immuno-purified Flag-BRAF proteins were performed followed by incubation of Flag-BRAF with 14-3-3 proteins. The anti-Flag IP were subjected to SDS-PAGE and IB analysis.

(g) Binding between endogenous BRAF and 14-3-3 was reduced upon TNFα treatment. IB analysis of whole cell lysates (WCL) and anti-BRAF IP derived from WM1346 cells treated with 50 $ng \cdot ml^{-1}$ TNFα for the indicated time period.

(h) A schematic illustration of the different roles 14-3-3 plays in regulating BRAF activation and dimerization.

(i) BRAF ubiquitination failed to influence its dimerization *in vitro*. *In vitro* ubiquitination assays using the indicated immuno-purified Flag-BRAF proteins were performed followed by incubation of Flag-BRAF with BRAF and 14-3-3 proteins. The anti-Flag IP were subjected to SDS-PAGE and IB analysis.

(j) A structural illustration of the molecular architecture of the PP2A holoenzyme [2] (PDB ID: 3DW8).

(k) Ubiquitination of BRAF did not affect its binding with PPP2CA *in vitro*. *In vitro* ubiquitination assays using immuno-purified HA-BRAF proteins were performed followed by incubation of HA-BRAF with PPP2CA. The anti-HA IP were subjected to SDS-PAGE and IB analysis.

(l) *In vitro* phosphatase assay using immuno-purified PPP2CA or PP2A complex for Flag-BRAF dephosphorylation. immuno-purified Flag-BRAF was incubated with the indicated PP2A phosphatases for 60 min at 30 °C. The free phosphate was measured using the malachite green detection reagent, normalized, and calculated as mean ± SD (n=3) from three independent experiments. * $P < 0.05$; Student's *t* test.

(m) IB analysis of WCL derived from WM1346 cells infected with the indicated sh*PPP2R2A* lentiviral constructs, shScr was used as the negative control.

(n) IB analysis of WCL derived from WM1346 cells infected with shScr or sh*PPP2R2A* lentiviral constructs, the cells were treated with 50 $ng \cdot ml^{-1}$ TNFα for 20 min as indicated before harvest.

(o-p) IB analysis of WCL derived from HEK293 cells transfected with the indicated Flag-tagged WT-BRAF or BRAF mutants.

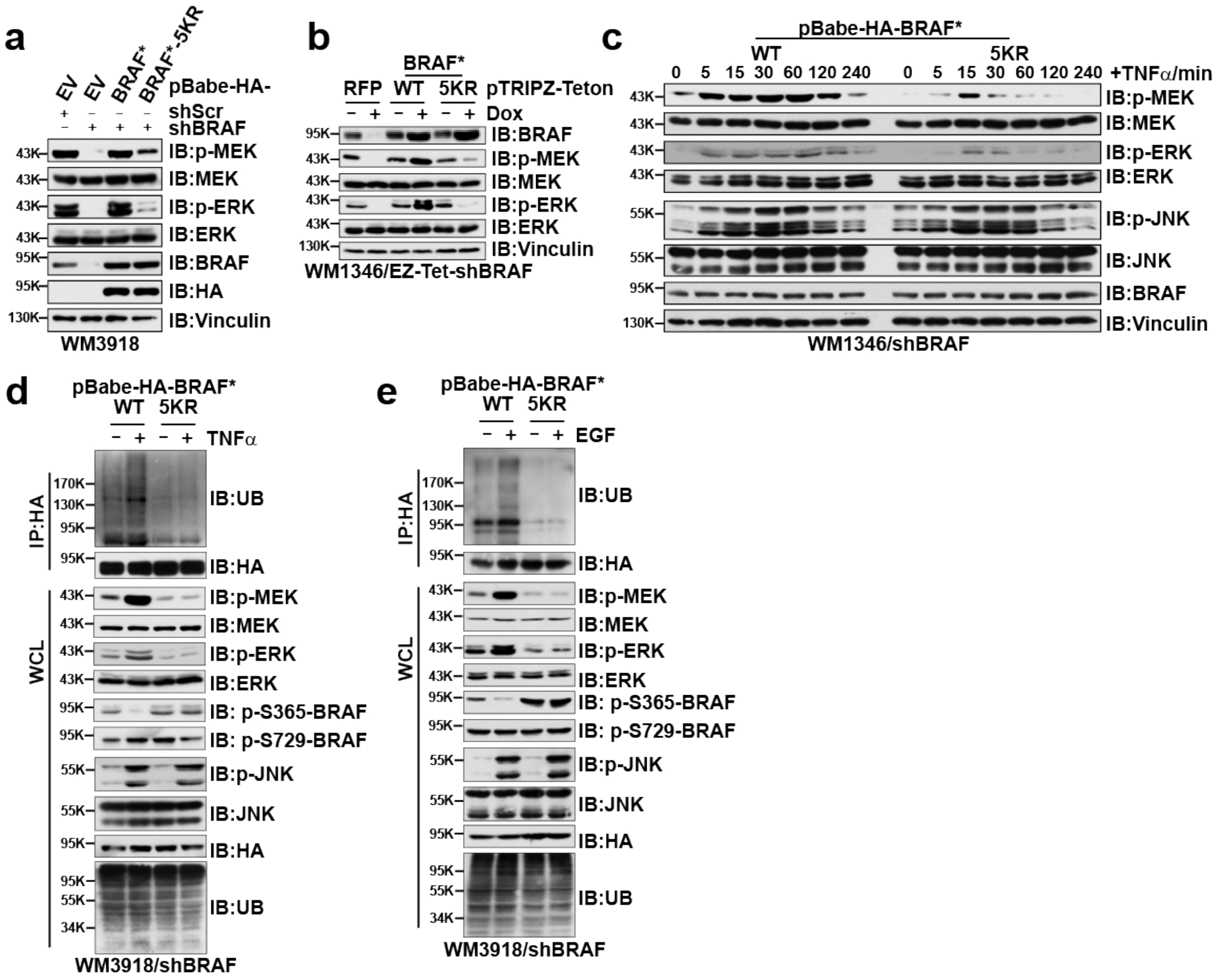

Supplementary Figure 10. Ubiquitination-deficient BRAF Fails to Activate MEK/ERK in Melanoma Cells

(a) Immunoblot (IB) analysis of whole cell lysates (WCL) derived from WM3918 cells stably expressing EV, WT-BRAF or the 5KR-BRAF mutant. The stable cell lines were further infected with the indicated lentiviral vectors to deplete endogenous *BRAF*. *The BRAF cDNA used in this Figure was mutated to confer resistant to the sh*BRAF* lentiviral vector.

(b) IB analysis of WCL derived from WM1346 cells stably expressing EZ-Tet-pLKO-sh*BRAF* and the indicated doxycycline (Dox)-inducible pTRIPZ-CMV constructs. The cells were treated with 1 $\mu g \cdot ml^{-1}$ doxycycline (Dox) as indicated for 48 h before harvest.

(c) Immunoblot (IB) analysis of whole cell lysates (WCL) derived from WM1346 cells stably expressing WT-BRAF or 5KR-BRAF treated with 50 $ng \cdot ml^{-1}$ TNFα for the indicated time period.

(d-e) IB analysis of anti-BRAF immunoprecipitates (IP) *BRAF*-depleted WM3918 cells stably expressing WT-BRAF or the 5KR-BRAF mutant. 50 $ng \cdot ml^{-1}$ TNFα (**d**) or 10 $ng \cdot ml^{-1}$ EGF (**e**) was added to stimulate BRAF ubiquitination where indicated.

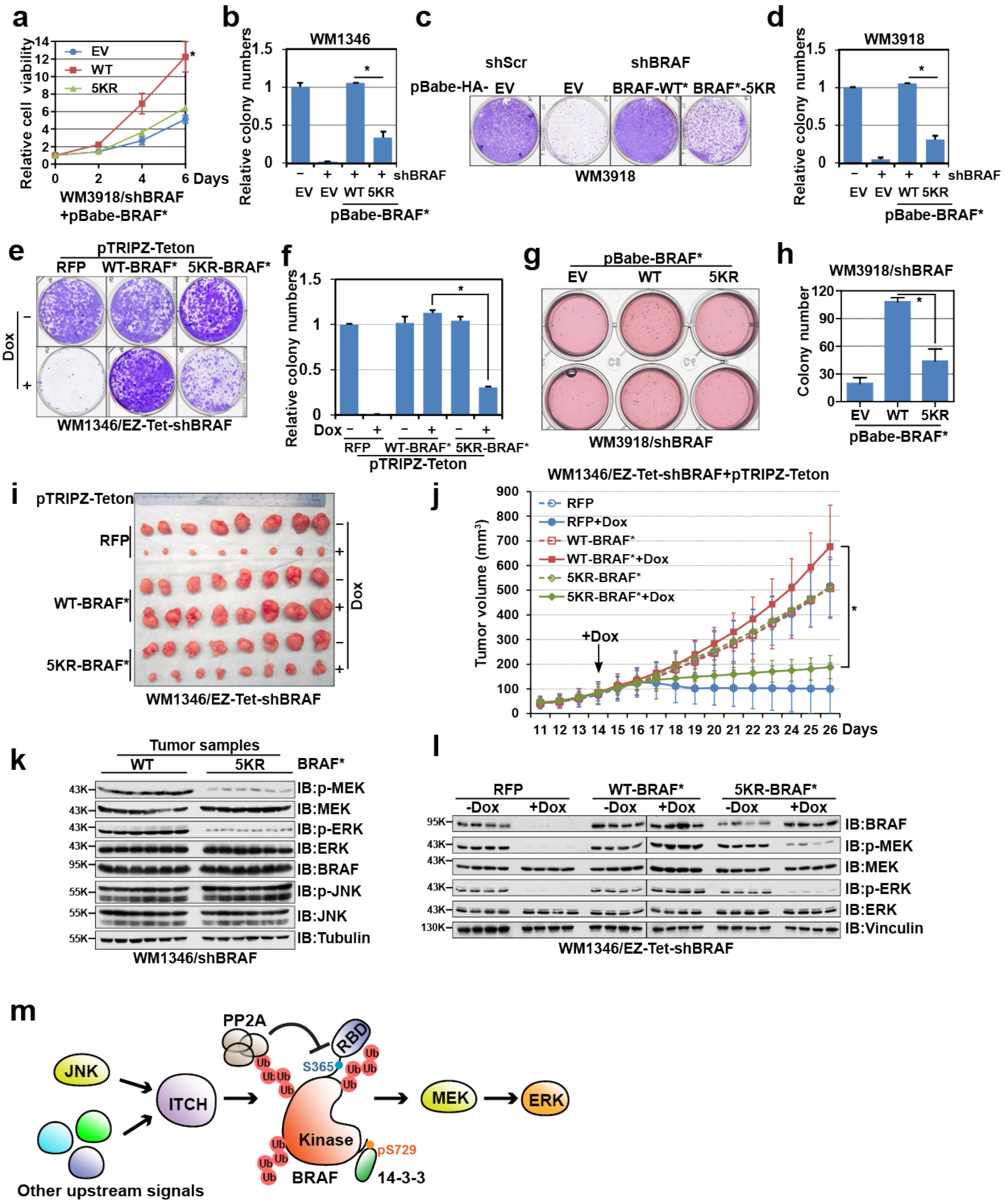
a
Relative cell viability
EV
WT
5KR
Days
WM3918/shBRAF
+pBabe-BRAF*
b
WM1346
Relative colony numbers
shBRAF
EV EV WT 5KR
pBabe-BRAF*
c
shScr
shBRAF
pBabe-HA- EV EV BRAF-WT* BRAF*-5KR
WM3918
d
WM3918
Relative colony numbers
shBRAF
EV EV WT 5KR
pBabe-BRAF*
e
pTRIPZ-Teton
RFP WT-BRAF* 5KR-BRAF*
Dox
WM1346/EZ-Tet-shBRAF
f
Relative colony numbers
Dox
RFP WT-BRAF* 5KR-BRAF*
pTRIPZ-Teton
g
pBabe-BRAF*
EV WT 5KR
WM3918/shBRAF
h
WM3918/shBRAF
Colony number
EV WT 5KR
pBabe-BRAF*
i
pTRIPZ-Teton
RFP
WT-BRAF*
5KR-BRAF*
Dox
WM1346/EZ-Tet-shBRAF
j
WM1346/EZ-Tet-shBRAF+pTRIPZ-Teton
Tumor volume (mm³)
RFP
RFP+Dox
WT-BRAF*
WT-BRAF*+Dox
5KR-BRAF*
5KR-BRAF*+Dox
+Dox
Days
k
Tumor samples
WT 5KR BRAF*
IB:p-MEK
IB:MEK
IB:p-ERK
IB:ERK
IB:BRAF
IB:p-JNK
IB:JNK
IB:Tubulin
WM1346/shBRAF
l
RFP WT-BRAF* 5KR-BRAF*
-Dox +Dox -Dox +Dox -Dox +Dox
IB:BRAF
IB:p-MEK
IB:MEK
IB:p-ERK
IB:ERK
IB:Vinculin
WM1346/EZ-Tet-shBRAF
m
PP2A
RBD
S365
JNK
ITCH
Kinase
BRAF
pS729
14-3-3
MEK
ERK
Other upstream signals

Supplementary Figure 11. Ubiquitination-deficient BRAF Exhibits Compromised Activity in Melanoma Cells

(a) WM3918 cells generated in **Supplementary Fig. 10a** were subjected to cell proliferation assays for 6 days. Cell viability was determined at the indicated time points. The viability was calculated as mean ± SD (n=3) from three independent experiments. * $P < 0.05$; Student's *t* test. *The BRAF cDNA used in this Figure was mutated to confer resistant to the sh*BRAF* lentiviral vector.

(b) The colony numbers from the experiment described in **Fig. 7d** were counted from three independent experiments. The colony numbers were calculated as mean ± SD (n=3), * $P < 0.05$; Student's *t* test**.**

(c-d) WM3918 cells generated in (**a**) were seeded (1,000 cells per well) for clonogenic survival assays. 14 days after plating, crystal violet was used to stain the formed colonies (**c**) and the colony numbers were counted from three independent experiments. The colony numbers were calculated as mean ± SD (n=3), * $P < 0.05$; Student's *t* test (**d**)**.**

(e-f) WM1346 cells generated in **Supplementary Fig. 10b** were seeded (1,000 cells per well) for clonogenic survival assays with the treatment of 1 $\mu g \cdot ml^{-1}$ doxycycline as indicated. 14 days after plating, crystal violet was used to stain the formed colonies (**e**) and the colony numbers were counted from three independent experiments. The colony numbers were calculated as mean ± SD (n=3), * $P < 0.05$; Student's *t* test (**f**)**.**

(g-h) WM1346 cells generated in **Supplementary Fig. 10a** were subjected to soft agar colony formation assays for 21 days. Formed colonies were stained with Iodonitrotetrazolium chloride (INT) (**g**). The colony numbers were counted and calculated as mean ± SD (n=3) from three independent experiments. * $P < 0.05$; Student's *t* test (**h**).

(i-j) Tumor pictures at the end point (**i**) and growth curves (**j**) for the xenograft experiments with the WM1346 cells generated in **Supplementary Fig. 10b** were inoculated subcutaneously. In each flank of six nude mice, 2×10^6 cells were injected. The visible tumors were measured at the indicated days. The arrow indicates the day doxycycline administration was started. Error bars represent ±SEM (n=6).

(k) IB analysis of WCL derived from the tumor samples generated in **Fig. 7m-n.**

(l) IB analysis of WCL derived from the tumor samples generated in (**i-j**)**.**

(m) A schematic illustration of the proposed model for the JNK/ITCH-mediated BRAF poly-ubiquitination recruits the PP2A complex to dephosphorylate p-S365-BRAF and thereby promote BRAF activation.

Supplementary Figure 12. Uncropped Original Scans

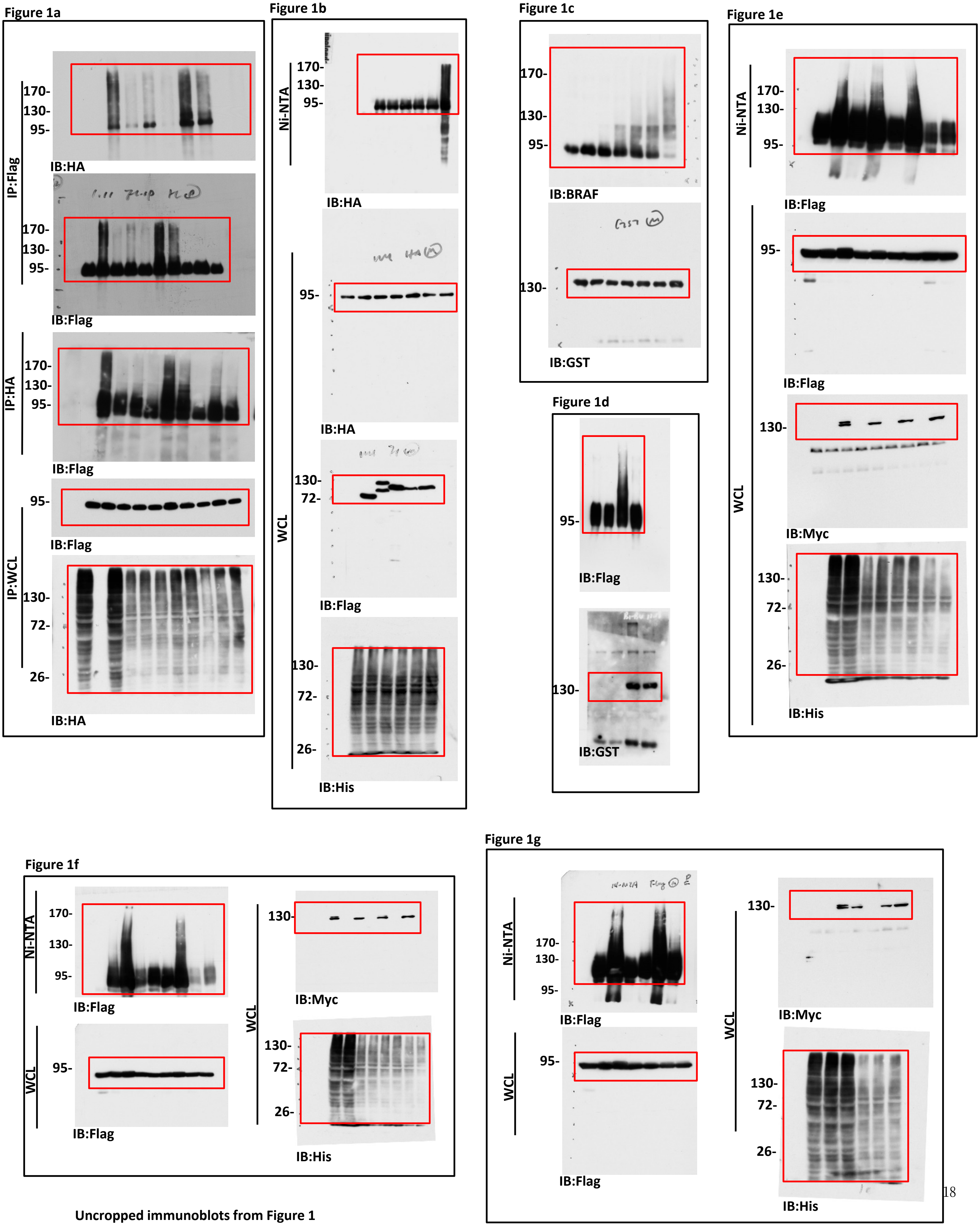

Uncropped immunoblots from Figure 1

Figure 1h

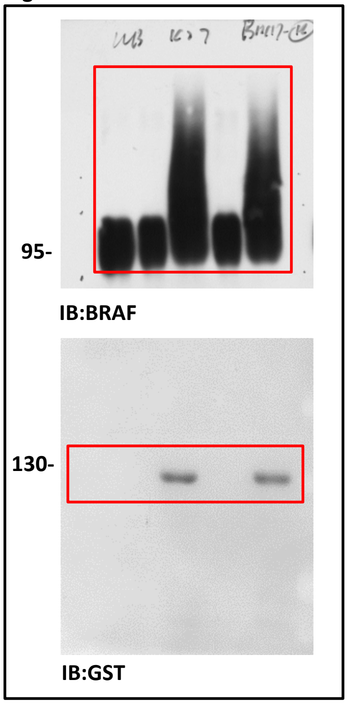

Figure 1i

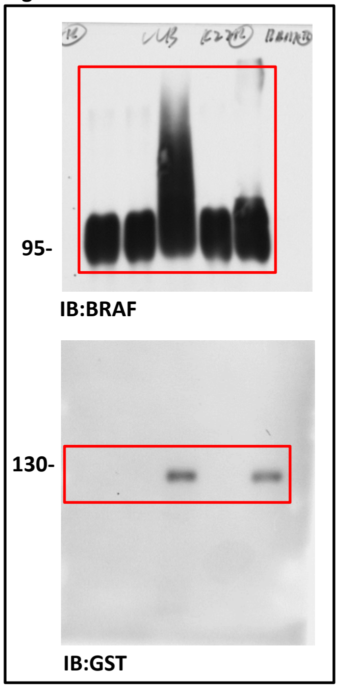

Figure 1k

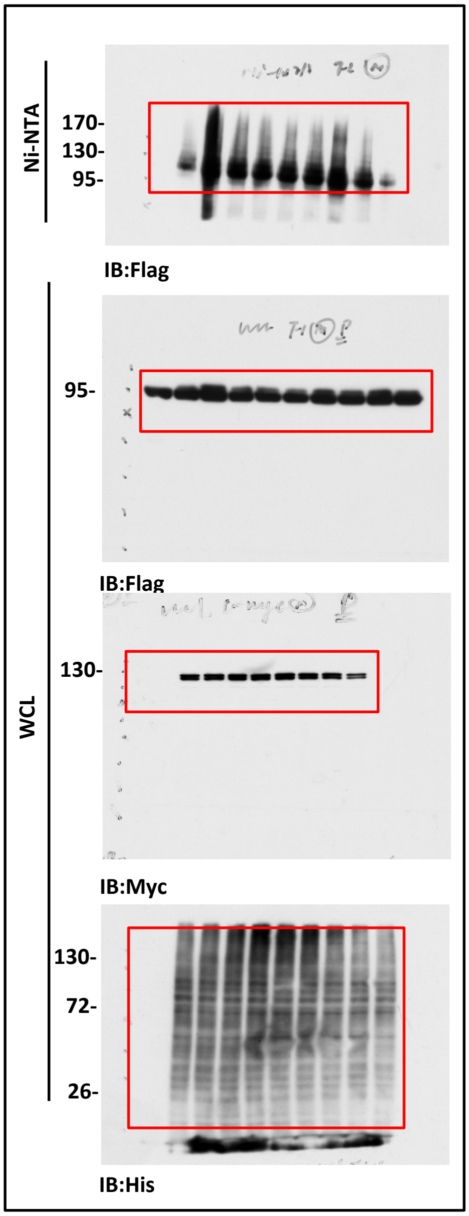

Figure 1l

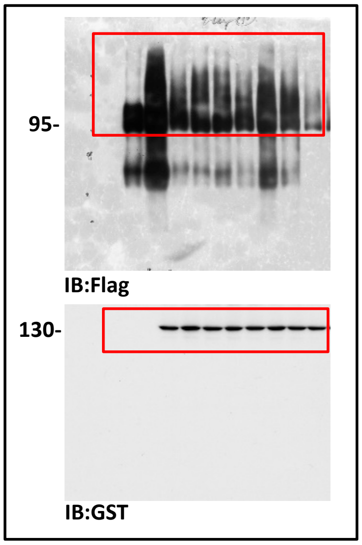

Uncropped immunoblots from Figure 1

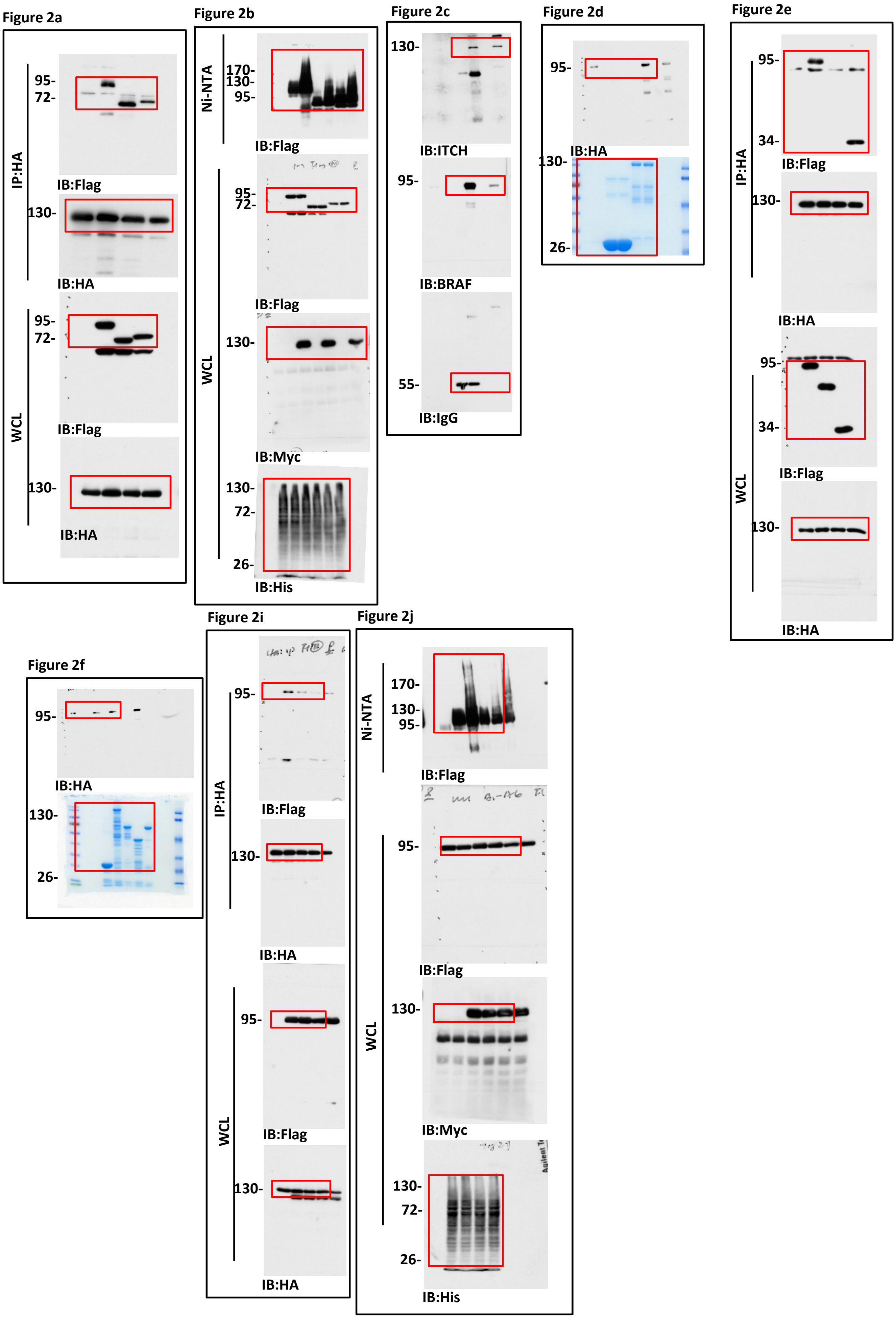

Uncropped immunoblots from Figure 2

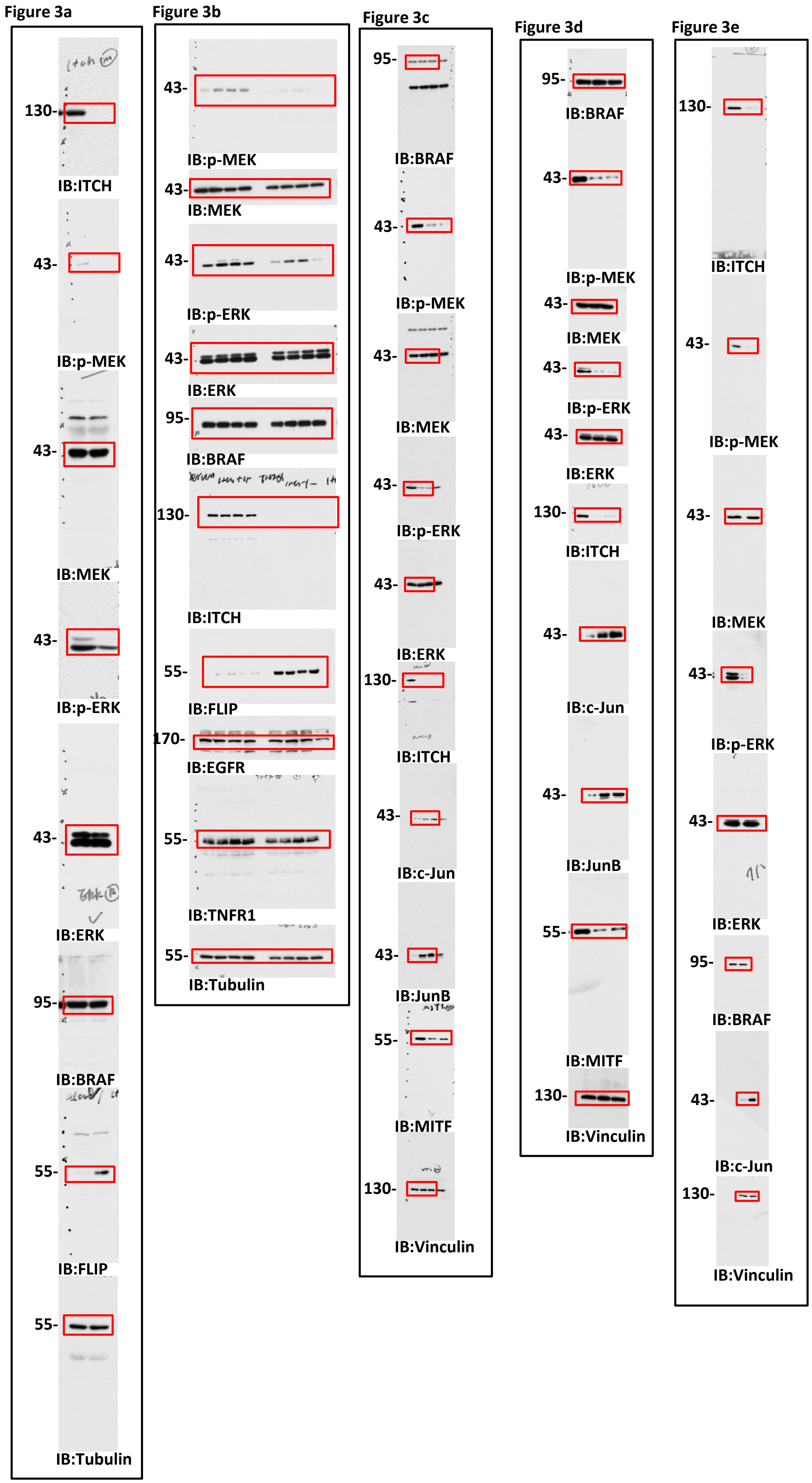

Uncropped immunoblots from Figure 3

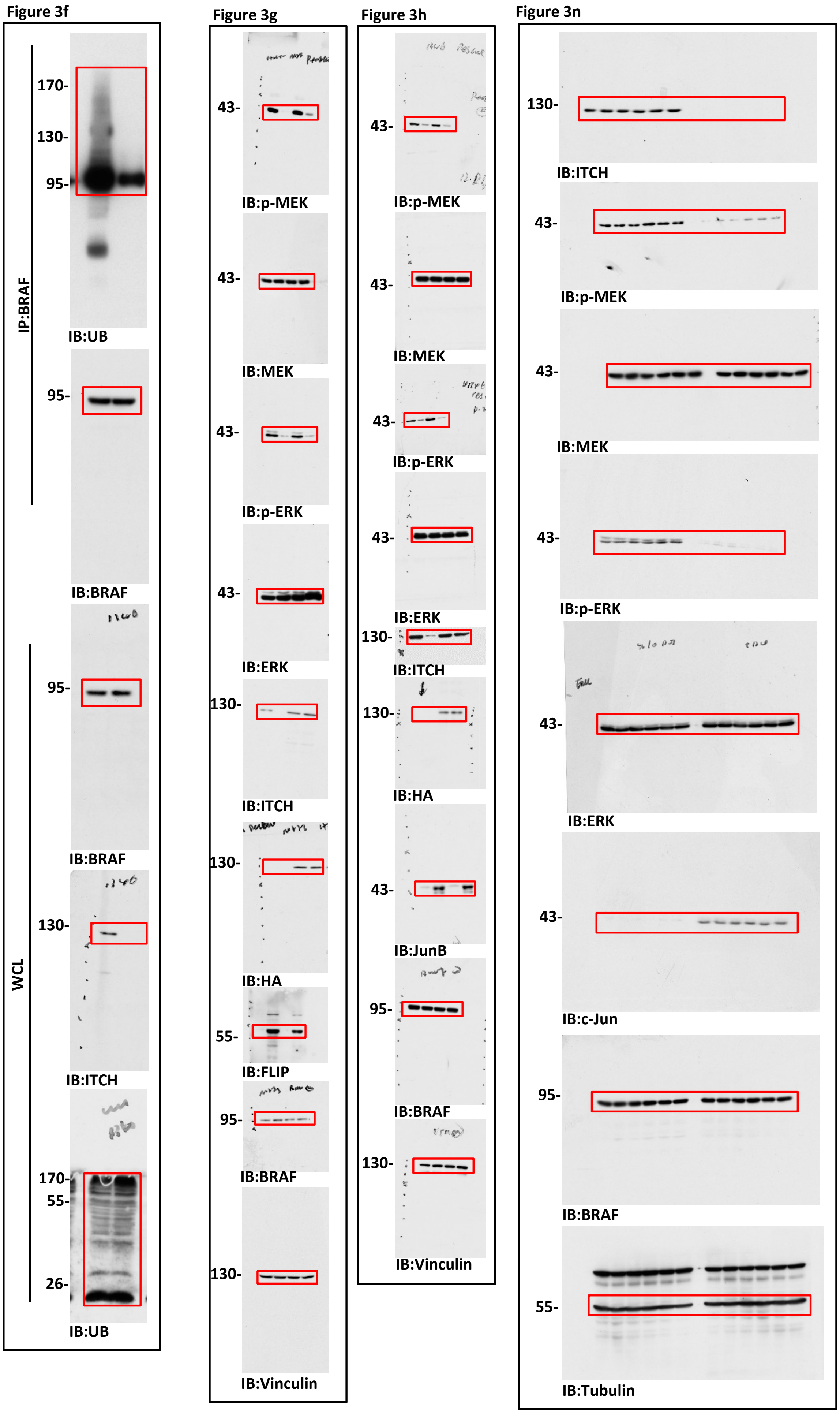

Uncropped immunoblots from Figure 3

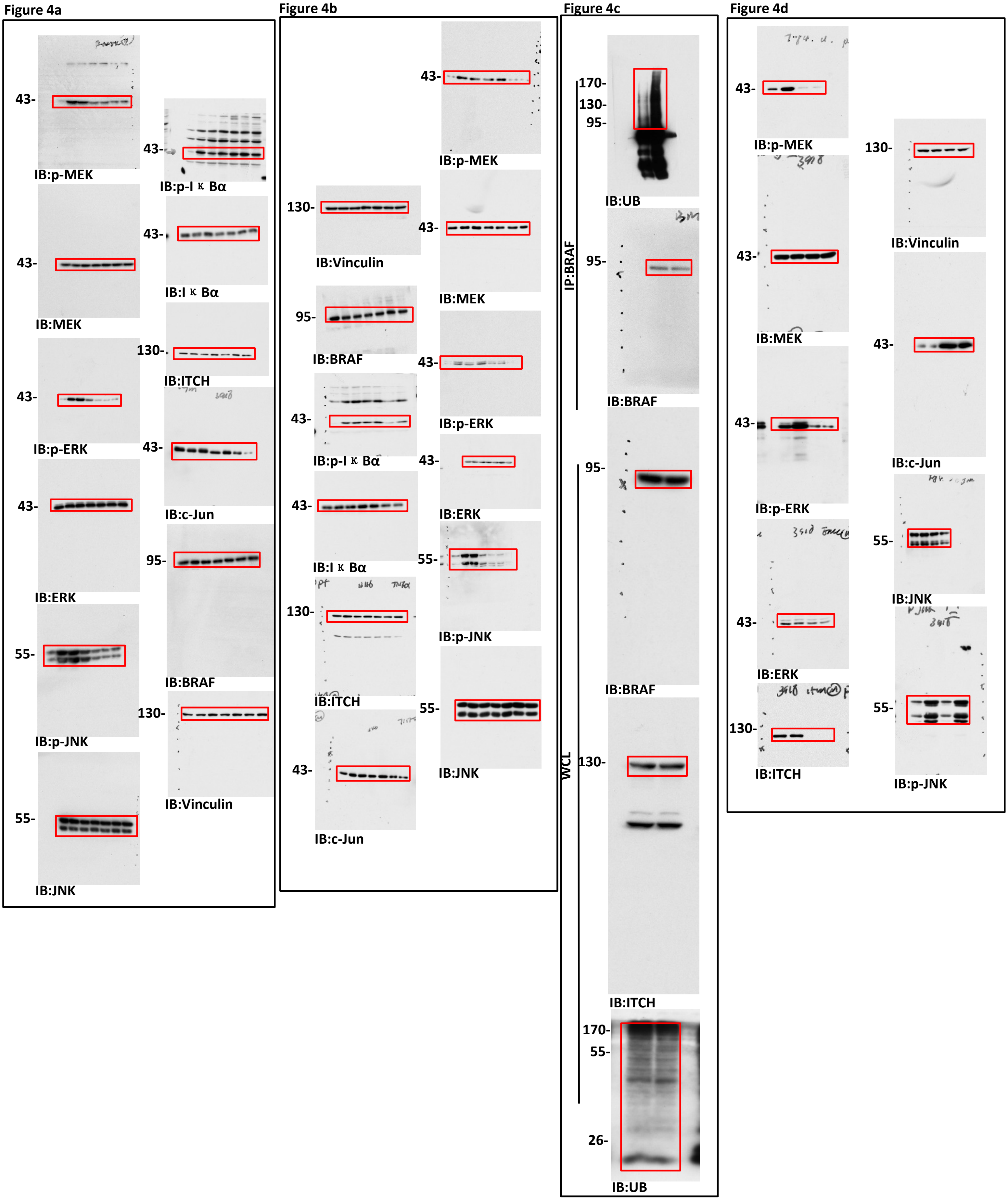

Uncropped immunoblots from Figure 4

Figure 4e

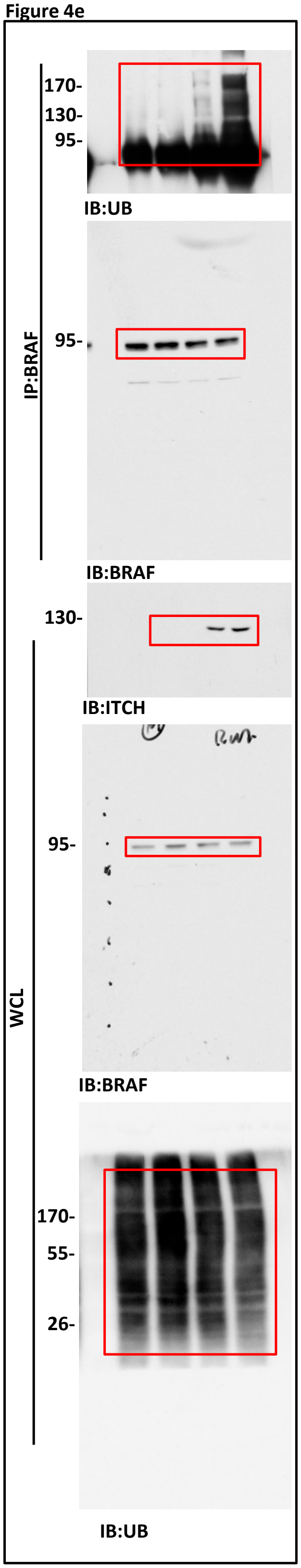

Figure 4f

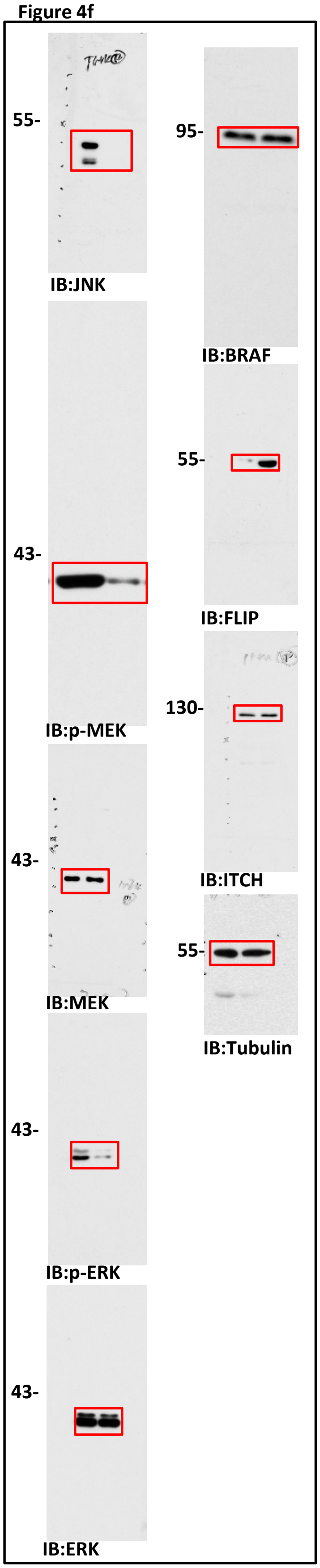

Figure 4g

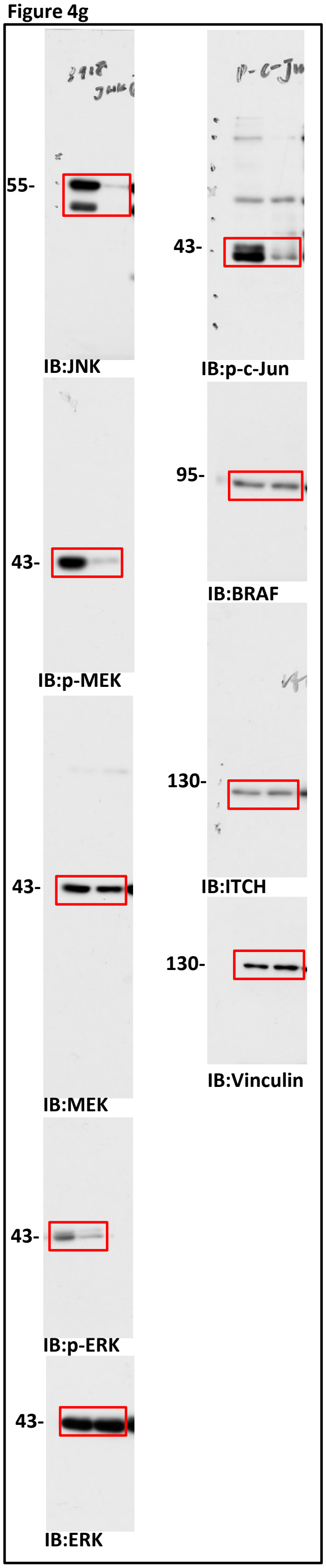

Figure 4l

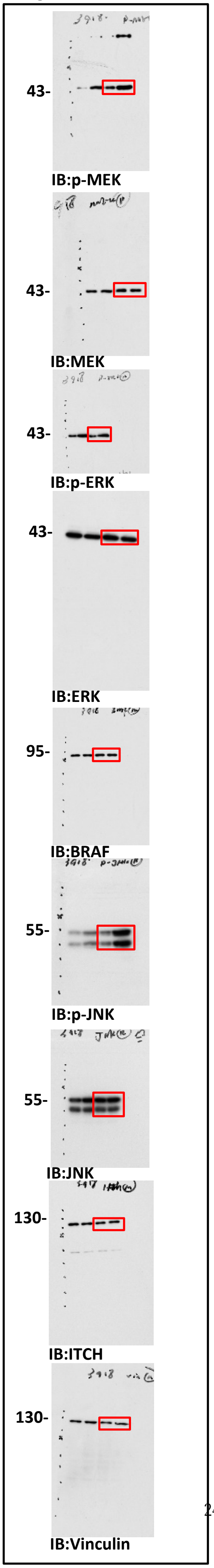

Uncropped immunoblots from Figure 4

Figure 4m

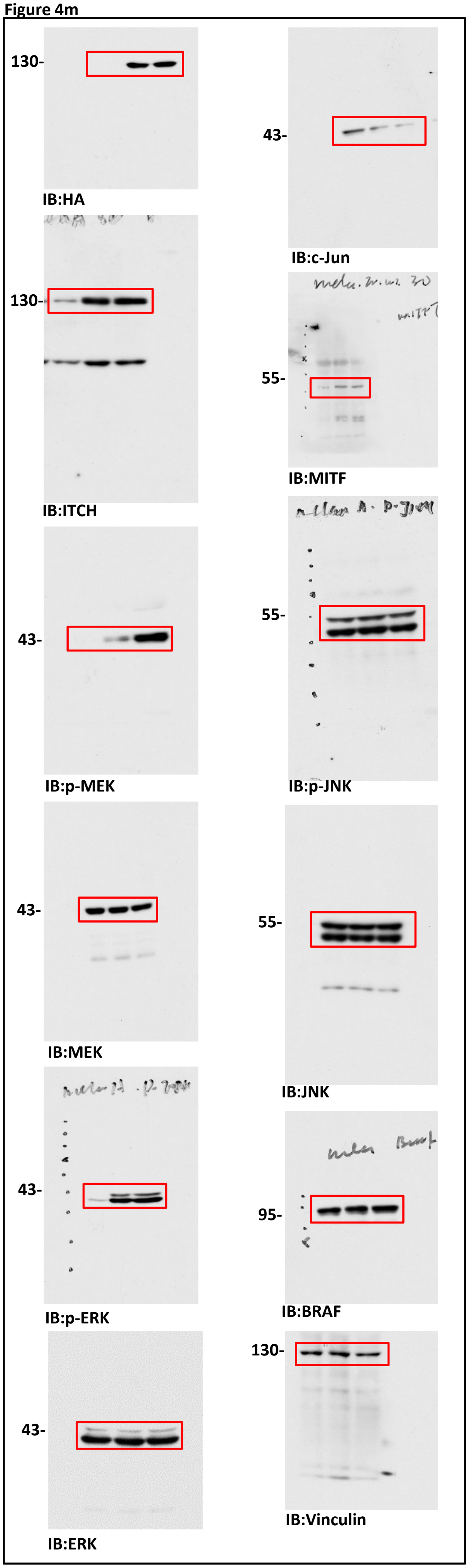

Uncropped immunoblots from Figure 4

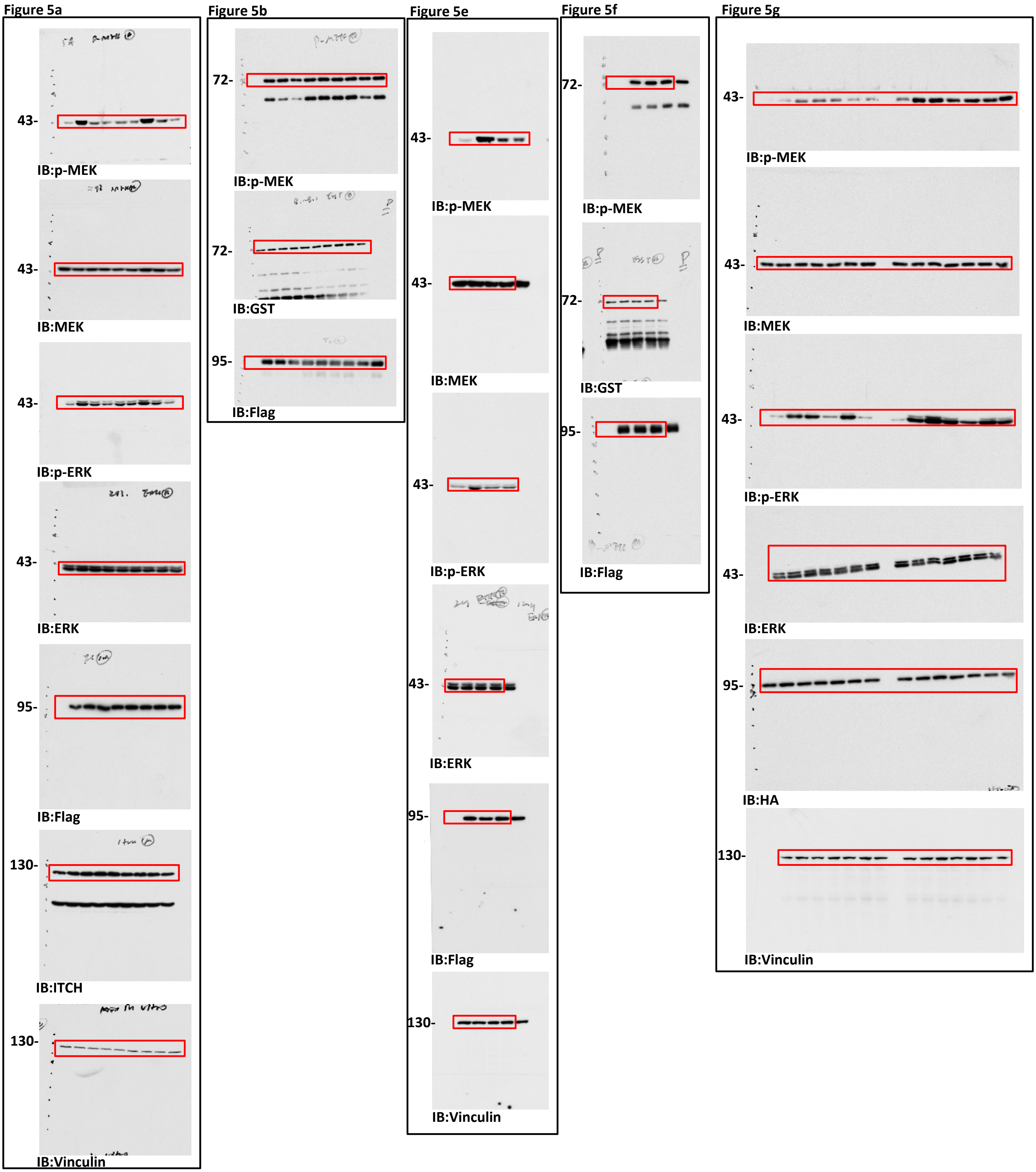

Uncropped immunoblots from Figure 5

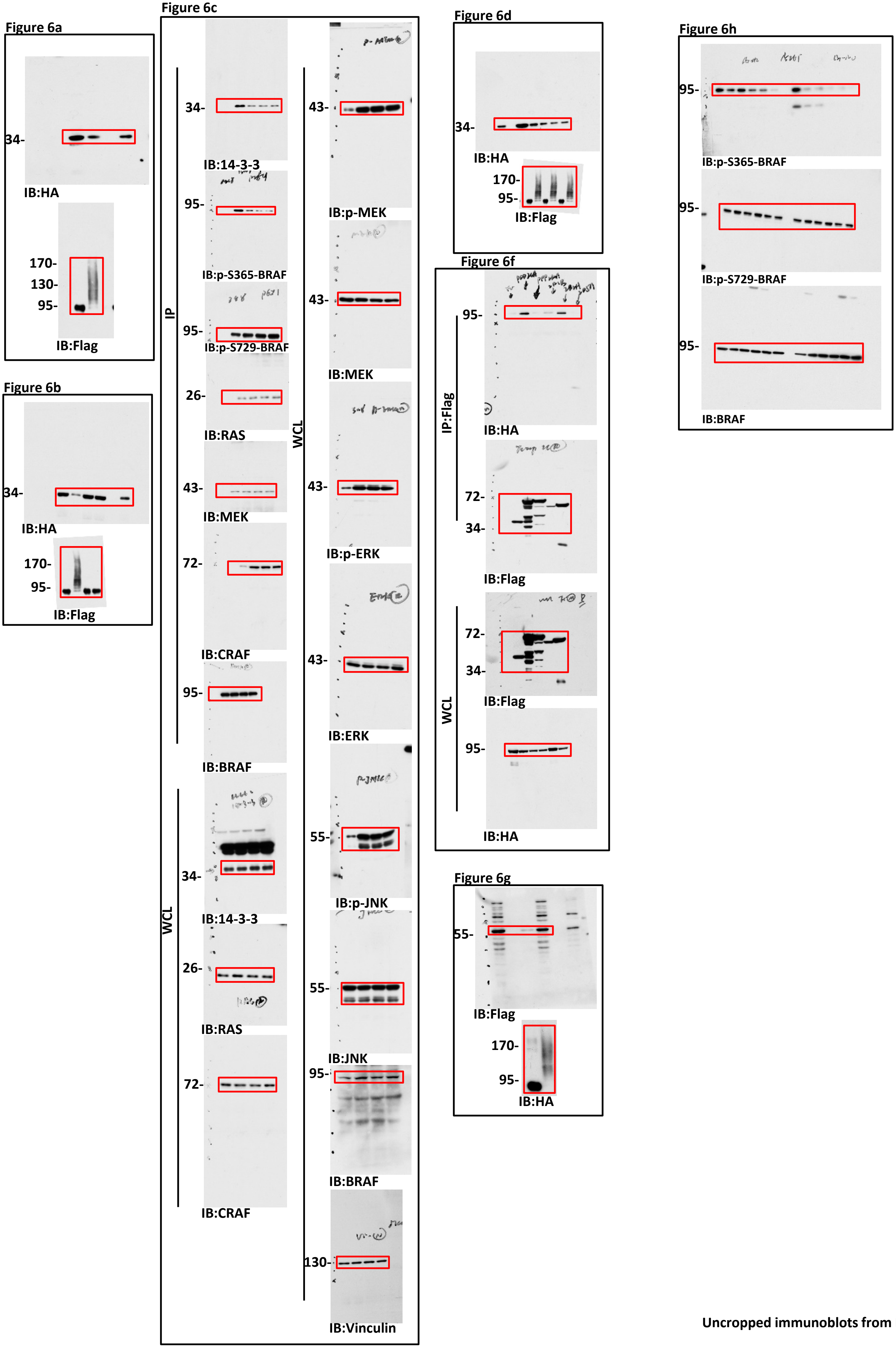

Uncropped immunoblots from Figure 6

Figure 6k

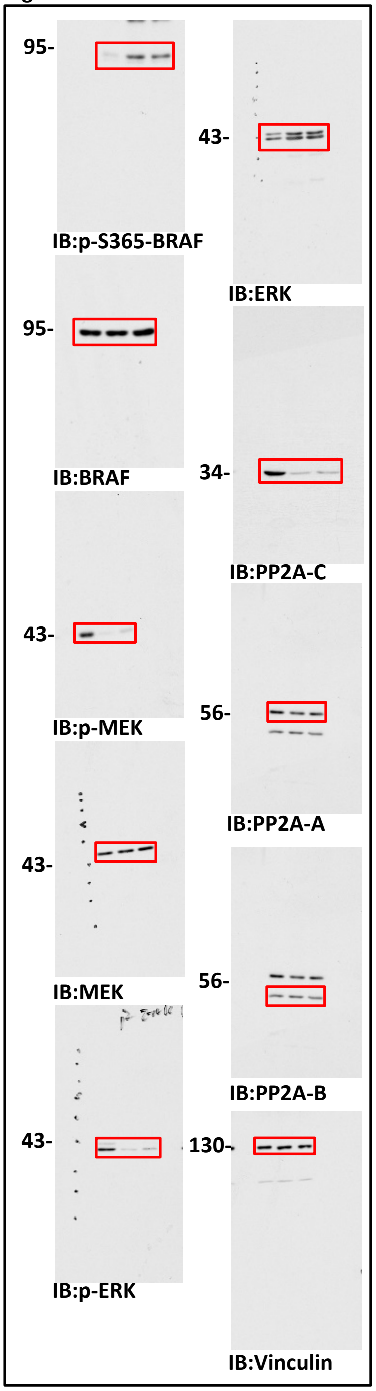

Figure 6l

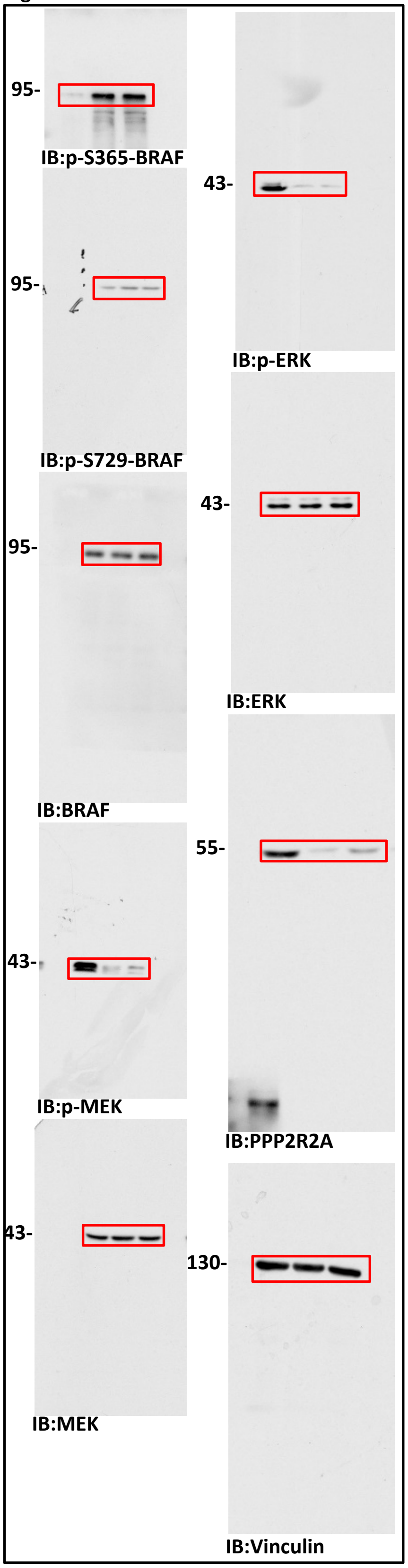

Figure 6m

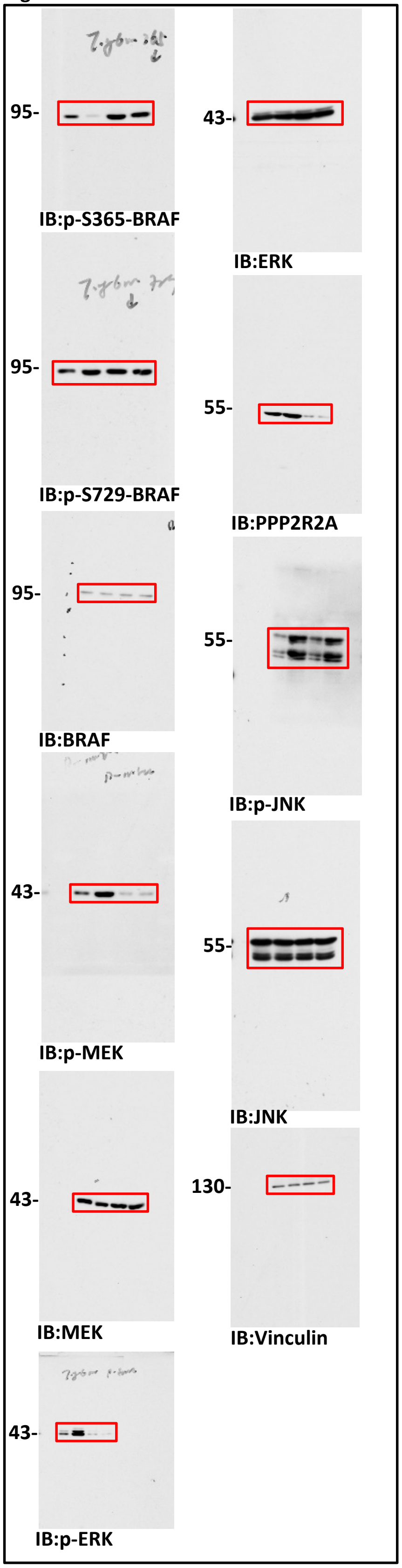

Uncropped immunoblots from Figure 6

Figure 6n

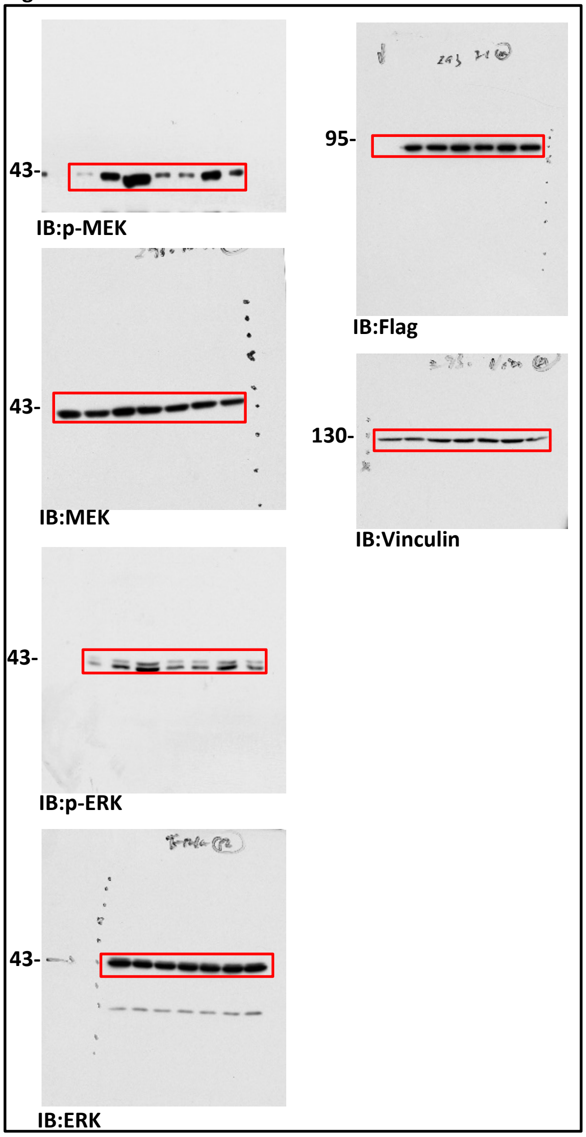

Uncropped immunoblots from Figure 6

Figure 7a

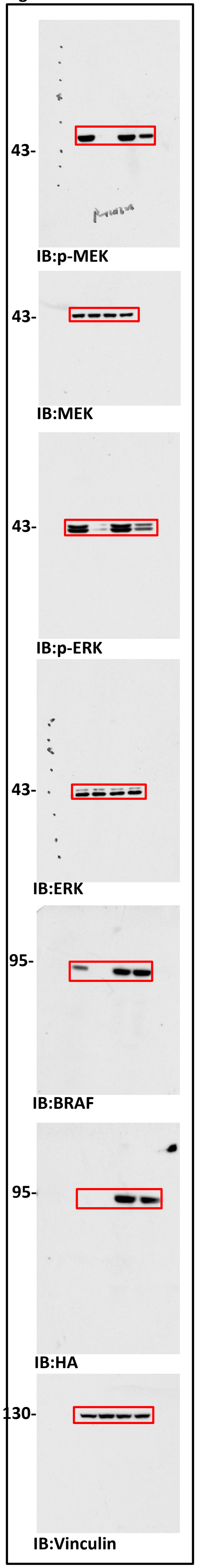

Figure 7b

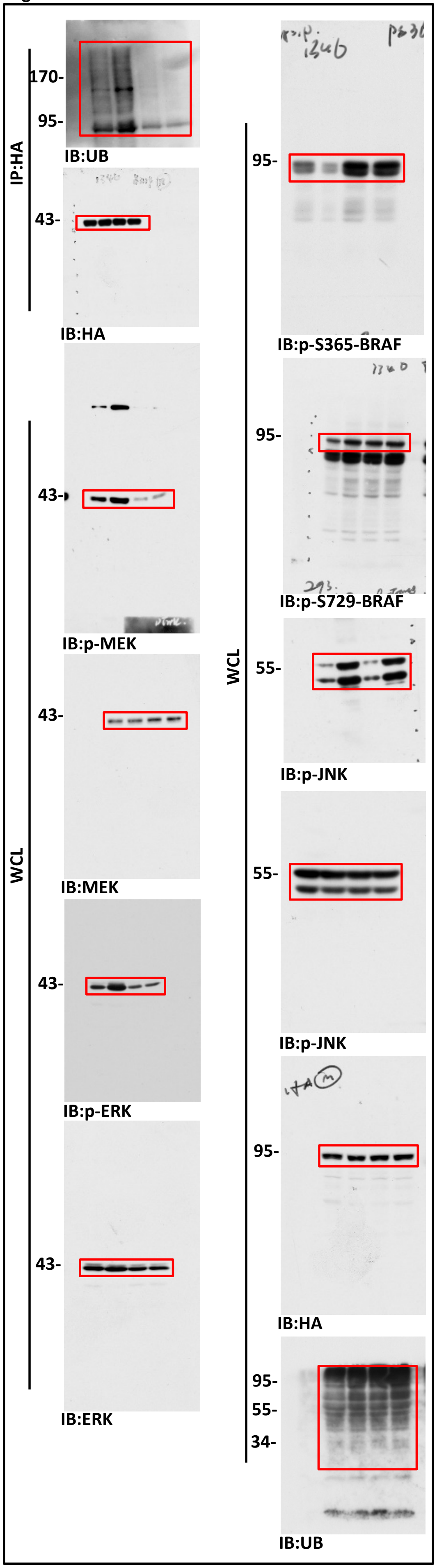

Uncropped immunoblots from Figure 7

Supplementary Figure 1a

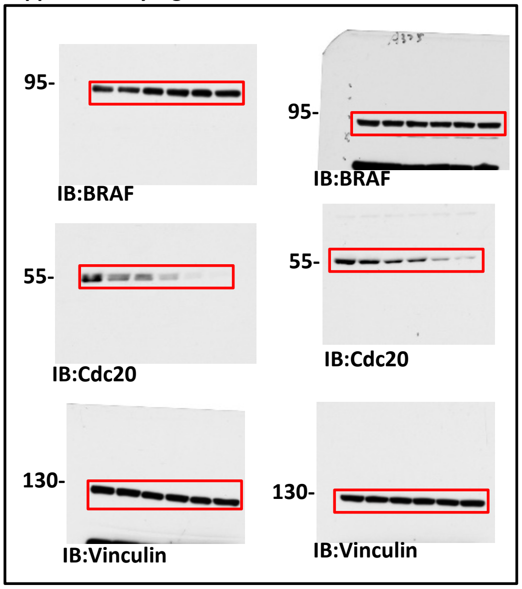

Supplementary Figure 1b

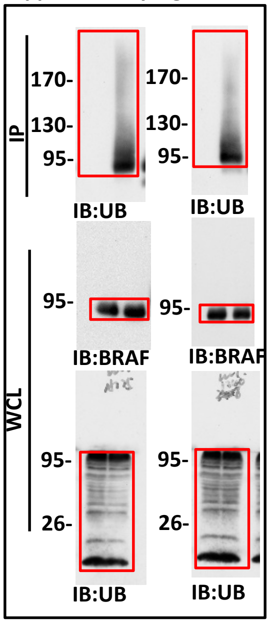

Supplementary Figure 1c

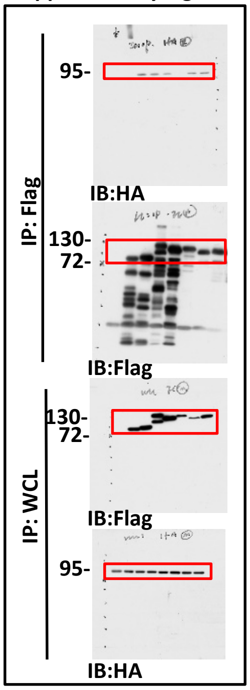

Supplementary Figure 1d

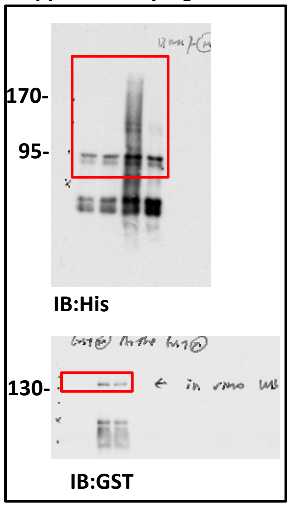

Supplementary Figure 1e

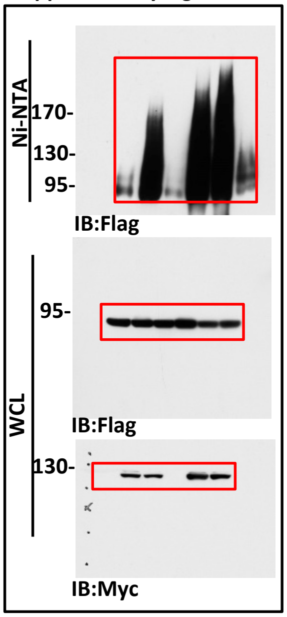

Supplementary Figure 1f

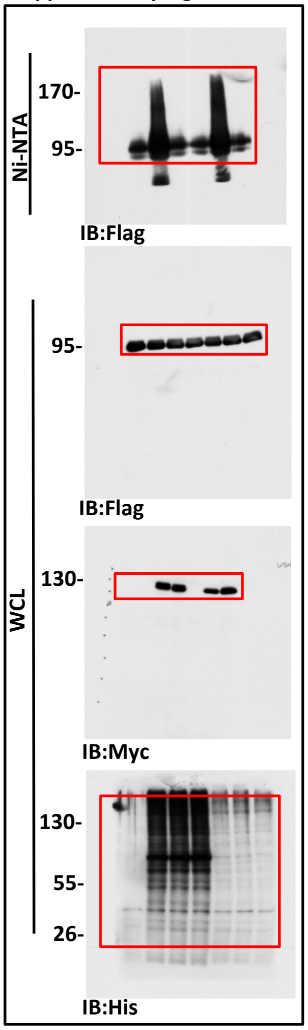

Supplementary Figure 1g

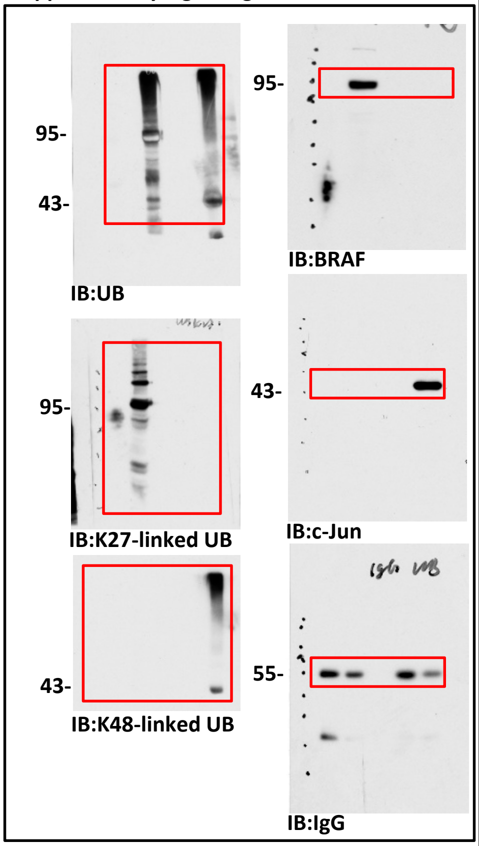

Supplementary Figure 1h

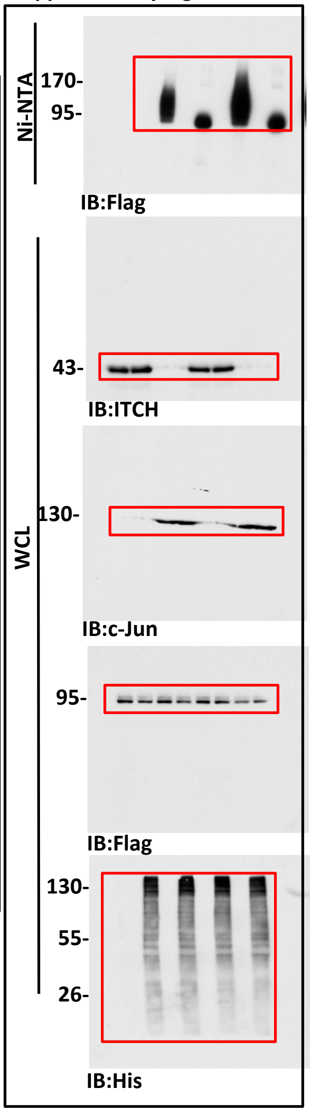

Uncropped immunoblots from Supplementary Figure 1

Supplementary Figure 3b

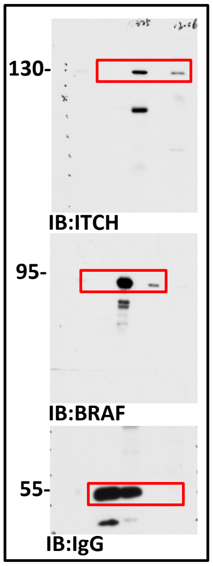

Supplementary Figure 3c

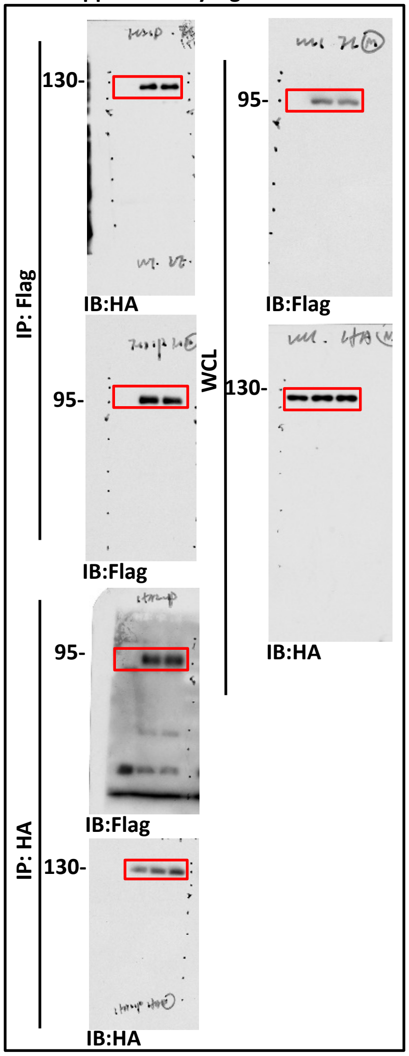

Supplementary Figure 3d

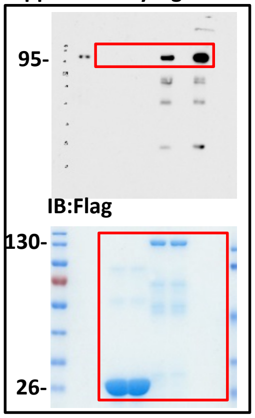

Supplementary Figure 3e

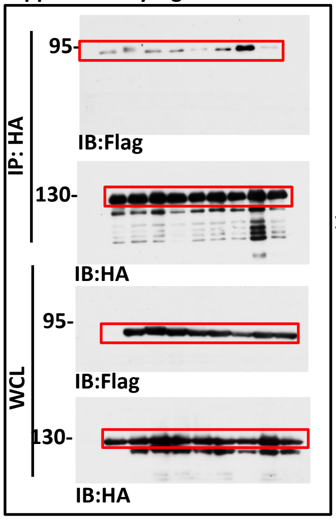

Supplementary Figure 3f

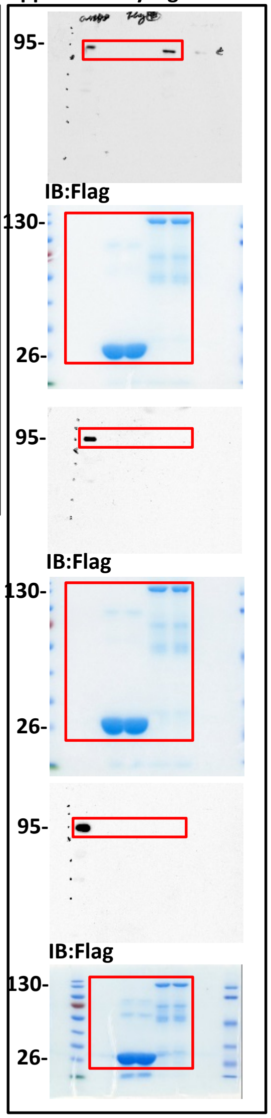

Supplementary Figure 3j

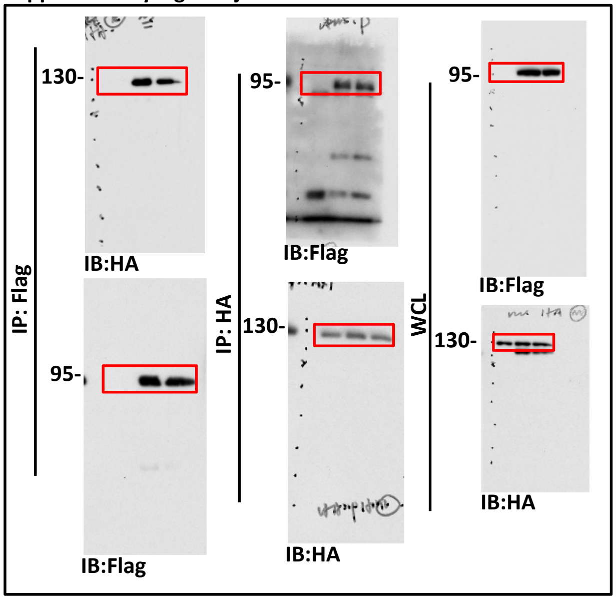

Supplementary Figure 3g

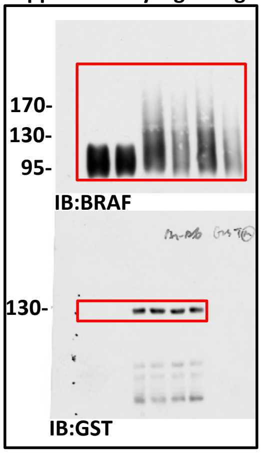

Uncropped immunoblots from Supplementary Figure 3

Supplementary Figure 4a

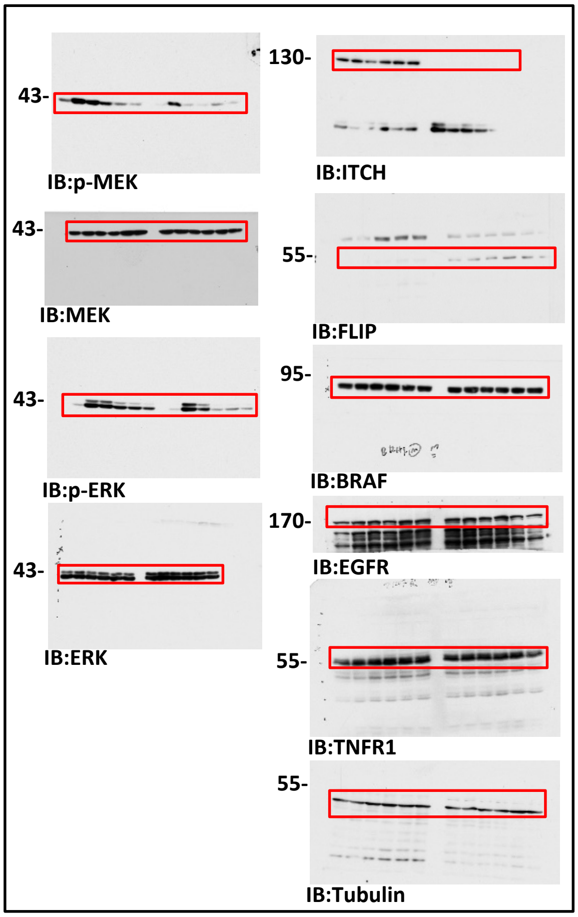

Supplementary Figure 4b

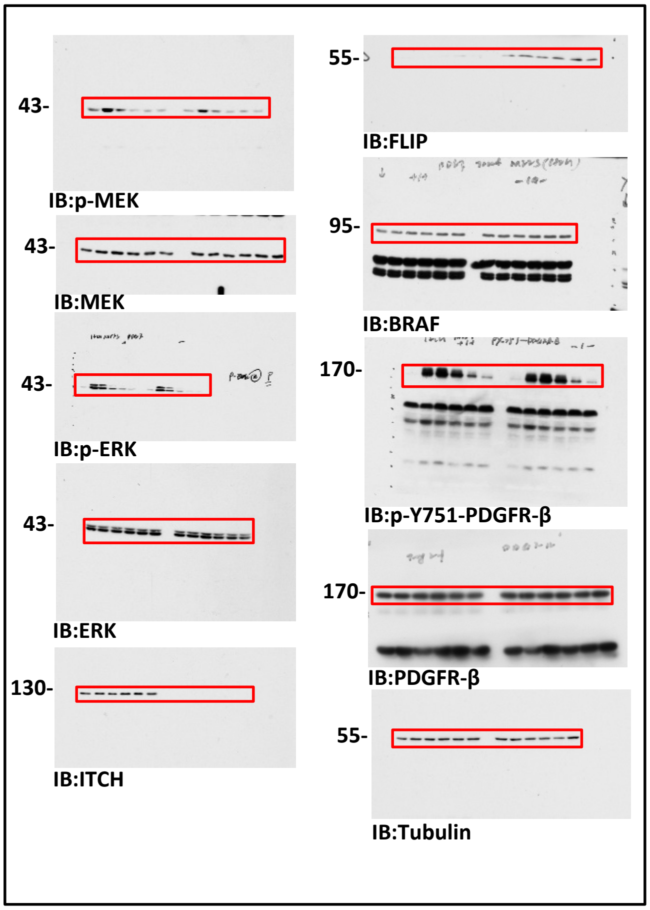

Supplementary Figure 4c

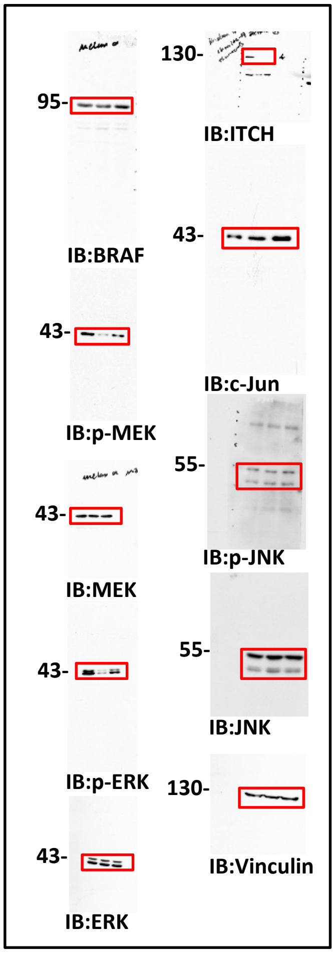

Supplementary Figure 4d

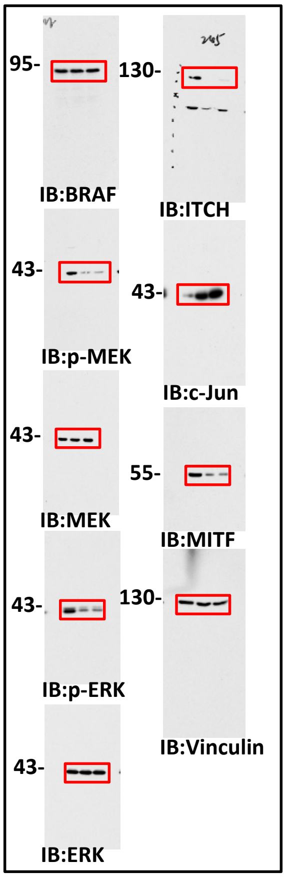

Supplementary Figure 4e

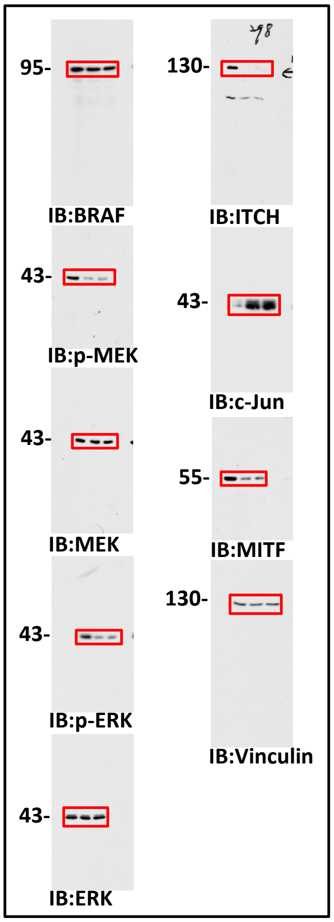

Uncropped immunoblots from Supplementary Figure 4

Supplementary Figure 4f

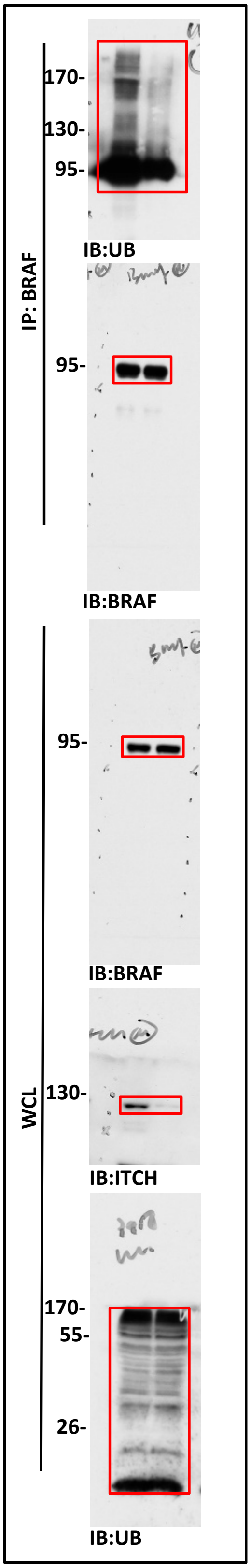

Supplementary Figure 4g

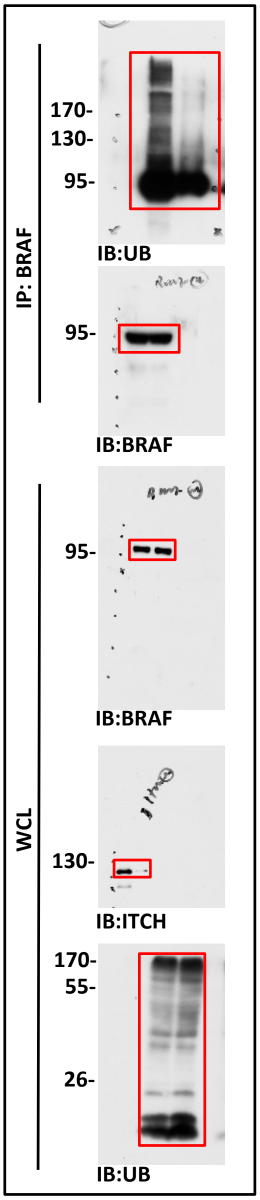

Supplementary Figure 4h

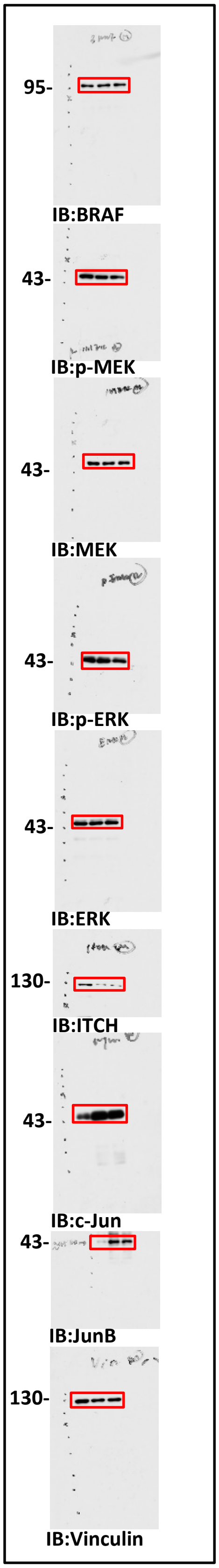

Supplementary Figure 4i

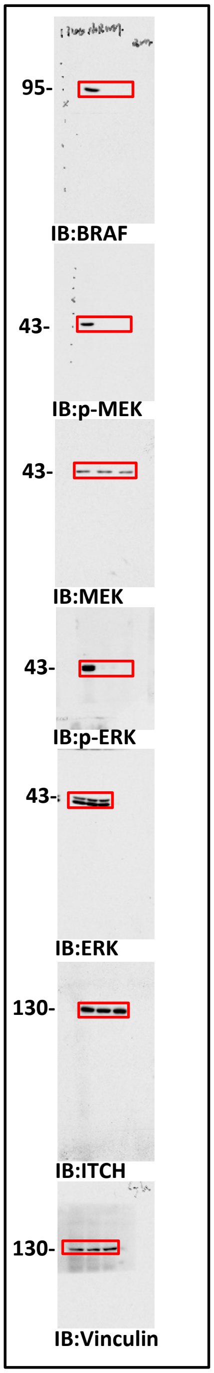

Uncropped immunoblots from Supplementary Figure 4

Supplementary Figure 5a

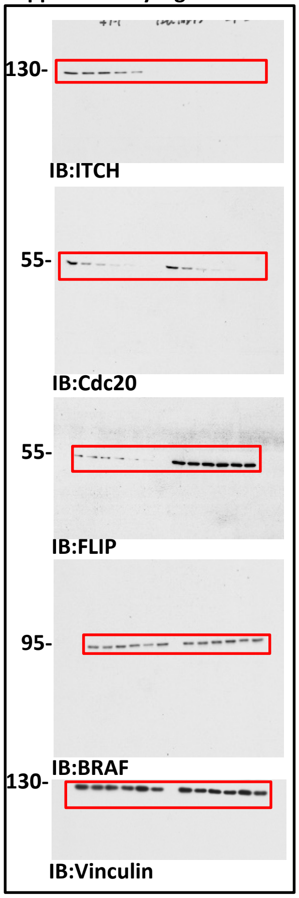

Supplementary Figure 5b

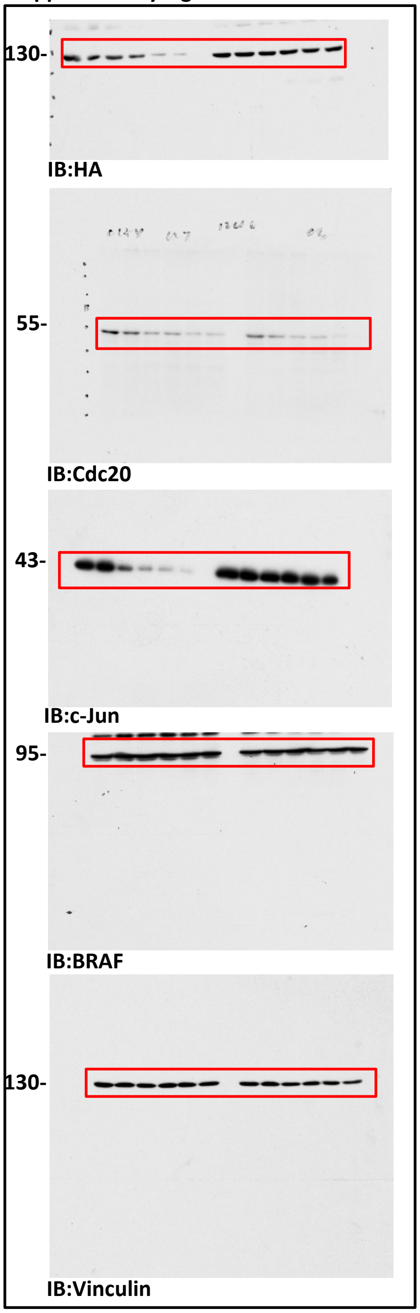

Supplementary Figure 5c

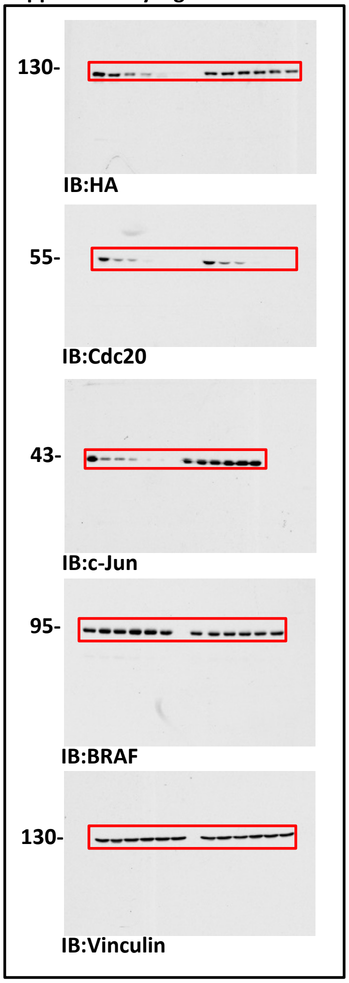

Supplementary Figure 5d

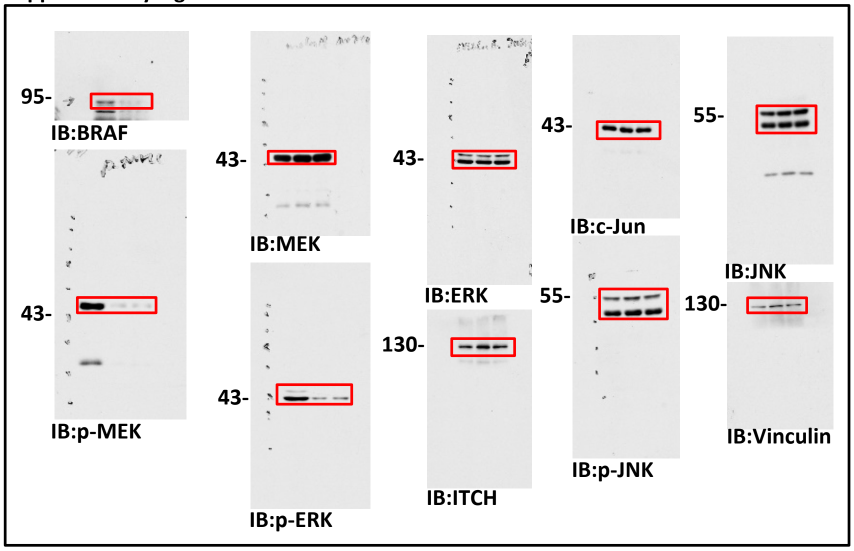

Uncropped immunoblots from Supplementary Figure 5

Supplementary Figure 6a

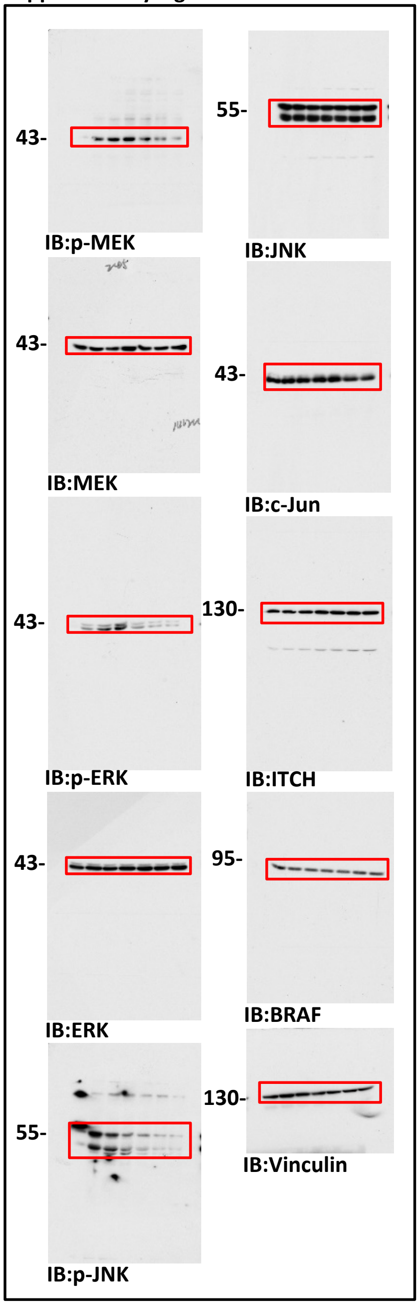

Supplementary Figure 6b

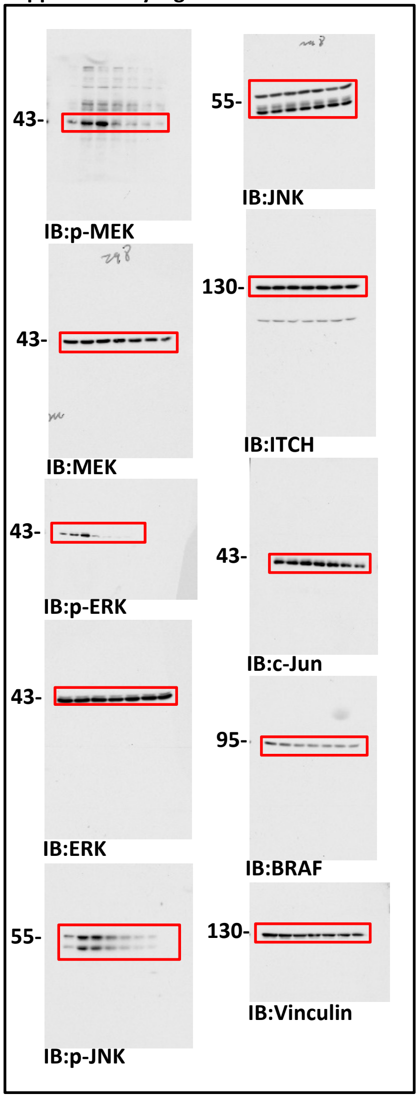

Supplementary Figure 6c

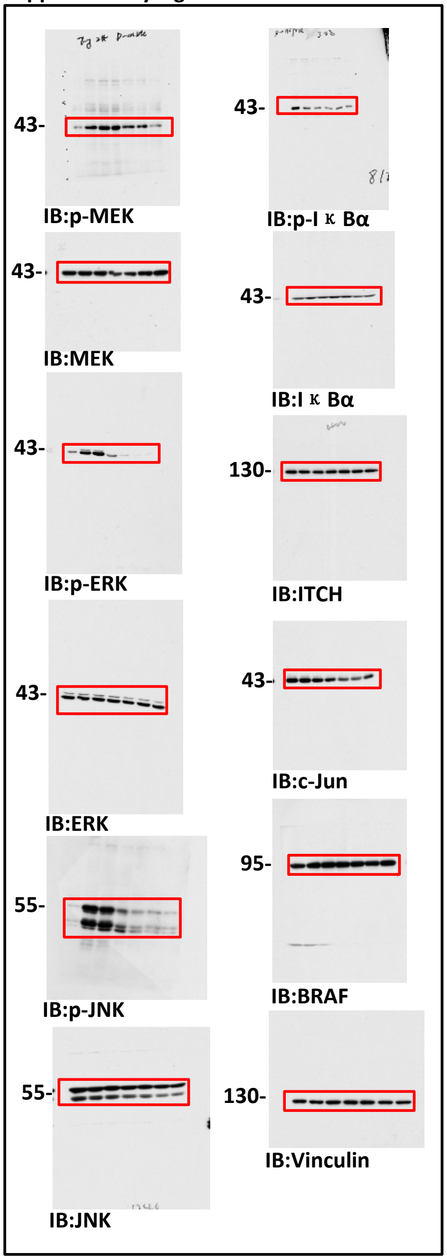

43

Uncropped immunoblots from Supplementary Figure 6

Supplementary Figure 6d

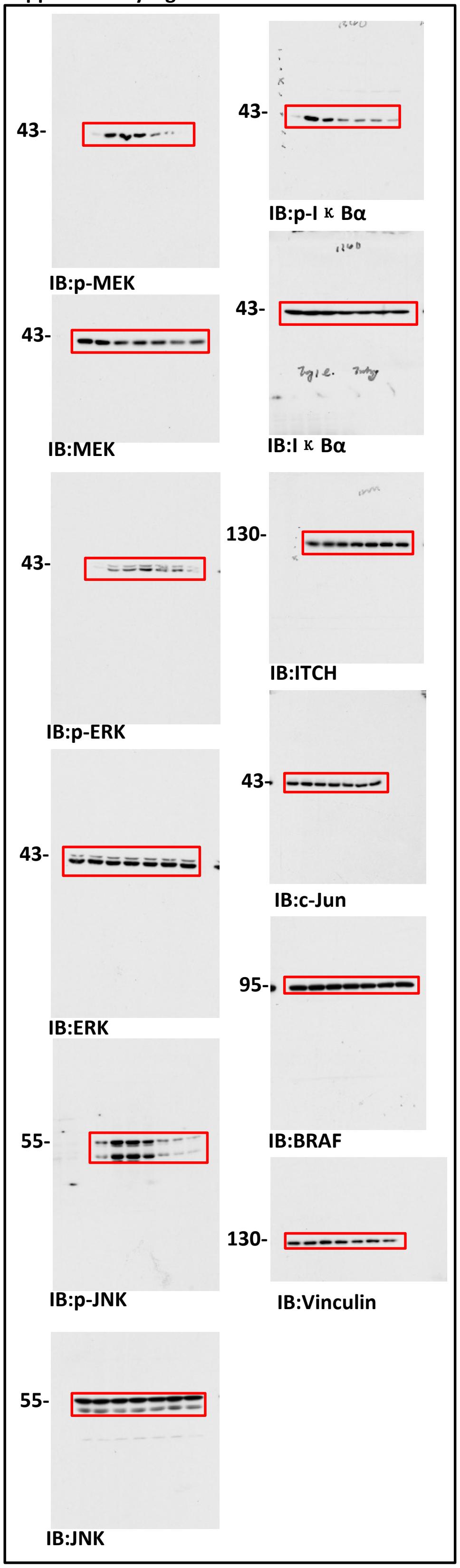

Supplementary Figure 6e

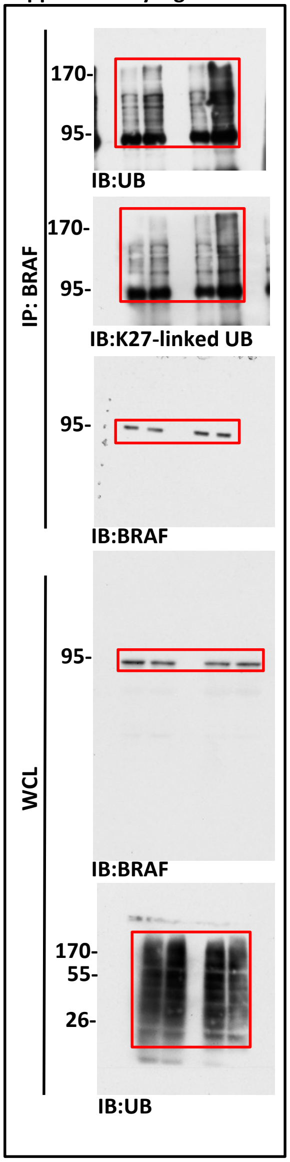

Supplementary Figure 6f

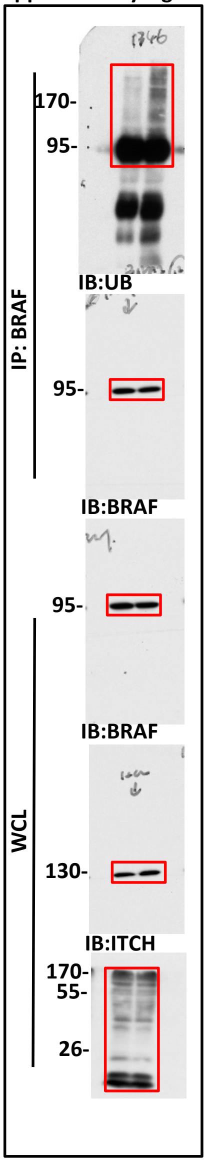

Supplementary Figure 6g

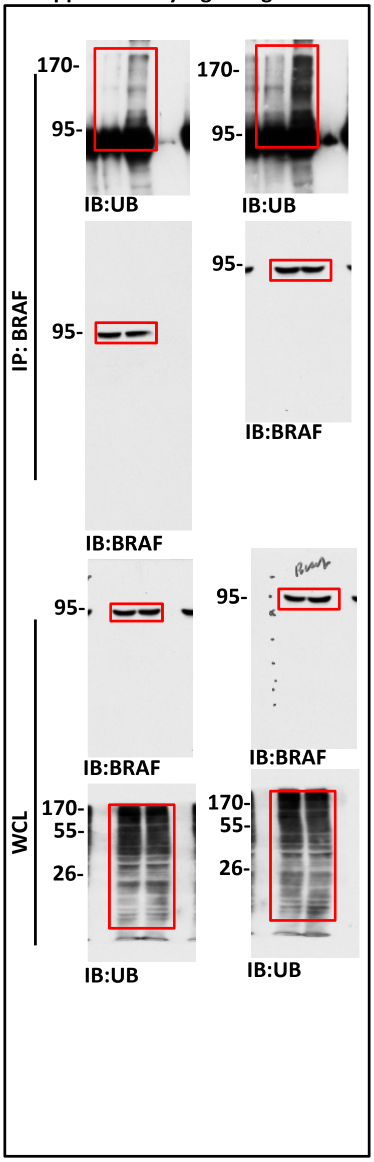

Uncropped immunoblots from Supplementary Figure 6

Supplementary Figure 6h

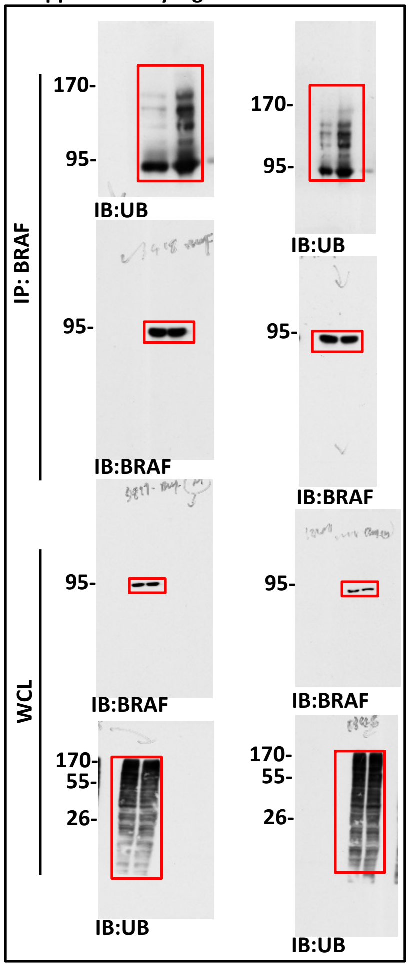

Supplementary Figure 6i

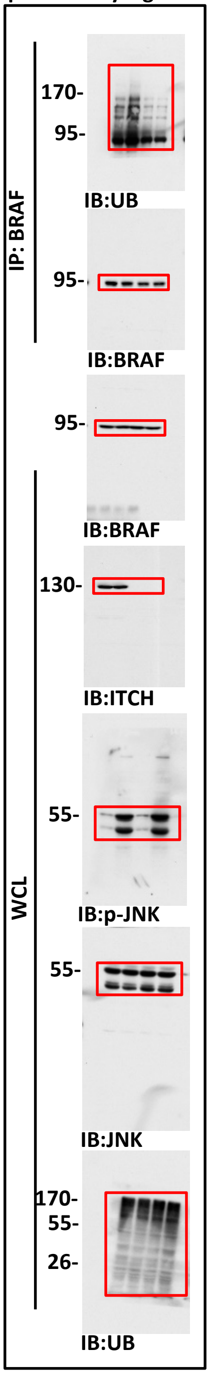

Supplementary Figure 6j

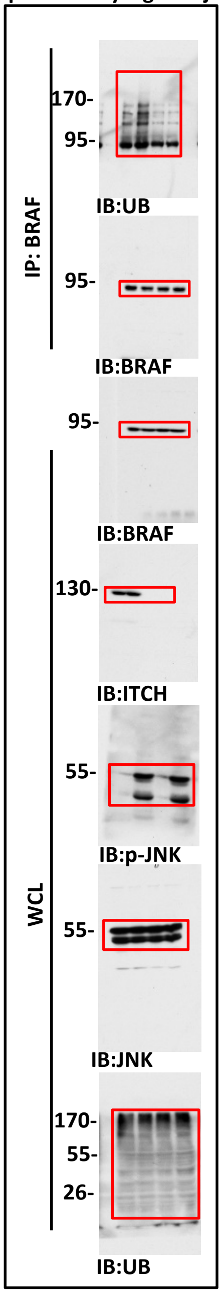

Supplementary Figure 6k

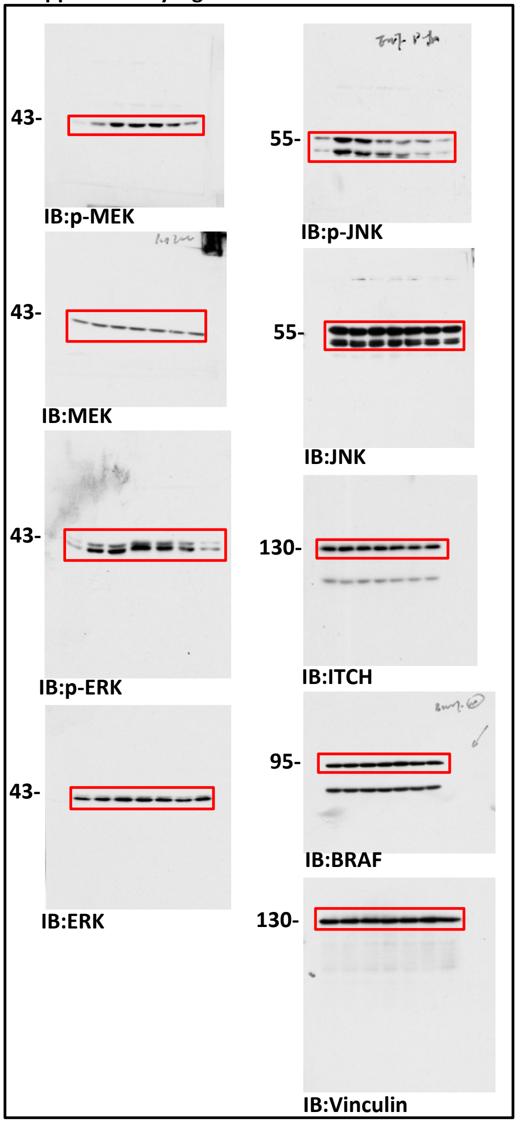

Uncropped immunoblots from Supplementary Figure 6

Supplementary Figure 6l

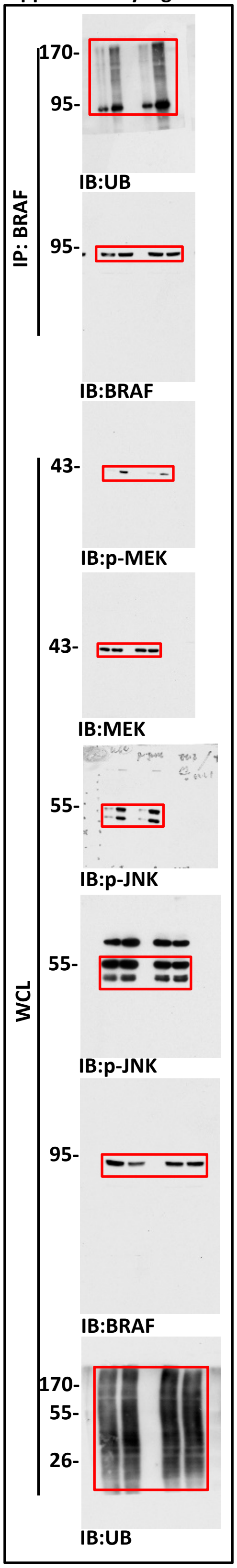

Supplementary Figure 6m

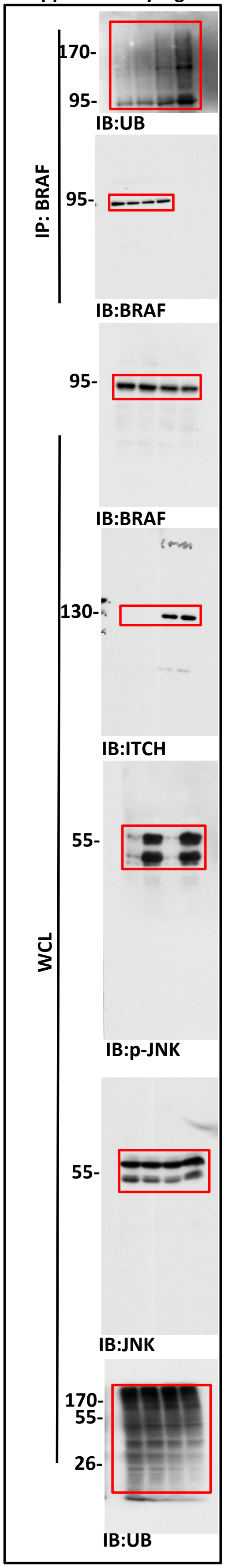

Supplementary Figure 6n

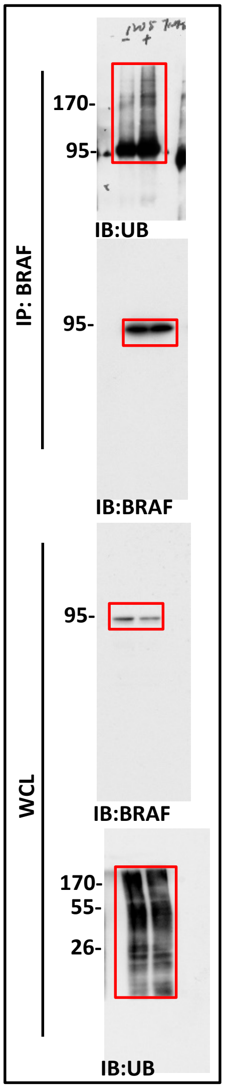

Supplementary Figure 6o

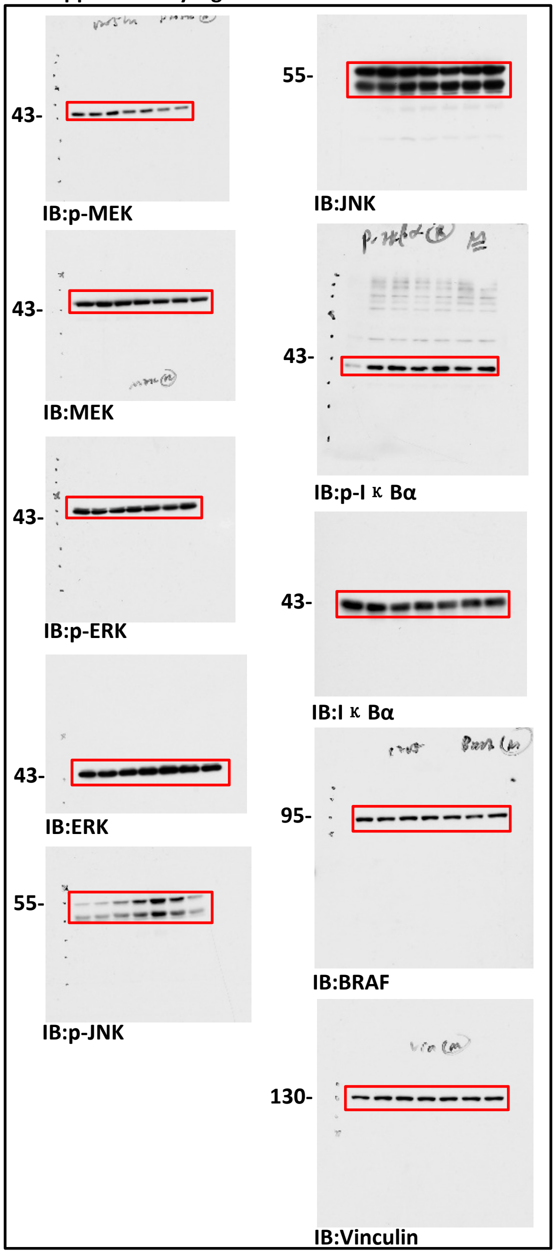

Uncropped immunoblots from Supplementary Figure 6

Supplementary Figure 6p

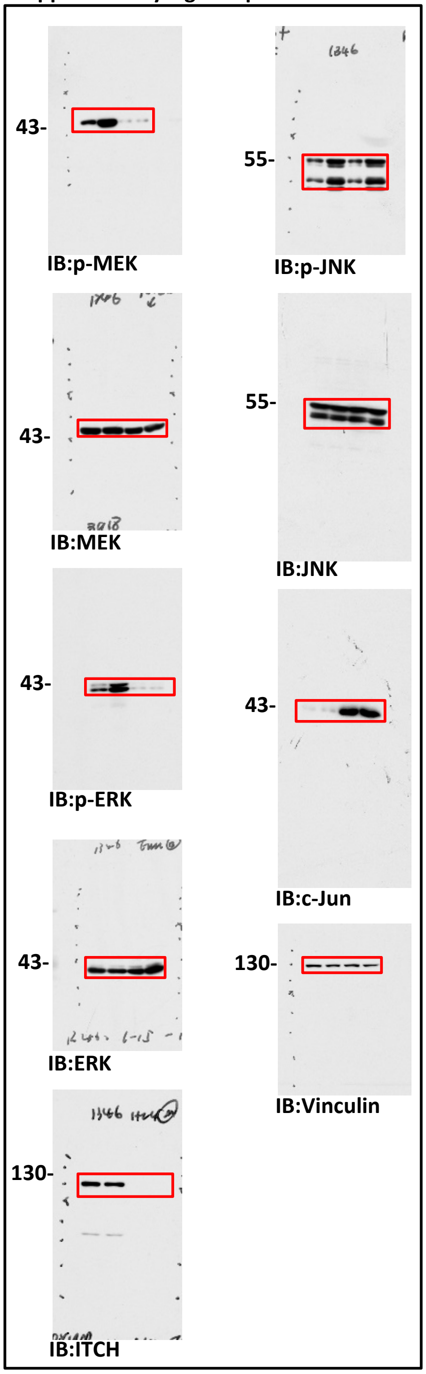

Uncropped immunoblots from Supplementary Figure 6

Supplementary Figure 7a

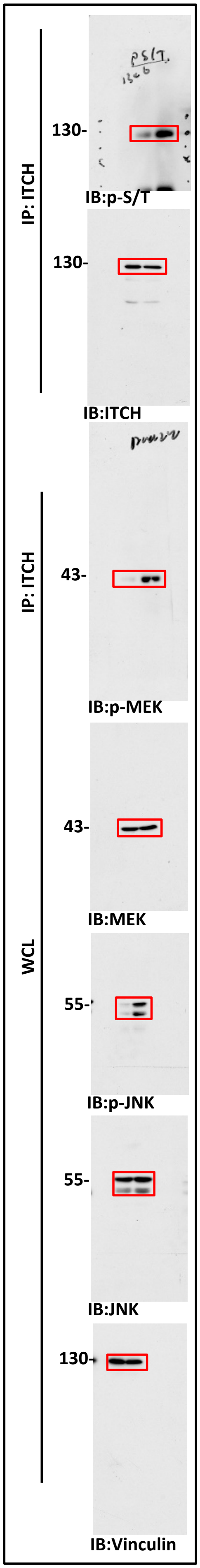

Supplementary Figure 7c

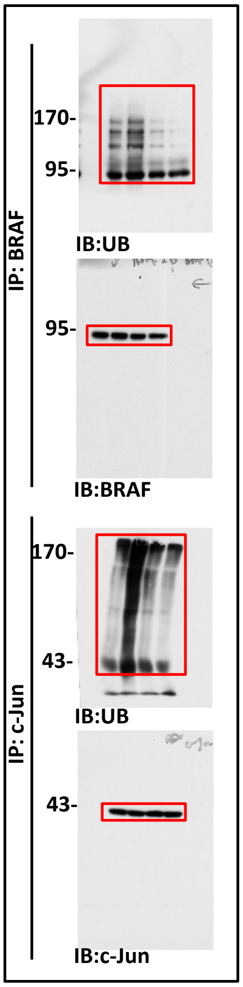

Supplementary Figure 7d

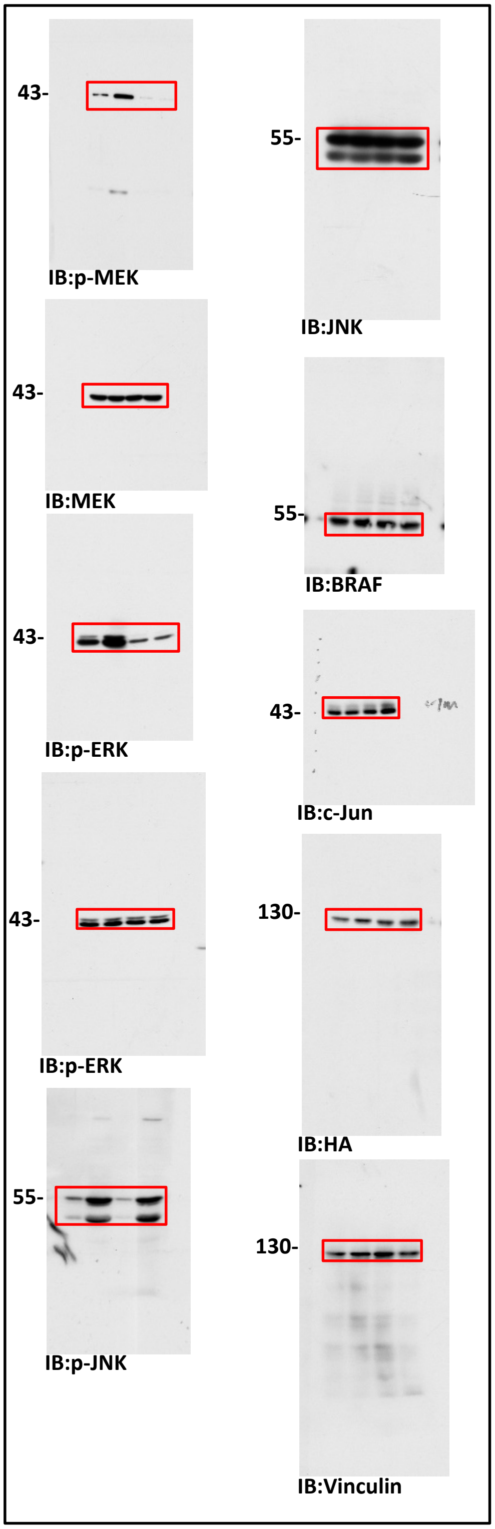

Uncropped immunoblots from Supplementary Figure 7

Supplementary Figure 7o

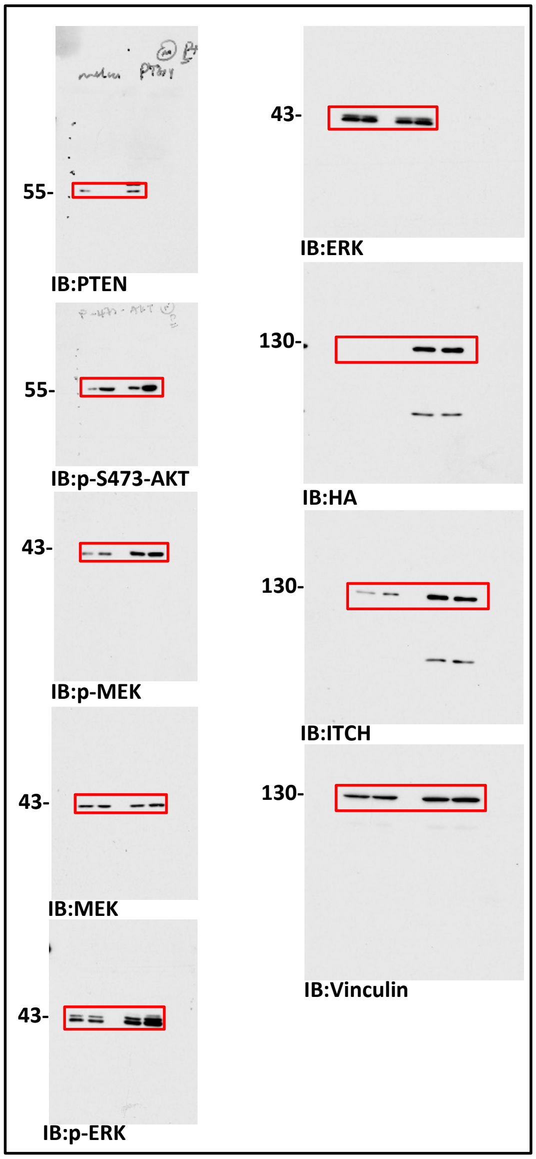

Uncropped immunoblots from Supplementary Figure 7

Supplementary Figure 8a

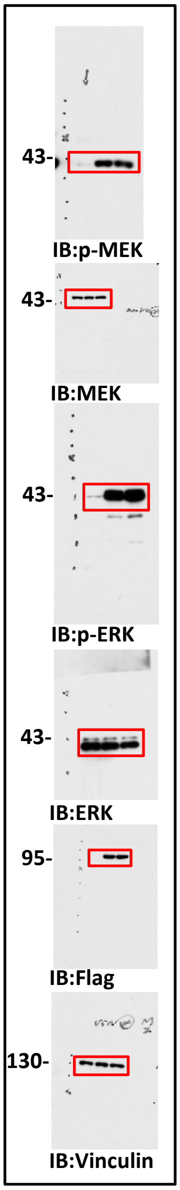

Supplementary Figure 8c

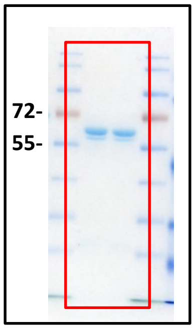

Supplementary Figure 8e

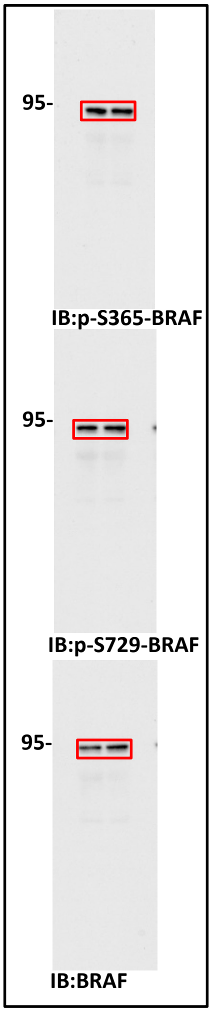

Supplementary Figure 8f

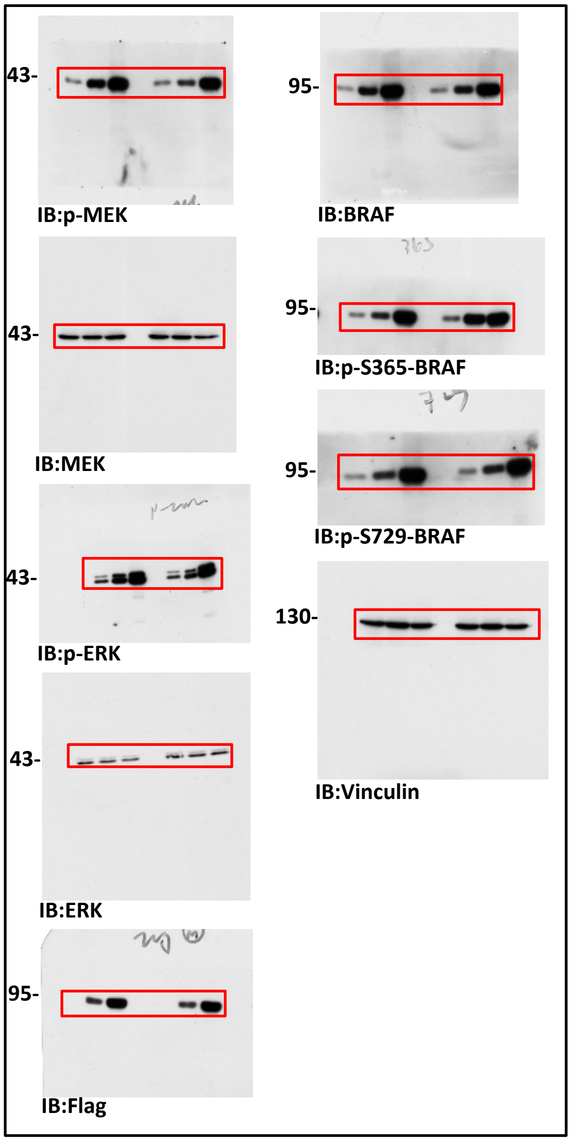

Supplementary Figure 8b

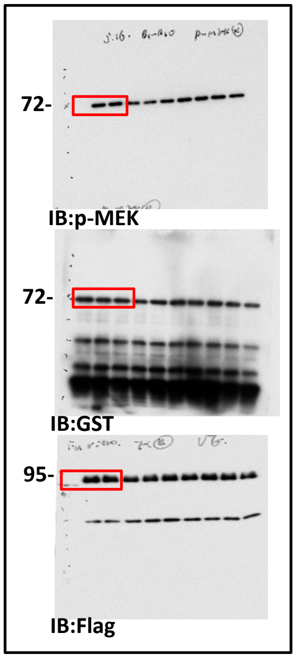

Supplementary Figure 8g

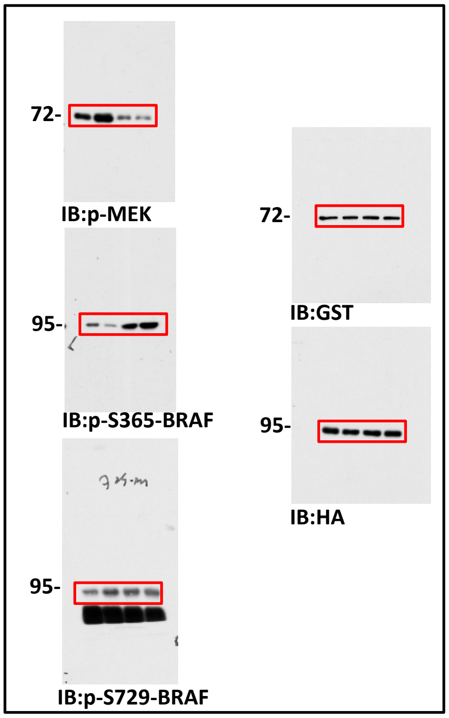

Uncropped immunoblots from Supplementary Figure 8

Supplementary Figure 9a

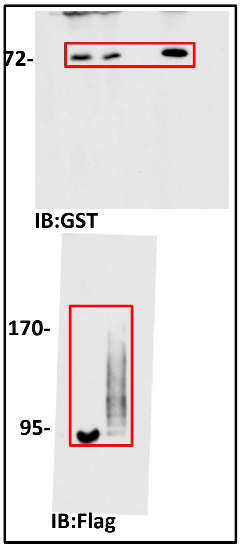

Supplementary Figure 9b

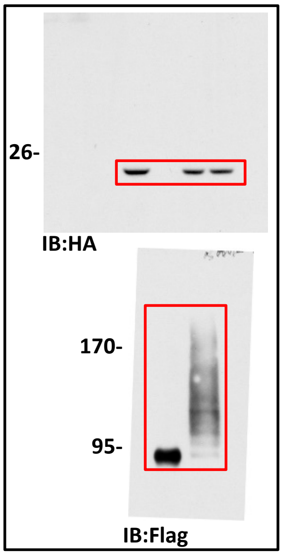

Supplementary Figure 9c

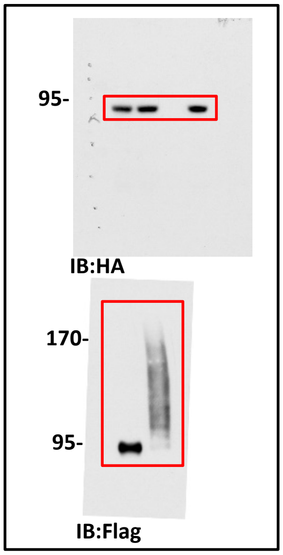

Supplementary Figure 9d

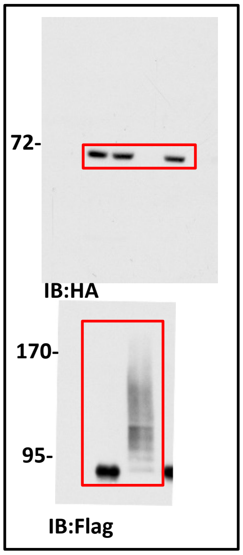

Supplementary Figure 9e

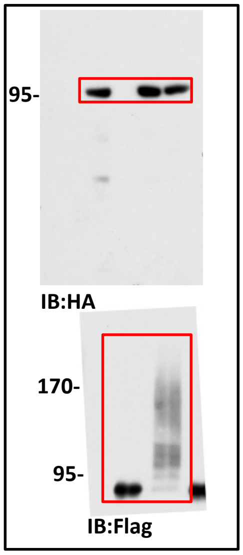

Supplementary Figure 9f

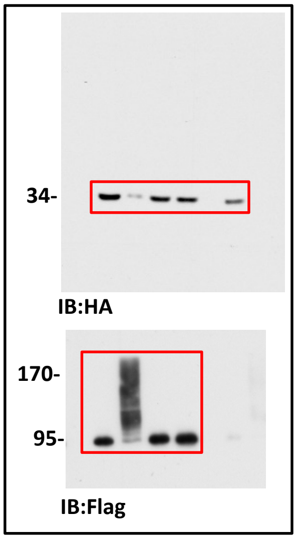

Supplementary Figure 9g

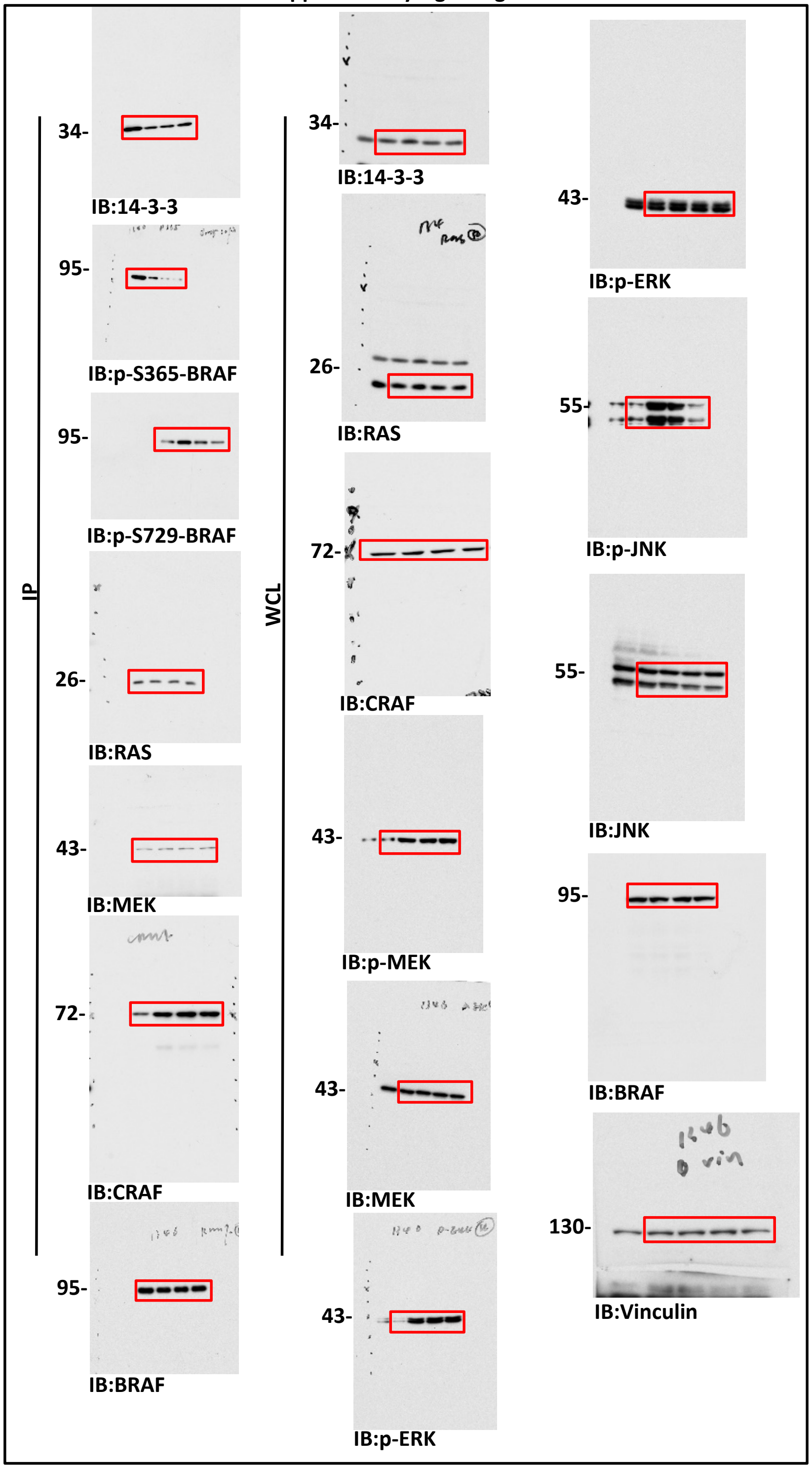

Uncropped immunoblots from Supplementary Figure 9

Supplementary Figure 9i

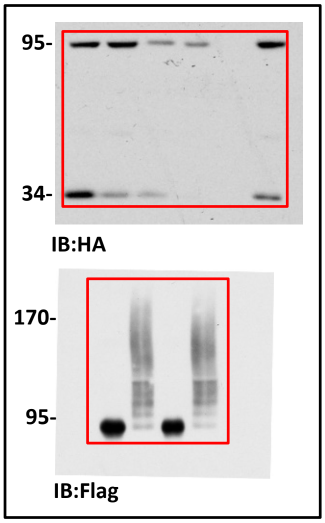

Supplementary Figure 9k

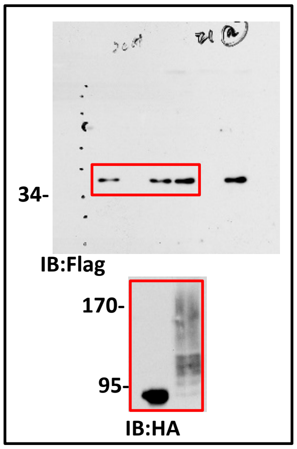

Supplementary Figure 9m

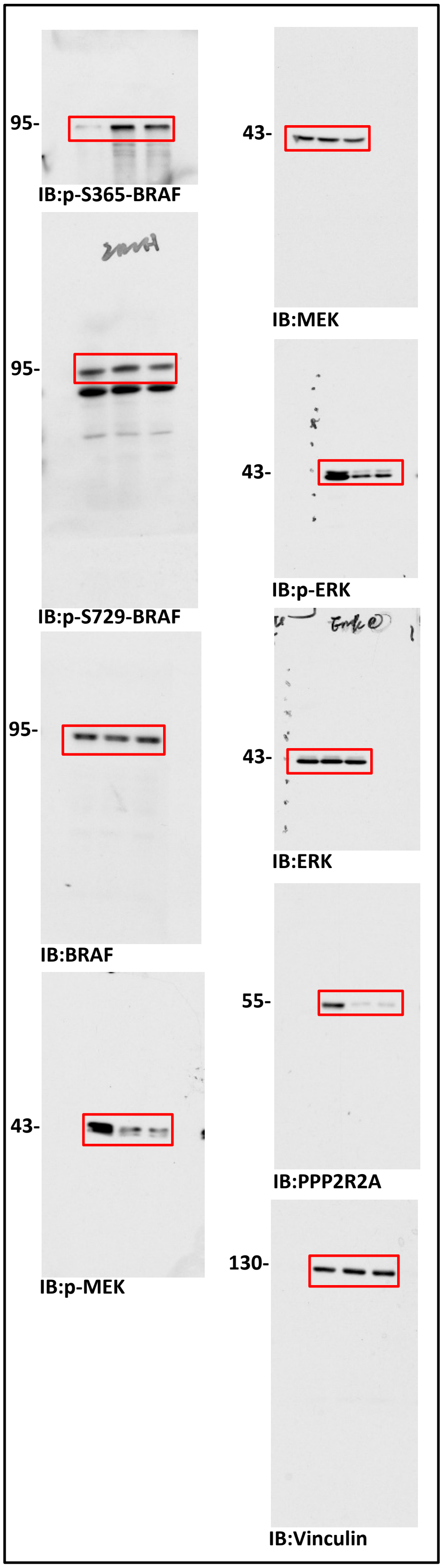

Supplementary Figure 9n

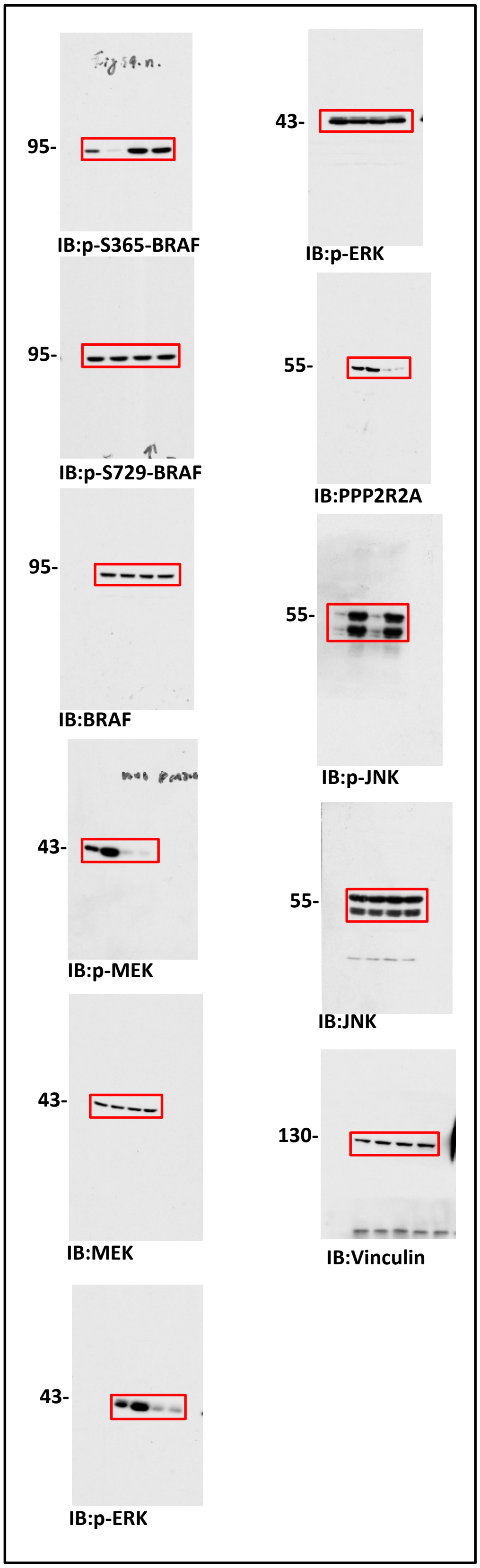

Uncropped immunoblots from Supplementary Figure 9

Supplementary Figure 9o

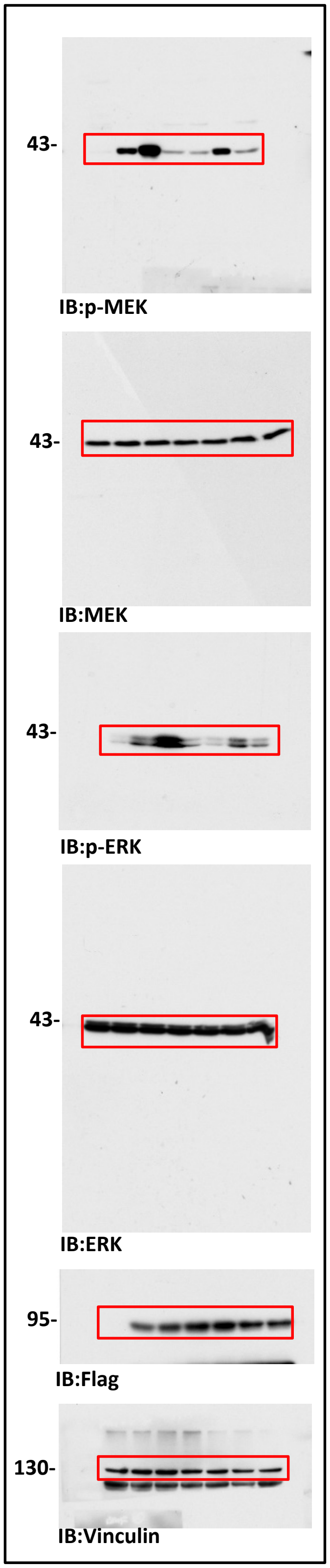

Supplementary Figure 9p

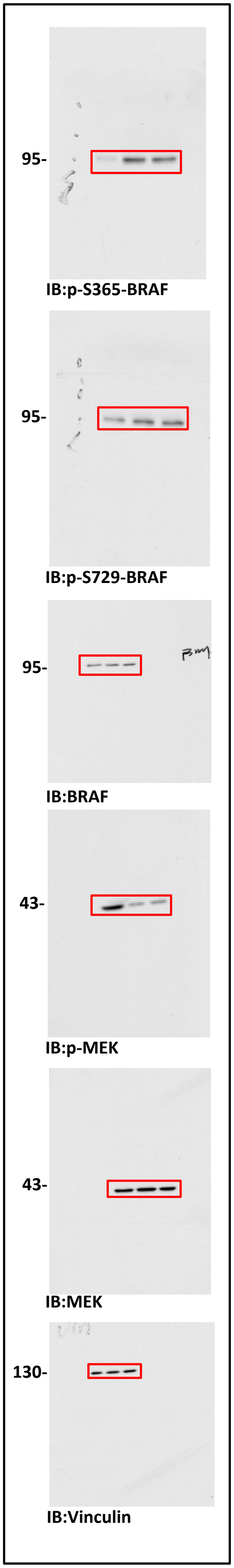

Uncropped immunoblots from Supplementary Figure 9

Supplementary Figure 10a

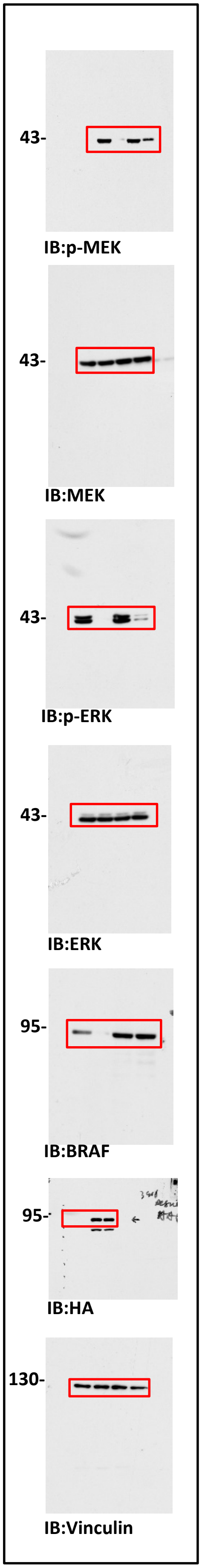

Supplementary Figure 10b

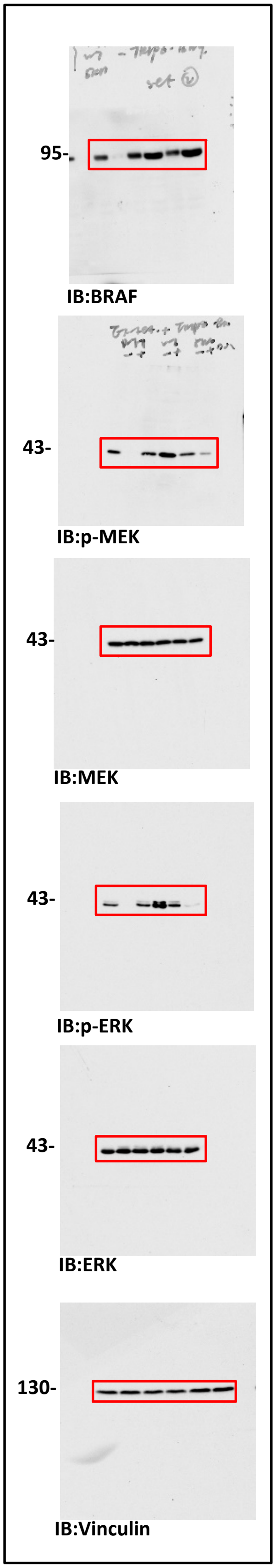

Supplementary Figure 10c

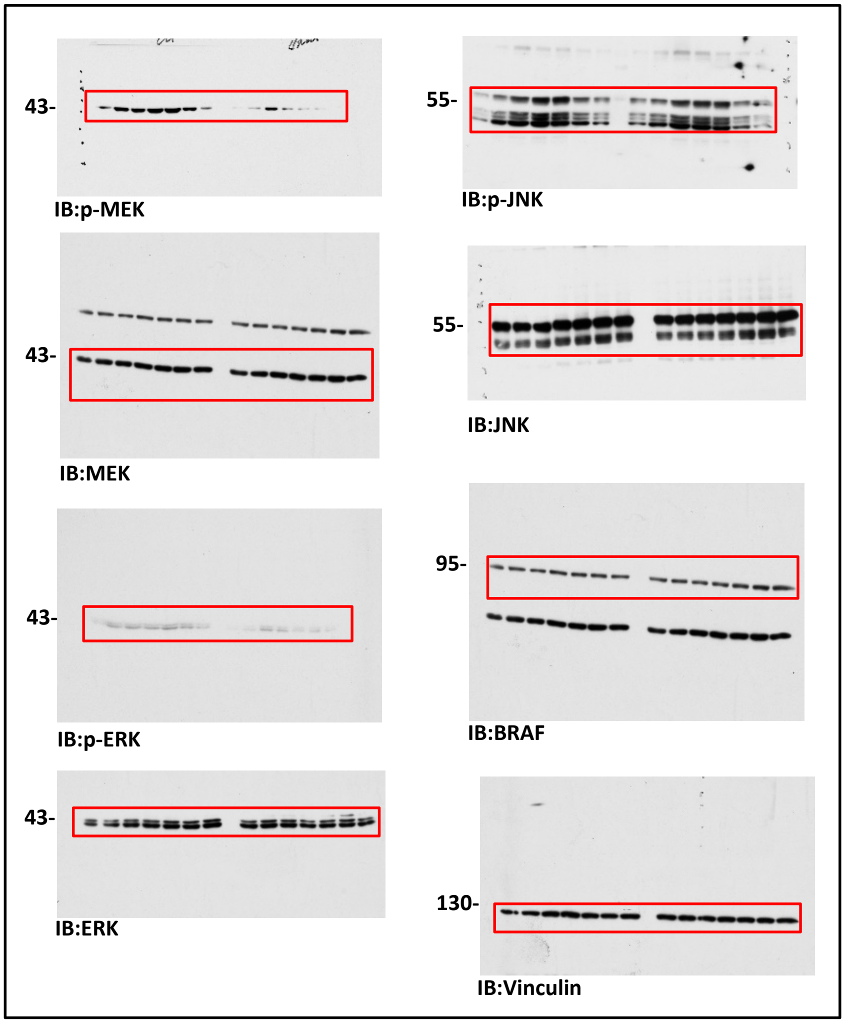

Uncropped immunoblots from Supplementary Figure 10

Supplementary Figure 10d

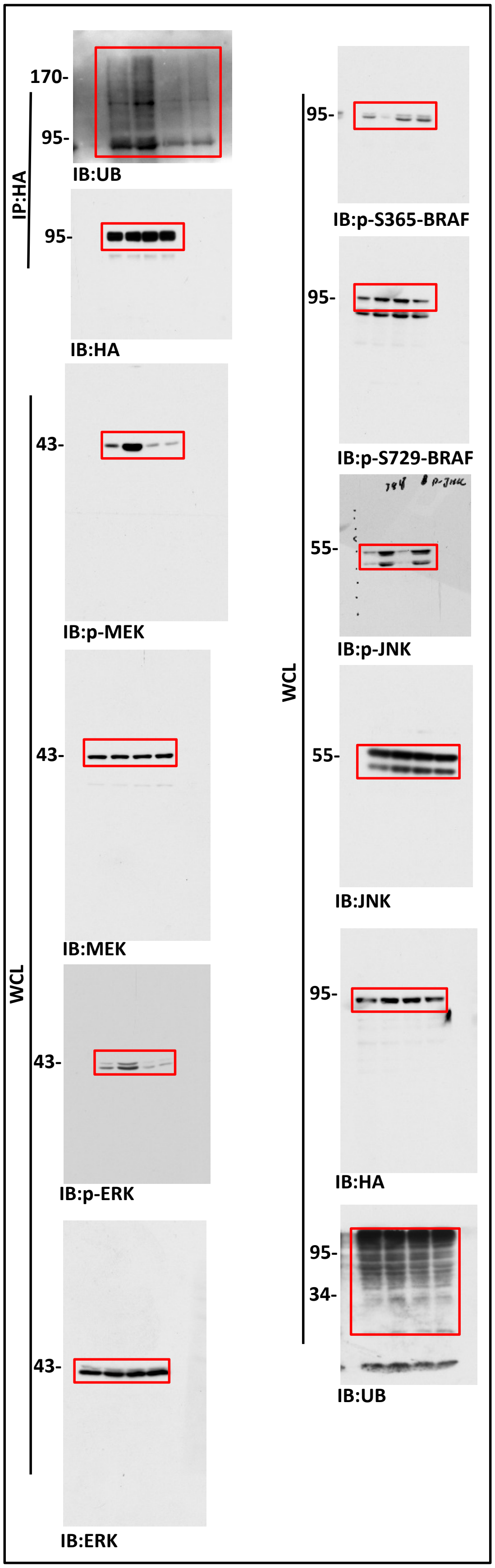

Supplementary Figure 10e

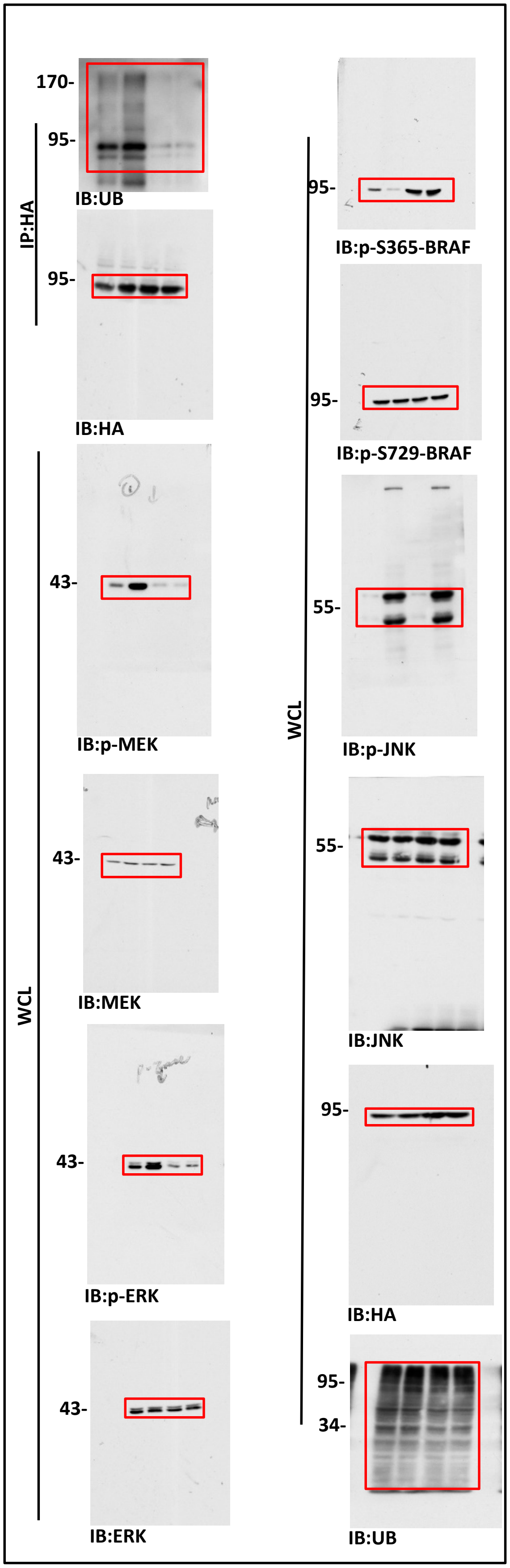

Uncropped immunoblots from Supplementary Figure 10

Supplementary Figure 11k

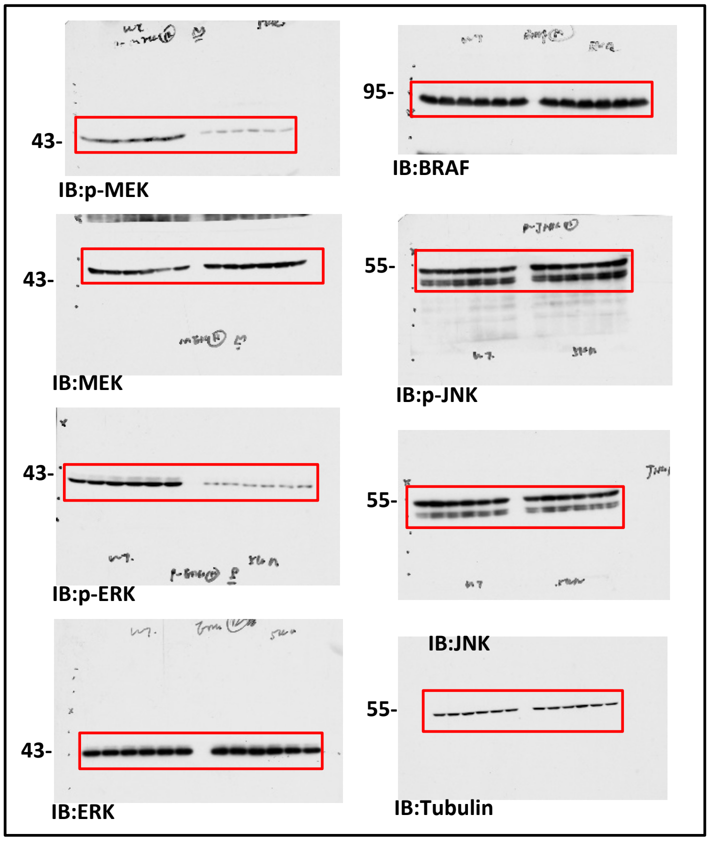

Supplementary Figure 11l

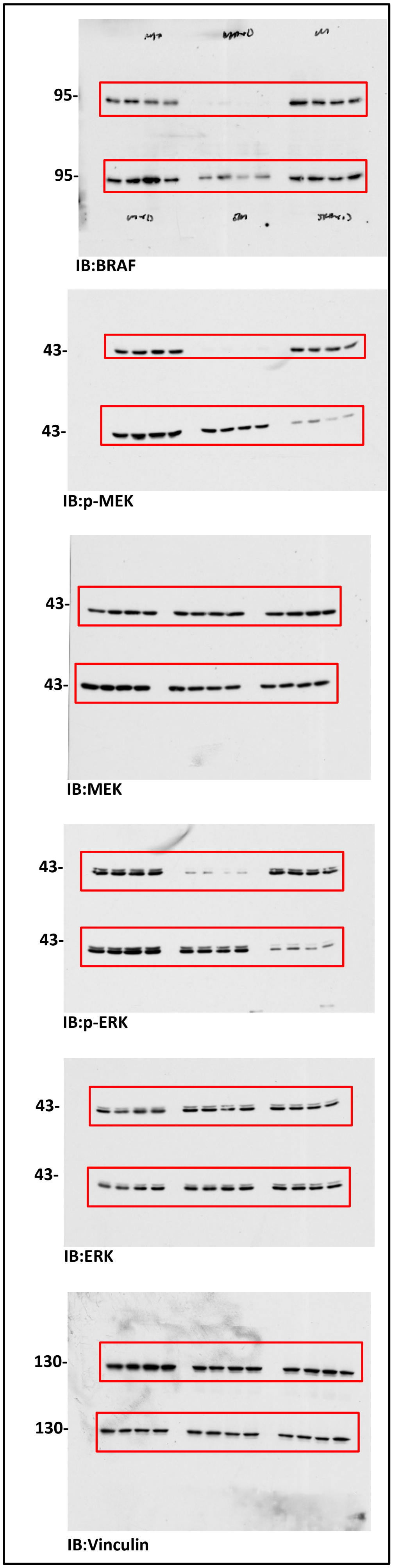

Uncropped immunoblots from Supplementary Figure 11

Supplementary Table 1. A list of primers used in this study

**Primer name**		**Primer sequence (5'-3')**
BRAF-AgeI-F	Forward	GCATACCGGTGCGGCGCTGAGCGGTG
BRAF-SalI-R	Reverse	GCATGTCGACTCAGTGGACAGGAAAC
BRAF-K164R	Sense	GAGTCTTCCTGCCCAACAGACAGAGGACAGTGG
	Antisense	CCACTGTCCTCTGTCTGTTGGGCAGGAAGACTC
BRAF-K473R	Sense	CATTTGGAACAGTCTACAGGGGAAAGTGGCATGG
	Antisense	CCATGCCACTTTCCCCTGTAGACTGTTCCAAATG
BRAF-K570R	Sense	GGATTACTTACACGCCAGGTCAATCATCCACAGAG
	Antisense	CTCTGTGGATGATTGACCTGGCGTGTAAGTAATCC
BRAF-K698R	Sense	GATTAATGGCAGAGTGCCTCAGAAAGAAAAGAGATGAGAGAC
	Antisense	GTCTCTCATCTCTTTTCTTTCTGAGGCACTCTGCCATTAATC
BRAF-K699R	Sense	GGCAGAGTGCCTCAAAAGGAAAAGAGATGAGAGAC
	Antisense	GTCTCTCATCTCTTTTCCTTTTGAGGCACTCTGCC
BRAF-K700R	Sense	CAGAGTGCCTCAAAAAGAGAAGAGATGAGAGACCAC
	Antisense	GTGGTCTCTCATCTCTTCTCTTTTTGAGGCACTCTG
BRAF-S365A	Sense	GAGACCGATCCTCAGCAGCTCCCAATGTG
	Antisense	CACATTGGGAGCTGCTGAGGATCGGTCTC
BRAF-S729A	Sense	CAAAAATTCACCGCAGTGCAGCAGAACCCTCCTTGAATCG
	Antisense	CGATTCAAGGAGGGTTCTGCTGCACTGCGGTGAATTTTTG
BRAF-P490A/P492A	Sense	GTTGAATGTGACAGCAGCTACAGCTCAGCAGTTACAAGC
	Antisense	GCTTGTAACTGCTGAGCTGTAGCTGCTGTCACATTCAAC
BRAF-P631A/F634A	Sense	CCAGAAGTCATCAGAATGCAAGATAAAAATGCATACAGCGCTCAGTCAGATGTATATG
	Antisense	CATATACATCTGACTGAGCGCTGTATGCATTTTTATCTTGCATTCTGATGACTTCTGG
BRAF-P655A	Sense	GAATTGATGACTGGACAGTTAGCTTATTCAAACATCAACAACAG
	Antisense	CTGTTGTTGATGTTTGAATAAGCTAACTGTCCAGTCATCAATTC
BRAF-S675A/P676A	Sense	GGGACGAGGATACCTGGCTGCAGATCTCAGTAAGG
	Antisense	CCTTACTGAGATCTGCAGCCAGGTATCCTCGTCCC
BRAF-P705A/P708A	Sense	CCTCAAAAAGAAAAGAGATGAGAGAGCACTCTTTGCCCAAATTCTCGC
	Antisense	GCGAGAATTTGGGCAAAGAGTGCTCTCTCATCTCTTTTCTTTTTGAGG
BRAF-P731A	Sense	CCGCAGTGCATCAGAAGCCTCCTTGAATCG
	Antisense	CGATTCAAGGAGGCTTCTGATGCACTGCGG
BRAF-P751A/P754A	Sense	GTCTATATGCTTGTGCTTCTGCAAAAACAGCCATCCAGGCAG
	Antisense	CTGCCTGGATGGCTGTTTTTGCAGAAGCACAAGCATATAGAC
ITCH-C832S	Sense	GTTACCCAGAAGCCATACTAGTTTTAACCGCCTGGAC
	Antisense	GTCCAGGCGGTTAAAACTAGTATGGCTTCTGGGTAAC
shBRAF	Sense	CCGGCAGATGAAGATCATCGAAATCTCGAGATTTCGATGATCTTCATCTGTTTTTG
	Antisense	AATTCAAAAACAGATGAAGATCATCGAAATCTCGAGATTTCGATGATCTTCATCTG
shBRAF^{resistant}	Sense	CACAGCCCTTCCGACCAGCAGACGAGGACCACAGAAATCAATTTGGGCAACGAG
	Antisense	CTCGTTGCCCAAATTGATTTCTGTGGTCCTCGTCTGCTGGTCGGAAGGGCTGTG
shJNK-1	Sense	CCGGGCAGAAGCAAGCGTGACAACACTCGAGTGTTGTCACGCTTGCTTCTGCTTTTTG
	Antisense	AATTCAAAAAGCAGAAGCAAGCGTGACAACACTCGAGTGTTGTCACGCTTGCTTCTGC
shJNK-2	Sense	CCGGAAAGGTTGTGTGATATTCCAACTCGAGTTGGAATATCACACAACCTTTTTTTTG
	Antisense	AATTCAAAAAAAAGGTTGTGTGATATTCCAACTCGAGTTGGAATATCACACAACCTTT
shPPP2CA-A	Sense	CCGGTGGAACTTGACGATACTCTAACTCGAGTTAGAGTATCGTCAAGTTCCATTTTTG
	Antisense	AATTCAAAAATGGAACTTGACGATACTCTAACTCGAGTTAGAGTATCGTCAAGTTCCA
shPPP2CA-B	Sense	CCGGACCGGAATGTAGTAACGATTTCTCGAGAAATCGTTACTACATTCCGGTTTTTTG
	Antisense	AATTCAAAAAACCGGAATGTAGTAACGATTTCTCGAGAAATCGTTACTACATTCCGGT
shPPP2R2A-A	Sense	CCGGTATGTCTCGCTTTGTGTTTCTCTCGAGAGAAACACAAAGCGAGACATATTTTTG
	Antisense	AATTCAAAAATATGTCTCGCTTTGTGTTTCTCTCGAGAGAAACACAAAGCGAGACATA
shPPP2R2A-B	Sense	CCGGAAATGACCTGTTACTGGGATCCTCGAGGATCCCAGTAACAGGTCATTTTTTTTG
	Antisense	AATTCAAAAAAAAATGACCTGTTACTGGGATCCTCGAGGATCCCAGTAACAGGTCATTT

Supplementary References

1. Wan, P. T. *et al.* Mechanism of activation of the Raf-MEK signaling pathway by oncogenic mutations of B-Raf. *Cell* **116**, 856–867 (2004).
2. Xu, Y., Chen, Y., Zhang, P., Jeffrey, P. D. & Shi, Y. Structure of a protein phosphatase 2A holoenzyme: insights into B55-mediated Tau dephosphorylation. *Mol. Cell* **31**, 873–885 (2008).
