# Supplementary material for: K27-linked ubiquitination of BRAF by ITCH engages cytokine response to maintain MEK-ERK signaling
Source: Nat Commun. 2019 Apr 23;10:1870. doi: 10.1038/s41467-019-09844-0 (PMC6478693; doi:10.1038/s41467-019-09844-0)
Supplement: Supplementary file 3 — Reporting Summary [file 41467_2019_9844_MOESM3_ESM.pdf]

nature research

Corresponding author(s): Lixin Wan
Last updated by author(s): 3/12/2019

Reporting Summary

Nature Research wishes to improve the reproducibility of the work that we publish. This form provides structure for consistency and transparency in reporting. For further information on Nature Research policies, see Authors & Referees and the Editorial Policy Checklist.

Statistics

For all statistical analyses, confirm that the following items are present in the figure legend, table legend, main text, or Methods section.

n/a	Confirmed	
☐	☒	The exact sample size (*n*) for each experimental group/condition, given as a discrete number and unit of measurement
☐	☒	A statement on whether measurements were taken from distinct samples or whether the same sample was measured repeatedly
☐	☒	The statistical test(s) used AND whether they are one- or two-sided<br>*Only common tests should be described solely by name; describe more complex techniques in the Methods section.*
☒	☐	A description of all covariates tested
☒	☐	A description of any assumptions or corrections, such as tests of normality and adjustment for multiple comparisons
☐	☒	A full description of the statistical parameters including central tendency (e.g. means) or other basic estimates (e.g. regression coefficient) AND variation (e.g. standard deviation) or associated estimates of uncertainty (e.g. confidence intervals)
☐	☒	For null hypothesis testing, the test statistic (e.g. *F*, *t*, *r*) with confidence intervals, effect sizes, degrees of freedom and *P* value noted<br>*Give P values as exact values whenever suitable.*
☒	☐	For Bayesian analysis, information on the choice of priors and Markov chain Monte Carlo settings
☒	☐	For hierarchical and complex designs, identification of the appropriate level for tests and full reporting of outcomes
☐	☒	Estimates of effect sizes (e.g. Cohen's *d*, Pearson's *r*), indicating how they were calculated

Our web collection on statistics for biologists contains articles on many of the points above.

Software and code

Policy information about availability of computer code

Data collection	The TCGA-SKCM RPPA data were retrieved from https://tcpaportal.org/tcpa/.
Data analysis	Graphpad Prism 7.0, ImageJ and ggplot2 (https://github.com/tidyverse/ggplot2)

For manuscripts utilizing custom algorithms or software that are central to the research but not yet described in published literature, software must be made available to editors/reviewers. We strongly encourage code deposition in a community repository (e.g. GitHub). See the Nature Research guidelines for submitting code & software for further information.

Data

Policy information about availability of data

All manuscripts must include a data availability statement. This statement should provide the following information, where applicable:

- Accession codes, unique identifiers, or web links for publicly available datasets
- A list of figures that have associated raw data
- A description of any restrictions on data availability

The data that support the findings of this study are available from the corresponding authors in reasonable request.

Field-specific reporting

Please select the one below that is the best fit for your research. If you are not sure, read the appropriate sections before making your selection.

☒ Life sciences ☐ Behavioural & social sciences ☐ Ecological, evolutionary & environmental sciences

For a reference copy of the document with all sections, see nature.com/documents/nr-reporting-summary-flat.pdf

Life sciences study design

All studies must disclose on these points even when the disclosure is negative.

Sample size	The chosen sample size are based on the numbers used for previous publications, which is most optimal to generate statistically significant results.
Data exclusions	No data were excluded for statistical analyses.
Replication	All attempts at replication were successful.
Randomization	The samples/cells were randomized to be examined. For doxycycline-induced in vivo mouse xenograft experiments, the mice were randomly grouped into normal diet and dox diet groups prior to dox treatment.
Blinding	Blinding was not relevant to the study because all cells/samples were analyzed in the same way.

Reporting for specific materials, systems and methods

We require information from authors about some types of materials, experimental systems and methods used in many studies. Here, indicate whether each material, system or method listed is relevant to your study. If you are not sure if a list item applies to your research, read the appropriate section before selecting a response.

Materials & experimental systems

n/a	Involved in the study	
☐	☒	Antibodies
☐	☒	Eukaryotic cell lines
☒	☐	Palaeontology
☐	☒	Animals and other organisms
☒	☐	Human research participants
☒	☐	Clinical data

Methods

n/a	Involved in the study	
☒	☐	ChIP-seq
☒	☐	Flow cytometry
☒	☐	MRI-based neuroimaging

Antibodies

Antibodies used	Anti-BRAF (9433, 1:2000), anti-CRAF (9422, 1:2000), anti-pT202/pY204-ERK1/2 (4370, 1:3000), anti-ERK1/2 (4695, 1:3000), anti-pS217/pS221-MEK1/2 (9154, 1:3000), anti-MEK1/2 (9122, 1:3000), anti-SAPK/JNK(9252, 1:2000), anti-pT183/pY185-SAPK/JNK (4668, 1:2000), anti-c-Jun (9165, 1:2000), anti-JunB (3753, 1:2000), anti-ubiquitin (3933, 1:1000), anti-RAS (3339, 1:2000), anti-p-S259-CRAF (9421, 1:2000), anti-K48-linkage ubiquitin (D9D5, 1:2000), Human TNFa Neutralizing (D1B4) mAb (7321), and anti-GST (2622, 1:2000) antibodies were purchased from Cell Signaling Technology. Anti-FLIP (H-150, 1:2000), anti-pS63-c-Jun (KM-1, 1:2000), anti-p-S621-Raf-1 (E-1, 1:2000), anti-c-Myc (9E10, 1:2000) and polyclonal anti-HA (Y-11, 1:2000) antibodies were purchased from Santa Cruz. Anti-TUBULIN (T-5168, 1:2000) and anti-VINCULIN (V-4505, 1:2000) antibodies were purchased from Bethyl Labs. Polyclonal anti-Flag antibody (F-2425, 1:2000), monoclonal anti-Flag (F-3165, 1:2000) antibody, anti-Flag agarose beads (A-2220), and anti-HA agarose beads (A-2095) as well as peroxidase-conjugated anti-mouse secondary antibody (A-4416, 1:3000) and peroxidase-conjugated anti-rabbit secondary antibody (A-4914, 1:3000) were purchased from Sigma. Anti-K27-linkage ubiquitin (ab181537, 1:2000) was purchased from Abcam. Monoclonal anti-HA antibody (MMS-101P, 1:2000) was purchased from Covance.
Validation	Antibody validation was deferred to the manufacturers and was supported by multiple publications.

Eukaryotic cell lines

Policy information about cell lines

Cell line source(s)	293T, HEK293, and A375 cell lines were obtained from ATCC. 1205Lu, WM1346, WM3918 cell lines were a gift from Dr. Meenhard Herlyn (The Wistar Institute). M245 was a gift from Dr. Antoni Ribas (UCLA Medical Center). IPC-298 was purchased from the Leibniz-Institut DSMZ (Germany). melan-a cell line was a gift from Dr. Wenyi Wei (BIDMC, Harvard Medical School).
Authentication	Cancer cell lines were used without further authentication.
Mycoplasma contamination	293T, M245, IPC-298, WM1346, WM3918, melan-a cell lines were tested negative for mycoplasma contamination.
Commonly misidentified lines (See ICLAC register)	No commonly misidentified cell lines were used in this study.

Animals and other organisms

Policy information about studies involving animals; ARRIVE guidelines recommended for reporting animal research

Laboratory animals	6-week-old male nude mice (NCRNU-M-M) were purchased from Taconic.
Wild animals	The study did not involve wild animals.
Field-collected samples	The study did not involve samples collected from the field.
Ethics oversight	All research involving animals was complied with protocols approved by the University of South Florida Animal Care and Use Committee.

Note that full information on the approval of the study protocol must also be provided in the manuscript.
